# Overall and serotype-specific dengue virus transmission intensity in Mexico, 2016–2023: A modelling study of case-notification data

**DOI:** 10.1371/journal.pmed.1004874

**Published:** 2026-09-03

**Authors:** Oliver S. Simmons, Anna Vicco, Ruth A. Martínez-Vega, José Ramos-Castañeda, Ilaria Dorigatti

**Affiliations:** 1 MRC Centre for Global Infectious Disease Analysis, School of Public Health, Imperial College London, London, United Kingdom; 2 Facultad de Ciencias Médicas y la Salud, Universidad de Santander, Bucaramanga, Colombia; 3 Centro de Investigaciones sobre Enfermedades Infecciosas, Instituto Nacional de Salud Pública, Cuernavaca, México; University of Glasgow, UNITED KINGDOM OF GREAT BRITAIN AND NORTHERN IRELAND

## Abstract

**Background:**

Mexico, and the Americas region more widely, have experienced increased dengue incidence in recent years, and there are concerns that the changing climate may enhance dengue virus (DENV) transmission in the future. Whilst previous studies have characterised spatial heterogeneities in the long-term average risk of DENV infection in Mexico, the extent to which DENV transmission changes year-to-year has not been investigated. Furthermore, the extent to which DENV’s four serotypes (DENV-1, DENV-2, DENV-3 and DENV-4) may differ in their transmissibility remains poorly characterised, both in Mexico and globally.

**Methods and findings:**

In this study, we characterised the spatial and temporal variations in DENV transmission intensity, as defined by the force of infection, across 27 states in Mexico. We analysed annual dengue case data reported to the National Epidemiological Surveillance System (SINAVE) in Mexico between 2016 and 2023, which comprised 833,629 probable or confirmed cases, using established catalytic models and a new, serotype-specific extension of these models that characterises differences in the transmission intensity of the different serotypes. We found evidence of large spatial, temporal, and serotype-specific heterogeneities in transmission intensity across Mexico. Serotype-specific force of infection estimates suggest that DENV-1 and DENV-2 have historically circulated at high levels across Mexico, with DENV-4 showing a low transmission intensity throughout the study period. We found evidence of increasing DENV-3 transmission intensity across some states in recent years, coinciding with large outbreaks. The extent to which the serotype-specific transmission intensity estimates generated in this study are affected by potential heterogeneities in case reporting across states and through time remains to be validated in future studies.

**Conclusions:**

This work quantifies serotype-specific differences in DENV transmission intensity using routinely collected case-notification data and demonstrates how extensive RT-PCR testing and new rapid diagnostic tests capable of discerning infecting serotypes can help to better understand the contributions of different serotypes to DENV transmission. The methods developed in this study contribute to the development of a better understanding of the past and current burden of dengue infection, which can help to refine assessments of the potential impact of existing and new interventions in future analyses.

## Author summary

**Why was this study done?:**

- Dengue, caused by infection with dengue virus, is an increasing public health threat globally, and is a cause of particular concern in Mexico and the Americas, where outbreaks have intensified in recent years.
- There are four serotypes of dengue virus, and due to the lack of serotype-specific data, modelling studies have traditionally assumed that the four serotypes are equally transmissible despite this hypothesis remaining to be validated.
- Mexico is one of the few countries to publish individual-level data on reported dengue cases, including the serotype of cases (when available), which provides new opportunities to generate evidence around the transmission intensity of the circulating serotypes.

**What did the researchers do and find?:**

- We applied mathematical models to analyse the data reported by disease surveillance, quantifying the intensity of dengue virus transmission in Mexico between 2016 and 2023.
- We developed a new model to estimate the transmission intensity of each of the circulating dengue virus serotypes separately.
- The intensity of dengue virus transmission varied by year, state, and serotype, with higher estimates obtained in the south of the country in 2023.

**What do these findings mean?:**

- We find evidence of substantial heterogeneity in dengue virus transmission intensity by state, year and serotype, which implies that the immunity profile of the population across the country is highly heterogeneous too.
- The methods developed in this paper can be applied to other countries, and the estimates can be used to inform the implementation of control interventions, surveillance strengthening, and public health decision-making.
- The model developed in this study assumes that dengue virus serotypes do not differ in their propensity to cause symptomatic disease, and that the observed serotype-specific data are representative of the serotypes circulating in the population. To date, these assumptions remain to be validated in Mexico and globally.

## Introduction

The annual number of suspected dengue cases in the Americas, whilst variable, has increased steadily since 2000 [1], with estimates suggesting that 16.7 (95% confidence interval [CI] [15.6, 17.7]) million infections and 7.6 (95% CI [4.8, 9.9]) million febrile cases occur on average each year in the region [2]. The principal vector of dengue virus (DENV), the mosquito *Aedes aegypti*, thrives in urban areas, breeding in small containers of water, and an increasingly urban global population therefore puts a growing number of people at risk of the disease. Increases in temperature and changing rainfall patterns, direct results of climate change, already have and will continue to impact the global distribution of infection and disease in the future [3,4], with currently inhospitable arid and temperate regions possibly becoming suitable to *Aedes* mosquitoes, and therefore dengue.

Changes in both urbanisation and climate are particularly acute in Mexico, where over 100 million people live in urban areas [5,6]. Mexico’s dengue burden has historically been greater in coastal and tropical regions in the south of the country, where the disease is endemic or hyper-endemic, and lower in the northern-central part of the country corresponding roughly to the Mexican Altiplano, as transmission here is limited by the high altitude, especially in areas higher than 1,700m above sea level (though exceptions to these generalities exist) [7,8]. Despite these limiting factors, Mexico reported approximately 278,000 cases of dengue in 2023, which represented the highest annual incidence in the country in the last decade (as recorded by the Pan American Health Organization (PAHO)), and the second highest number of cases in the Americas in 2023 after Brazil [1]. In 2024, dengue incidence in Mexico approximately doubled, with more than 558,000 cases reported to PAHO. Previous studies have suggested that the burden of dengue in Mexico may worsen further over the coming years due to climate change, although the extent and geographical distribution of any predicted change differs between models [3,9].

DENV transmission in Mexico is currently highly cyclical, both on an annual basis, with cases peaking between August and November each year during the rainy season, and on a year-to-year basis, with large outbreaks occurring every 3–4 years [1]. This inter-annual pattern of dengue is most likely a result of the complex population immune profiles that result from infection with DENV [10,11], which is made up of four, often co-circulating, serotypes denoted DENV-1, DENV-2, DENV-3 and DENV-4. Upon infection, each serotype confers long-term immunity to that serotype, and short-term immunity to heterologous serotypes, though estimates of the duration of this immunity vary [12]. This complex relationship between immunity and disease also makes evaluations of dengue’s disease burden difficult, and, due to differences in disease severity [13], the extent to which primary and post-primary (including tertiary and quaternary) infections are reported to surveillance can vary. Heterogeneities in the underreporting of cases across Mexico (as in any other country) and local differences in the sensitivity of dengue surveillance (driven, for instance, by differences in the historical trends of local DENV transmission) imply that the true burden of DENV infection is not necessarily reflected by the magnitude of the reported data [7,14].

The burden of dengue can, however, be estimated from the age-distribution of the reported case data through mathematical models. An important measure of DENV’s transmission intensity is the rate at which susceptible people become infected, known as the force of infection (FOI). The FOI of DENV can be estimated using a class of mathematical models referred to as catalytic models, first used to model DENV transmission dynamics by Ferguson and colleagues [15], using either seroprevalence or case-notification data [16–18]. In previous work, Imai and colleagues used two seroprevalence studies published in Mexico to estimate the nationwide DENV FOI to be within the range 0.035–0.037, assuming life-long immunity (and within the range 0.090–0.098 assuming immunity decay) [18]. On a sub-national level, Rodriguez-Barraquer and colleagues used case-notification data from 2000 to 2009 to estimate the FOI of DENV in 27 of Mexico’s 32 states, which ranged from 0.01 to 0.092 on average [19].

Previous modelling work on dengue using reported-case data typically assumes the equal circulation and transmissibility of all four serotypes [2,16,17,19,20], due to the absence of data on the number of cases caused by circulating serotypes. Whilst serotype-specific FOIs have been estimated, mainly from seroprevalence studies, none of these focussed on Mexico [15,18,21–24].

In light of the recent increases in dengue incidence in Mexico, in this study we aim to better characterise the transmission intensity of DENV across the country. We present a descriptive analysis of publicly available line-list dengue data recorded in Mexico between 2016 and 2023 and characterise the average DENV transmission intensity over all serotypes in the country using established catalytic models developed by Imai and colleagues and O’Driscoll and colleagues [16,17]. Finally, we developed a new serotype-specific catalytic model of DENV transmission intensity to quantify heterogeneities in the transmissibility of each serotype, enabling us to investigate potential drivers behind the incidence trends observed in Mexico over the last 8 years.

## Methods

### Data

#### Epidemiological data.

In Mexico, the reporting of suspected dengue cases by healthcare centres is mandatory, and dengue cases are recorded by the healthcare system through the National Epidemiological Surveillance System (*Sistema Nacional de Vigilancia Epidemiológica*; SINAVE). Anonymised line-list data on dengue cases in Mexico for 2020–2023 are available online from the Ministry of Health (*Secretaría de Salud*) [25], and data for 2016–2019 are available upon request from the *Instituto Nacional de Acceso a la Información* (INAI), folio 330026924000362. This study is reported as per the Reporting of Studies Conducted Using Observational Routinely-Collected Data (RECORD) guidelines (S1 Checklist).

The combined dataset comprised 1,062,240 entries, with information on the federal entity (*entidad federativa*; administrative level 1, from here on referred to as “state”, Fig A in S1 Text) and municipality (*municipio*; administrative level 2) of residence. We only considered probable or confirmed cases (of which there were 833,761) and excluded international cases (those with state of residence listed as either the United States of America [*Estados Unidos de América*], other Latin-American countries [*otros países de Latinoamérica*], or other countries [*otros países*]) and one person with an incorrect age entry from the analysis, leaving 833,629 cases. Case status (*estatus caso*) was defined as either probable (*probable*), confirmed (*confirmado*), or discarded (*descartado*), where confirmed cases indicate probable cases with DENV confirmed by laboratory techniques, and discarded cases are probable cases with DENV not confirmed by laboratory techniques [26]. The definition of a probable case can be found in the Standardised Procedures Manual for the Epidemiological Surveillance of Vector-Borne Diseases (*Manual de Procedimientos Estandarizados para la Vigilancia Epidemiológica de las Enfermedades Transmitidas por Vector*) [26].

All patients were categorised as either hospitalised (*hospitalizado*) or ambulatory (*ambulatorio*, i.e., non-hospitalised) (variable *tipo paciente*). Sex (*sexo*) was defined as either man (*hombre*) or woman (*mujer*), and death as either yes (*sí*) or no (*no*). The reverse transcription polymerase chain reaction (RT-PCR) test result (*resultado PCR*) was defined as either DENV-1, DENV-2, DENV-3, DENV-4, or no designated serotype (*sin serotipo aislado*). Not all probable dengue cases are tested for DENV infection, and not all those positive for DENV have their serotype categorised, with the proportion of probable cases selected for testing dependent on local DENV circulation according to surveillance and on the severity of the case [26]. The case fatality rate (CFR) was calculated as the proportion of confirmed and probable cases who were recorded as having died, and incidence as the total number of probable and confirmed cases per capita per year, with the population of each state each year calculated as below. For the descriptive analysis, uncertainty was calculated using exact binomial confidence intervals (Clopper-Pearson method).

#### Population data.

Population data at administrative levels 1 and 2 for Mexico were reconstructed from the 2015 intercensal survey and from the 2020 census [6,27]. For each state, we calculated the change in population of each 5-year age group ($A_{\text{1}},\;A_{\text{2}},\ldots ,\;A_{\text{1}\text{6}},\;$where *A*_1_ = 0–4 years old, *A*_2_ = 5–9 years old, …, *A*_15_ = 70–74 years old and *A*_16_ = 75+ years old) between 2015 and 2020 and reconstructed the yearly rates of population change, assuming that those whose age was listed as NA in the 2015 intercensal survey or in the 2020 census (0.07% of people in the 2015 intercensal survey and 0.22% [273,386/126,014,024] in the 2020 census) were distributed according to the age distribution of the rest of the population with known age in that year in each state. The population growth rate of age group $A_{j}$, $r_{j}$, j∈{1, 2…, 16}, was then used to estimate the population size of each age group in each state between 2015 and 2020, and we extrapolated the population size for 2021, 2022 and 2023 assuming the same growth trend, using the formula:


$$\begin{array}{c} \begin{array}{c} P_{j,t}=P_{j,t-\text{1}}\cdot r_{j} \end{array} \end{array}$$ (1)

where $P_{j,t}$ is the population of age group $A_{j}$ in year t, t∈T={2016, 2017, …, 2023}.

#### Serotype-specific case incidence.

We reconstructed the number of serotype-specific cases from the total number of cases reported by state and the respective proportions of cases positive for each serotype among hospitalised and non-hospitalised cases separately. To account for the fact that severity impacts the likelihood a person receives an RT-PCR test [26], we stratified cases with a designated serotype by hospitalisation status and calculated the prevalence of each serotype separately for hospitalised and non-hospitalised cases for each state and year. We then assumed that the prevalence of each serotype among the probable/confirmed cases without a designated serotype was the same as among those with a designated DENV serotype, and reconstructed the number of hospitalised and non-hospitalised cases for each serotype by multiplying the total number of hospitalised and non-hospitalised cases by, respectively, the serotype-specific proportion positive for each serotype among hospitalised and non-hospitalised cases. We then combined these estimates to generate the total reconstructed serotype-specific case incidence for each state and year.

We assumed that the serotype-specific distribution of cases with unknown hospitalisation status (0.006% [53/833,629] of probable and confirmed cases) was the same as was observed among non-hospitalised cases. Building on the available age-distribution of serotype-specific case data (Figs B and C in S1 Text), we assumed that the observed serotype proportions were constant over all ages. We reconstructed serotype-specific case incidence in a given state for the longest sequence of consecutive years for which there were more than 10 RT-PCR positive test results in each year. Furthermore, for a given state, we assumed that a serotype was circulating within the modelled years only if there were more than 10 RT-PCR positive cases for that serotype in at least one of the modelled years.

### Mathematical models

#### Non-serotype-specific models.

To estimate the FOI in each of Mexico’s states over the 8 years of data (2016–2023 inclusive), we used both time-constant and time-varying catalytic models, which assume that each serotype confers lifelong immunity against reinfection by the same serotype. The time-constant catalytic model, Model 1, developed by Imai and colleagues [16], assumes that the per-serotype FOI (denoted λ in the model) is constant throughout the 8 modelled years and historically prior to 2016. We also adapted the time-varying catalytic model developed by O’Driscoll and colleagues [17], denoted Model 2, to allow the per-serotype FOI to vary each modelled year (now denoted $\lambda_{t}$), and used five different historic FOI estimates for the years prior to 2016 ($\lambda_{H_{\text{1}}},\;\lambda_{H_{\text{2}}},\;\ldots ,\;\lambda_{H_{\text{5}}})$ to reconstruct the initial immunity profile of the population at the start of the time-series data ($\lambda_{H_{\text{1}}}$ is applied to the 20 years directly before the start of the study period, 1995–2015, $\lambda_{H_{\text{2}}}$ to the 20 years prior to that [1975–1995], and similarly for $\lambda_{H_{\text{3}}}$, $\lambda_{H_{\text{4}}}$ and $\lambda_{H_{\text{5}}}$). Both Model 1 and Model 2 assume that the four DENV serotypes are equally transmissible and that tertiary and quaternary cases are not symptomatic, and hence not reported (full model equations for Model 2 can be found in S1 Text).

In both models, the reported incidence of dengue cases in age group$\;\tilde{A_{j}}\;{(\tilde{A_{j}}=\;A}_{j}\;\forall \;j\in \left\{\text{2},\;\text{3},\ldots ,\;\text{1}\text{6}\right\},\;\tilde{{\;A}_{\text{1}}}=$ 1–4-year-olds) in year t, $D_{j,\;t}$, can be described by equation (2) below. Model 1 assumes that the reporting rate of primary infections is smaller or equal to the reporting rate of secondary infections, whereas Model 2 relaxes this assumption. Model 2 also allows for children under 15 years old to report at a different rate to people older than 15 years to reflect potential differences in the diagnosis and reporting of dengue cases through paediatricians.


$$\begin{array}{c} D_{j,t}=\tilde{\chi_{j}}\left(\rho \left(\sum_{a\;\in \;\tilde{A_{j}}}I_{\text{2},\;a,\;t}\right)+\gamma \left(\sum_{a\;\in \;\tilde{A_{j}}}I_{\text{1},a,t}\right)\right)\frac{\tilde{P_{j,t}}}{w_{j}} \end{array}$$ (2)

In equation (2), $I_{\text{1},a,t}$ is the annual incidence of primary dengue infection in age a in year t, $I_{\text{2},\;a,t}$ the annual incidence of secondary dengue in age a in year t, $\tilde{P_{j,t}}$ the population of $\tilde{A_{j}}$ in year t reconstructed as defined in equation (1), ρ the reporting rate of secondary cases, γ the reporting rate of primary cases (defined as $\tilde{\gamma }\cdot \rho$ in Model 1), $w_{j}$ the width of age group $\tilde{A_{j}}$ (e.g., for $\tilde{A_{\text{2}}}$, the age group containing 5–9 year olds,$w_{j}=\left|\tilde{A_{j}}\;\right|=\text{5}$), and $\tilde{\chi_{j}}$ a multiplier that allows children under 15 years old to report at a different rate than people older than 15 years in Model 2 (which was fixed to 1 in Model 1). We excluded cases in children under one year old from the analysis due to maternally transferred immunity [28], and modified $\tilde{{\;A}_{\text{1}}}$ and $\tilde{P_{\text{1},t}}$ accordingly (in the case of $\tilde{P_{\text{1},t}}$, using the proportion of 0-year-olds among children aged 0–4 years in the 2020 census [6]).

As a sensitivity analysis, and in line with O’Driscoll and colleagues [17], we tested the assumption that the FOI was constant-in-time for all years prior to 2016 (Model 3), a stricter assumption on the historic FOI than used in Model 2. We assigned *Normal(0,1)* prior distributions, with parameters constrained to be positive and at most 1 (or at most 0.25 in the case of per-serotype FOI estimates, see Table A in S1 Text), to all parameters in Models 1–3, apart from the scaling factor used in Models 2 and 3, $\tilde{\chi_{j}}$, which was assigned a *Normal(1,1)* prior distribution (with $\tilde{\chi_{j}}$ constrained to be positive and such that reporting rates were never above one). We performed model selection using: the deviance information criterion (DIC), the widely-applicable information criterion (WAIC), and leave-one-out information criterion (LOOIC) [29–31].

#### Serotype-specific model.

We developed a variation of Model 2 to allow serotype-specific time-varying FOIs, where $\lambda_{t}^{\omega }$ denotes the proportion of the total FOI attributable to each serotype (ω∈Ω={1, 2, 3, 4} denoting the 4 serotypes, t∈T denoting the year, with T varying depending on the data available). Equations (3)–(12) describe this new model (Model 4), where S(a,t) denotes the proportion of the population of age a susceptible at the end of year t, MO(a,t,ω) the proportion of the population monotypically infected with DENV serotype ω at age a at the end of year t, MU(a,t) the proportion of the population multitypically infected at age a at the end of year t, $I_{\text{1}}(a,t,\omega )$ the annual primary incidence of DENV serotype ω in age a in year t, and $I_{\text{2}}(a,t,\omega )$ the annual secondary incidence of DENV serotype ω in age a in year t. In equations (3)–(12), Ω denotes the set of DENV serotypes, Ω\{ω} the set of all serotypes excluding serotype ω. f(a, ω) is a function that defines the historical force of infection of serotype ω experienced by an individual of age a prior to 2016.


$$\begin{array}{c} S(a,\text{0})=\text{exp}\left(-\left(\sum_{\omega \;\in \;\Omega }f(a,\;\omega )\right)\right) \end{array}$$ (3)

$$\begin{array}{c} MO(a,\text{0},\omega )=\text{exp}\left(-\left(\sum_{i\;\in \;\Omega \backslash \{\omega \}}f(a,\;i)\right)\right)\left(\text{1}-\text{exp}\left(-f(a,\;\omega )\right)\right) \end{array}$$ (4)

$$\begin{array}{c} MU(a,\text{0})=\text{1}-S(a,\text{0})-\sum_{\omega \;\in \;\Omega }MO(a,\text{0},\omega ) \end{array}$$ (5)

where ∀ ω∈Ω,


$$\begin{array}{c} f(a,\;\omega )=\;\sum_{k\;=\;\text{1}\;}^{\text{5}}\sigma_{H_{k}}\lambda_{H_{k}}^{\omega }{\text{1}}_{\left\{a\;>\;\text{2}\text{0}(k-\text{1})\right\}}\text{min}(a-\text{2}\text{0}(k-\text{1}),\;\text{2}\text{0}) \end{array}$$ (6)

For t∈T,ω∈Ω,a≠0,


$$\begin{array}{c} S(a,t)=S(a-\text{1},t-\text{1})-\left(\sum_{\omega \;\in \;\Omega }\sigma_{t}\lambda_{t}^{\omega }\right)S(a-\text{1},t-\text{1}) \end{array}$$ (7)

$$\begin{array}{c} MO(a,t,\omega )=MO(a-\text{1},t-\text{1},\omega )+\sigma_{t}\lambda_{t}^{\omega }S(a-\text{1},t-\text{1})-\left(\sum_{\text{i }\in \;\Omega \backslash \{\omega \}}\sigma_{t}\lambda_{t}^{i}\right)MO(a-\text{1},t-\text{1},\omega ) \end{array}$$ (8)

$$\begin{array}{c} MU(a,t)=MU(a-\text{1},t-\text{1})+\sum_{\omega \;\in \;\Omega }\sigma_{t}\lambda_{t}^{\omega }\left(\sum_{\text{i }\in \;\Omega \backslash \{\omega \}}MO(a-\text{1},\;t-\text{1},\;i)\right) \end{array}$$ (9)

For t∈T,ω∈Ω,∀a,


$$\begin{array}{c} \begin{array}{c} I_{\text{1}}(a,t,\omega )=\sigma_{t}\lambda_{t}^{\omega }S(a,t-\text{1}) \end{array} \end{array}$$ (10)

$$\begin{array}{c} I_{\text{2}}(a,t,\omega )=\sigma_{t}\lambda_{t}^{\omega }\left(\sum_{\text{i }\in \;\Omega \backslash \{\omega \}}MO(a,\;t-\text{1},\;i)\right) \end{array}$$ (11)

The serotype-specific number of cases reported in age group $\tilde{A_{j}}$ during year t for serotype ω, $D_{j,t}^{\omega }$, was modelled as:


$$\begin{array}{c} D_{j,t}^{\omega }=\tilde{\chi_{j}}\;\left(\rho \left(\sum_{a\;\in \;\tilde{A_{j}}}I_{\text{2},\;a,\;t}^{\omega }\right)+\gamma \left(\sum_{a\;\in \;\tilde{A_{j}}}I_{\text{1},a,t}^{\omega }\right)\right)\frac{\tilde{P_{j,t}}}{w_{j}}\; \end{array}$$ (12)

where ρ represents the reporting rate of secondary cases, γ the reporting rate of primary cases, $\tilde{P_{j,t}}\;$the population of age group $\tilde{A_{j}}$ in year t (as defined above), $w_{j}$ the width of age group $\tilde{A_{j}}$, and where $\tilde{\chi_{j}}$ is, as above, a multiplier that allows children under 15 years old to report at a different rate than people older than 15 years ($\tilde{\chi_{j}}=\chi$ if j≤3, which we estimate, and $\tilde{\chi_{j}}=\text{1}$ otherwise).

We assigned a Dirichlet prior distribution to $\lambda_{t}^{\omega }$ at each timestep and included a scaling factor $\sigma_{\text{t}}$ ($\sigma_{H_{k}}$ for the historic $\lambda_{H_{k}}^{\omega }$’s) to allow the total FOI to be below 1, which was assigned a *Uniform(0,1)* prior distribution. Prior distributions for the other parameters were the same as for Model 2.

#### Model fitting.

We fitted our models to the observed age-stratified case-notification data (Models 1–3) and to the age-stratified serotype-specific case incidence data reconstructed from the observed case-notification data (Model 4). We assume that the total number of cases (over the study period for Model 1 and over each year for Models 2–3) was Poisson distributed and that the age-specific number of cases was multinomially distributed. Similarly, for Model 4, we assume that the yearly total number of cases (over all age groups) for each serotype was Poisson distributed, and that for each serotype the yearly age-specific distribution of cases was multinomially distributed. We report the median of the posterior distribution of the estimated parameters and the 95% credible interval (CrI) to show the uncertainty.

All models were fitted to the data using the Hamiltonian Monte Carlo method implemented using Stan (*RStan* version 2.32.7) [32,33]. For each state, models were run using 3 chains, each with 6,000 iterations, of which the first 1,000 were considered warm-up. The chains were considered to have successfully converged if the diagnostic value *Rhat* was below 1.01 and the bulk effective sample size (ESS) was over 300 for all parameters, as per Stan guidelines [33]. We excluded 5 states from our modelling analyses: Baja California, Ciudad de México, Chihuahua, Tlaxcala and Zacatecas, as there was no evidence of sustained transmission over the study period in these states.

### Ethics

This study is a secondary analysis of anonymised data that is either publicly available or available upon request, obtained from the National Epidemiological Surveillance System of the Mexican Ministry of Health. In accordance with the criteria of the CIOMS-WHO Guideline 12 and Article 17 of local health legislation [34,35], the project is classified as research involving minimal risk (or no risk) and is therefore exempt from the requirement to obtain individual informed consent.

### Coding

Data cleaning, manipulation, analysis and visualisation were conducted in R version 4.5.2 [36] using the packages *rstan, tidyverse*, *sf*, *MetBrewer, patchwork* and *binom*. Model comparison was performed with the *loo* R package [31].

## Results

### Descriptive analysis

**Fig 1:**
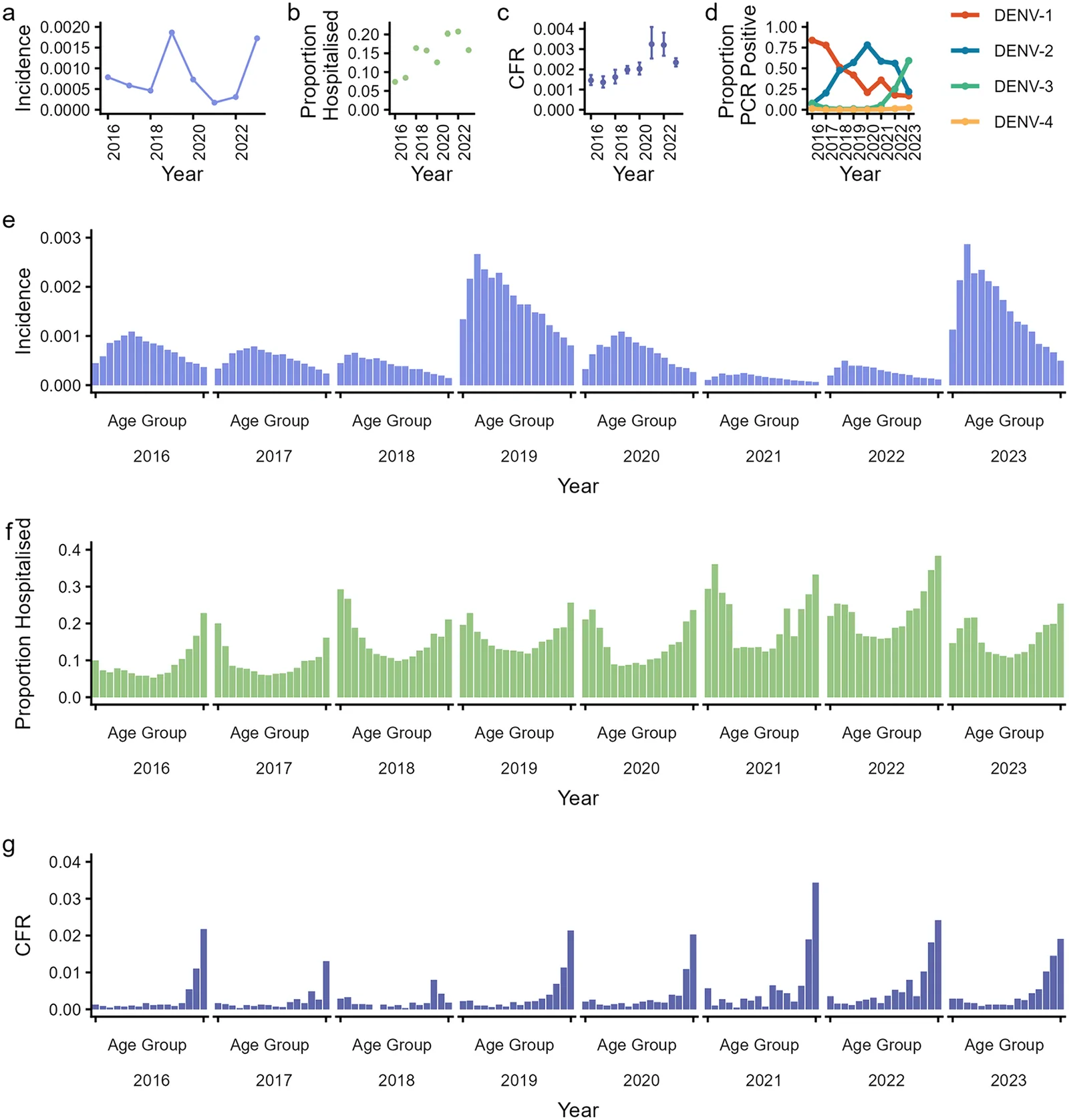
National incidence of reported probable or confirmed cases, hospitalisation, and case fatality ratio (CFR). **(a)** Yearly reported case incidence; **(b)** yearly proportion of cases hospitalised among those reported **(c)** yearly CFR, calculated as the yearly number of deaths among those with dengue divided by the yearly number of reported dengue cases, **(d)** proportion of serotype-specific cases positive for each DENV serotype, **(e)** yearly age distribution of case incidence, **(f)** yearly and age-specific proportion of cases hospitalised and **(g)** age-specific CFR by year. Ages were split into 5-year bands (0–4 years, 5–9 years, up to 70–74 years and 75+ years). Plots b and c include the 95% exact binomial confidence interval for each point.

Of the 833,629 probable or confirmed dengue cases in Mexico between 2016 and 2023, 53.9% (449,127) were in females, and the reported incidence among females was significantly higher than amongst males (0.874 versus 0.786 probable or confirmed case per 1,000 people per year, *p*-value <0.001, chi-squared test). Reported dengue incidence varied over time, with the highest incidence rates occurring during the outbreak years of 2019 and 2023 (1.86 and 1.73 cases per 1,000 people, respectively) (Fig 1a and 1e). Nationally, the median age of dengue cases ranged from 23 years in 2018 to 28 years in 2020, and the median age of reported cases was higher in females (27 years) than males (23 years).

The severity of dengue during the study period varied by age and location. At the national level, the proportion of hospitalised cases by age followed a “U” shape, with a greater proportion of hospitalised cases among the young and the elderly (Fig 1f). Nationally, over the entire study period, 14.2% (118737/833576; 95% exact binomial CI [14.2%, 14.3%]) of confirmed and probable cases were hospitalised. Hospitalisation rates were highest in the oldest age groups and significantly higher among males 75 years old and over than among females in the same age group; 27.6% (1297/4697; 95% CI [26.3%, 28.9%]) compared to 22.2% (1160/5216; 95% CI [21.1%, 23.4%]; *p*-value <0.001, chi-squared test). Geographically, states in the south and north-east of Mexico tended to admit the highest proportion of dengue cases to hospital (see Fig D in S1 Text), with the greatest proportion of probable or confirmed cases hospitalised in Chiapas (30.6%; 13671/44605; 95% CI [30.2%, 31.1%]), Tlaxcala (26.8%; 26/97; 95% CI [18.3%, 36.8%]) and Guerrero (24.9%; 12311/49388; 95% CI [24.5%, 25.3%]).

The overall case fatality ratio (CFR) during the study period was 0.203% (95% CI [0.194%, 0.213%]), with 1,695 deaths reported among those with confirmed or probable dengue. CFRs were highest in 2021 (0.325%; 71/21866; 95% CI [0.254%, 0.409%]) and 2022 (0.321%; 127/39611; 95% CI [0.267%, 0.381%]) (Fig 1c). The highest CFRs were observed in older age groups (Fig 1g), and, mirroring trends seen among hospitalisation rates, older males had a higher CFR than older females. Males over the age of 75 had the highest CFR of any group, 2.49% (117/4698; 95% CI [2.06%, 2.98%]), while the lowest CFR was amongst males aged 20–25 (0.0708%; 29/40941; 95% CI [0.0474%, 0.107%]). Regionally, the highest CFRs were recorded in states in north-west Mexico such as Baja California (0.591%; 3/508; 95% CI [0.122%, 1.72%]), Sonora (0.524%; 62/11843; 95% CI [0.402%, 0.671%]) and Chihuahua (0.929%; 3/323; 95% CI [0.192%, 2.69%]) (see Fig D in S1 Text).

**Fig 2:**
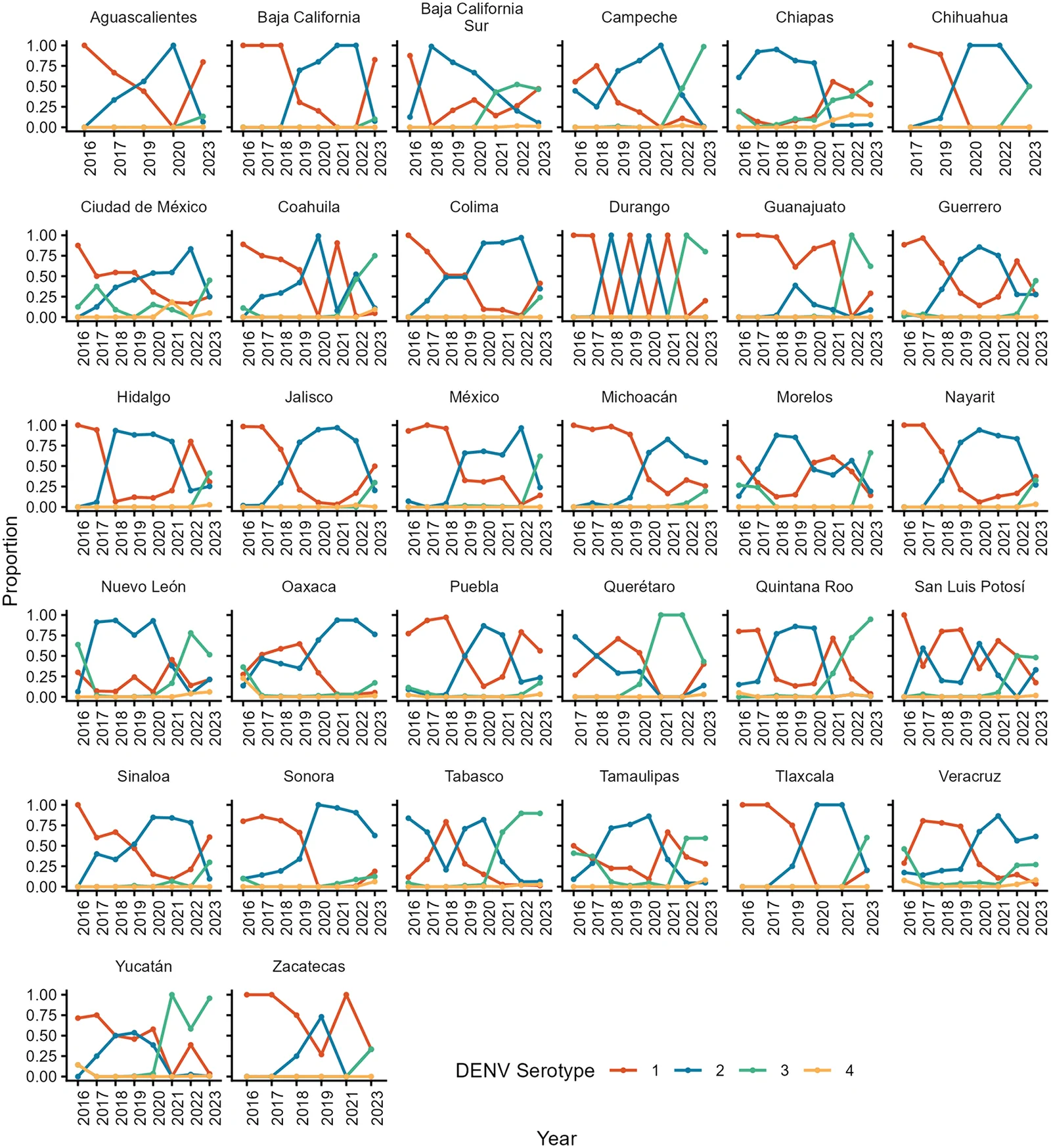
Spatiotemporal heterogeneity of DENV serotypes. Proportion of RT-PCR-positive dengue patients positive for each of the four serotypes of DENV. Each panel represents a different state (by year), and each serotype is shown in a different colour. Note: México here refers to the State of México (one of Mexico’s 32 states), not the country itself.

Among all cases, 85,529 (10.3% of all probable or confirmed cases) people tested RT-PCR positive for one of the four DENV serotypes between 2016 and 2023. 35.6% (30,473) of these were positive for DENV-1, 45.5% (38,894) for DENV-2, 18.2% (15,535) for DENV-3 and 0.7% (627) for DENV-4. Post-2021, DENV-3 circulation steadily increased in the south of Mexico, particularly in states in the Yucatán peninsula such as Yucatán, Quintana Roo and Campeche, where DENV-3 accounted for 95.6% (4,551/4,759), 94.8% (2,293/2,420) and 98.5% (603/612) of all RT-PCR-confirmed dengue cases in 2023, respectively (Fig 2).

**Fig 3:**
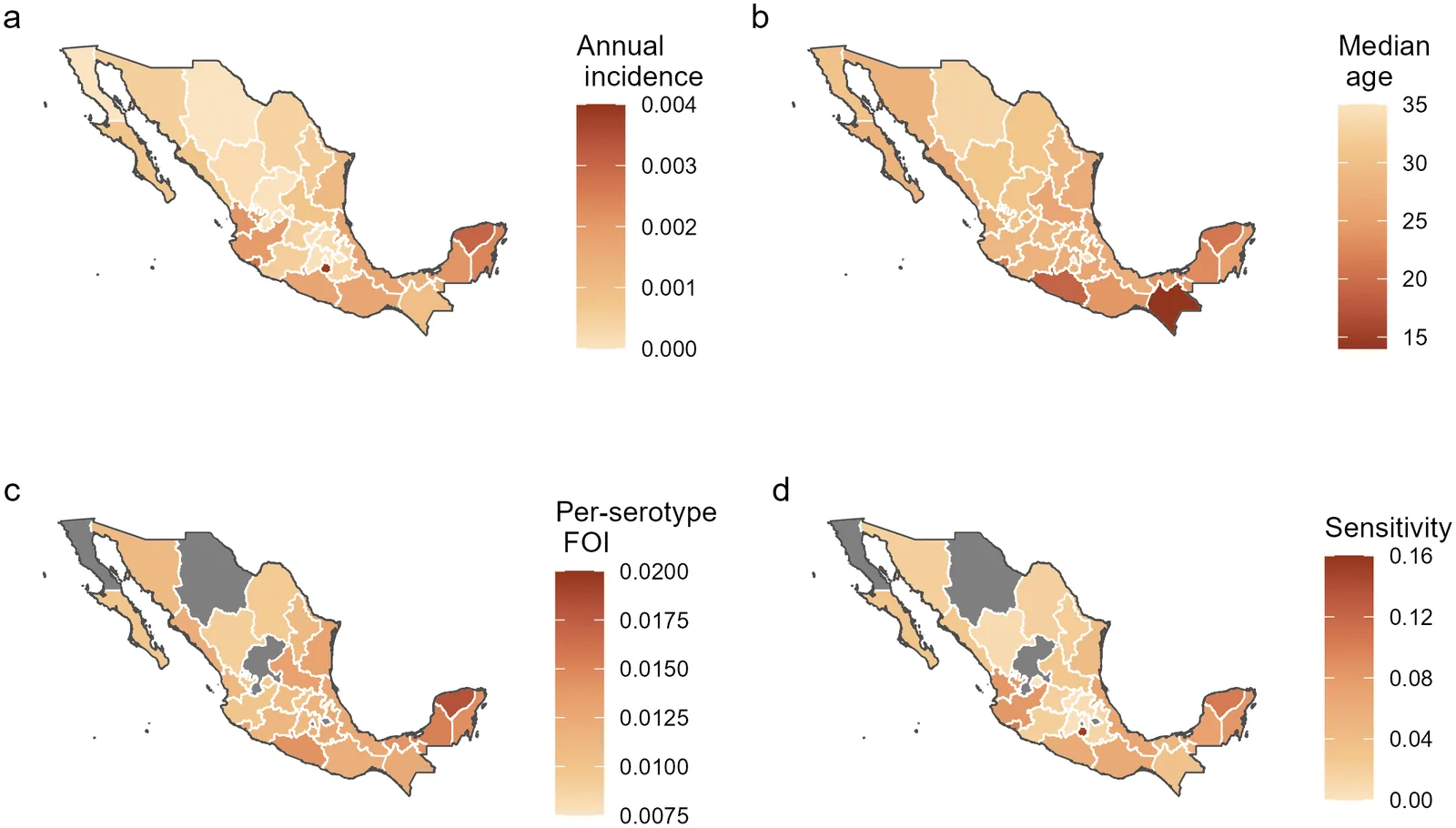
Spatial heterogeneities in the age of cases, incidence, force of infection and sensitivity of surveillance during 2016–2023. Dengue incidence per person per year by state over the period 2016–2023 **(a)**, median age of reported probable/confirmed dengue case by state **(b)**, median estimated time-constant per-serotype FOI (λ) for each of the 27 modelled states in Mexico between 2016 and 2023, estimated using a non-serotype specific model (Model 1) fitted to line-list case data of probable and confirmed dengue cases **(c)** and estimated sensitivity of surveillance, measured as the number of reported probable/confirmed dengue cases divided by the median expected number of infections from Model 1 **(d)**. Fig 3c and 3d shows the results of modelling work, with Baja California, Ciudad de México, Chihuahua, Tlaxcala and Zacatecas in grey because they were excluded due to low dengue incidence. The shapefiles used to generate the maps in the figure above were obtained from GADM (https://gadm.org/) and can be accessed by going to https://gadm.org/download_country.html and selecting “Mexico” from the drop-down menu.

Dengue incidence was highly regionally heterogeneous during the reporting period (Fig 3a). 29.5% of the overall reported dengue case burden between 2016 and 2023 was concentrated in Jalisco and Veracruz, Mexico’s third and fourth largest states by population, which were worst affected during the 2019 outbreak, reporting, respectively, 74,023 and 43,611 dengue cases that year. The 2023 outbreak struck states further to the south, such as Yucatán, Morelos, Campeche and Quintana Roo, which reported 44,646, 31,946, 8,934 and 19,825 cases, respectively, corresponding to incidences of 18.1, 15.9, 9.45, and 9.39 cases per 1,000 people (Fig E in S1 Text).

Morelos, Yucatán and Colima reported the highest dengue incidences between 2016 and 2023 (3.89, 3.03 and 2.72 cases per 1,000 people per year, respectively) (Fig 3a), while the lowest dengue burdens were seen in Tlaxcala and the northern states of Chihuahua, Baja California and Zacatecas, which reported 97, 323, 508 and 458 confirmed or probable cases of dengue over the entire 8-year period, respectively. These represented the lowest per-capita reported incidences in the country, along with Ciudad de México. States in the south of the country showed a lower median age of reported cases than states in the centre and north (Fig 3b and Table B in S1 Text).

### Non-serotype-specific models

#### Time-constant model.

Model 1 converged for all 27 states modelled. Fig 3c shows the per-serotype FOI (λ) estimates from Model 1 by state, showing large variations from 0.0092 (95% CrI [0.0086, 0.0097] in Durango to 0.0182 (95% CrI [0.0179, 0.0184] in Yucatán. Overall, our results suggest a higher FOI in the south of the country, particularly in the Yucatán peninsula. Estimated secondary reporting rates (ρ) varied from 0.0051 (95% CrI [0.0046, 0.0058]) in Querétaro to 0.3245 (95% CrI [0.3207, 0.3284] in Morelos, with low estimated secondary reporting rates often accompanying low estimates of per-serotype FOI. We estimate that the sensitivity of surveillance in Mexico during 2016–2023, defined as the total number of reported probable and confirmed cases divided by the total model-derived number of infections, varied from between 0.210% (95% CrI [0.196%, 0.229%]) of infections detected in Aguascalientes to 15.3% (95% CrI [15.2%, 15.4%]) of infections detected in Morelos (Fig 3d).

#### Time-varying models.

The model which allowed the historical FOI to vary over time (Model 2) outperformed the model with a constant historical FOI (Model 3) by DIC, WAIC and LOOIC in, respectively, 27, 23 and 25 states out of a total of 27 (Table C in S1 Text). As a result, we present the results of Model 2 below (which converged for all states according to the tests set out in Methods). We estimate lower reporting rates for primary infections compared to secondary infections in all 27 states, and the reporting rate of children under 15 to be higher than that of people older than 15 in all states but four (Coahuila, Guanajuato, Durango and Querétaro), with χ ranging from 0.795 (95% CrI [0.574, 1.084]) in Querétaro to 1.364 (95% CrI [1.284, 1.447]) in Puebla (Fig F in S1 Text).

**Fig 4:**
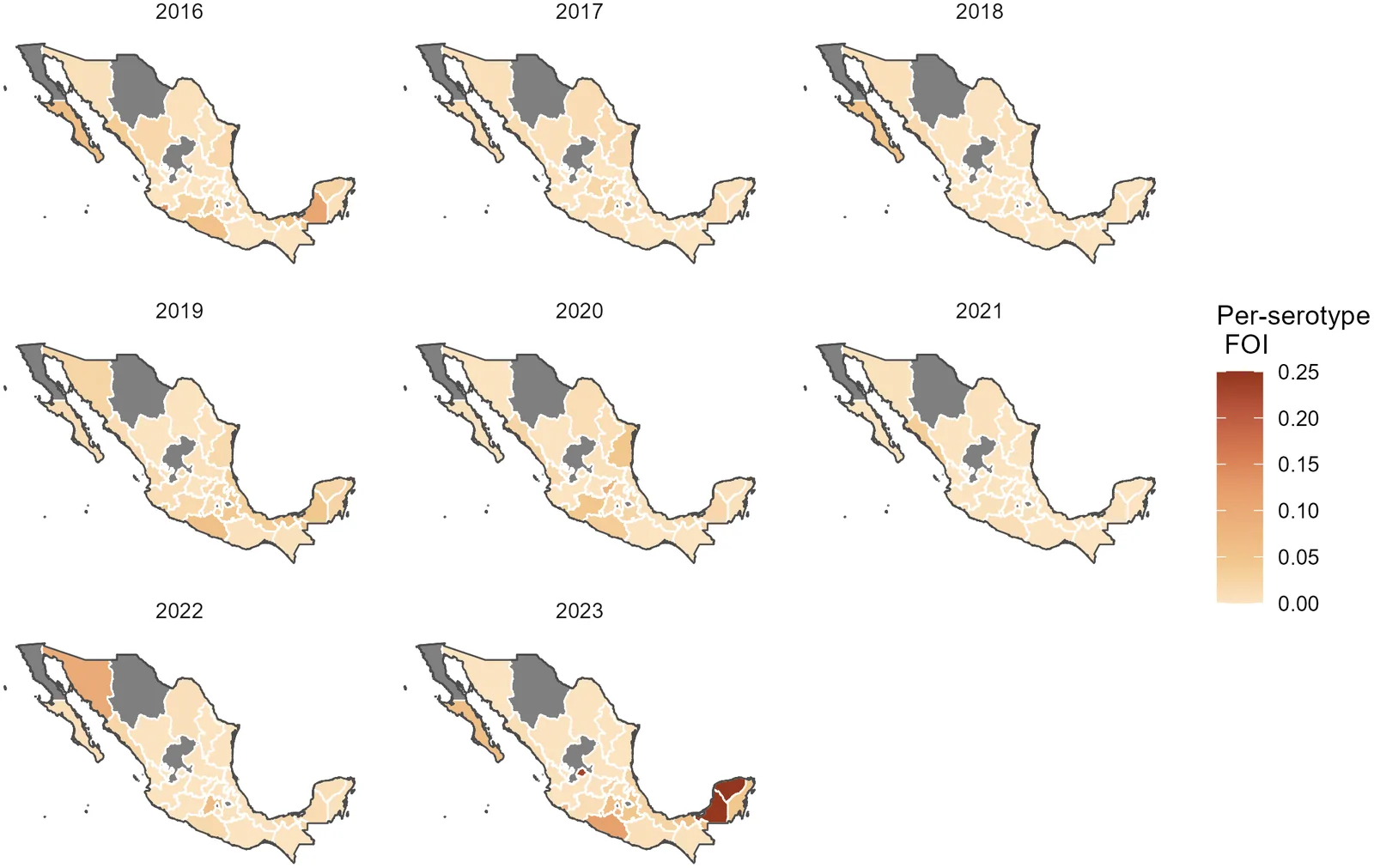
Spatiotemporal heterogeneity in annual state-level per-serotype FOI. Map of the median estimated time-varying per-serotype FOI ($\lambda_{t}$) for each of the 27 modelled states in Mexico between 2016 and 2023, obtained using a non-serotype-specific model (Model 2), which allows for a variable historic FOI and different reporting rates for children under 15 years, fitted to line-list case data of probable and confirmed dengue cases. The shapefiles used to generate the maps in the figure above were obtained from GADM (https://gadm.org/) and can be accessed as described above.

Fig 4 shows the median per-serotype FOI estimate, $\lambda_{\text{t}}$ (by definition assumed to be the same for all four serotypes) from Model 2, which is highly heterogeneous both in space and time. For instance, in Morelos, which is the state with the highest per-capita number of detected dengue cases over the reporting period, the median estimated $\lambda_{t}$ varied from 0.0206 (95% CrI [0.0187, 0.0226]) in 2016 to 0.0051 (95% CrI [0.0046, 0.0056]) in 2022, before rising again to 0.0804 (95% CrI [0.0734, 0.0879]) in 2023. During 2023, the most recent year modelled, estimates of $\lambda_{t}$ ranged from a median of 0.0003 (95% CrI [0.0002, 0.0004]) in Nuevo León in the north-east of the country and 0.00030 (95% CrI [0.00029, 0.00032]) in Jalisco in the west, to 0.1155 (95% CrI [0.1077, 0.1231]) in Guerrero and to as high as 0.2492 (95% CrI [0.2459, 0.2500]) in Yucatán and 0.2468 (95% CrI [0.2350, 0.2499]) in Campeche in the south-east.

Our model estimates that 10 out of 27 states had their highest DENV FOI during 2023. The states that saw the highest estimates of $\lambda_{t}$ over the 8-year period were not, however, geographically clustered: Yucatán, Campeche and Guerrero in the south; Aguascalientes, Querétaro and Morelos in the centre; Sonora in the north; and Colima in the west. In contrast, lower FOI estimates were clustered in the centre of the country, especially in Guanajuato, San Luis Potosí and Durango.

### Serotype-specific model

Durango was not included in the serotype-specific analysis as only DENV-1 was circulating throughout the period studied. The limited number of cases positive by RT-PCR for one of DENV’s serotypes in the data meant that we only were able to model all 8 years for 11/26 states, and a varying number of years for the other 15. We fitted the serotype-specific model in 8 states that had all four DENV serotypes co-circulating, 15 states in which DENV-1, DENV-2 and DENV-3 were co-circulating, and three states that only experienced circulation of DENV-1 and DENV-2. We reconstructed the age-distribution of the serotype-specific data (see Methods) using the serotype-specific model in 25 states (with the model successfully converging in all these states). For estimates of γ, ρ and χ (estimated primary and secondary reporting rates and paediatric reporting multiplier), see Fig G in S1 Text.

**Fig 5:**
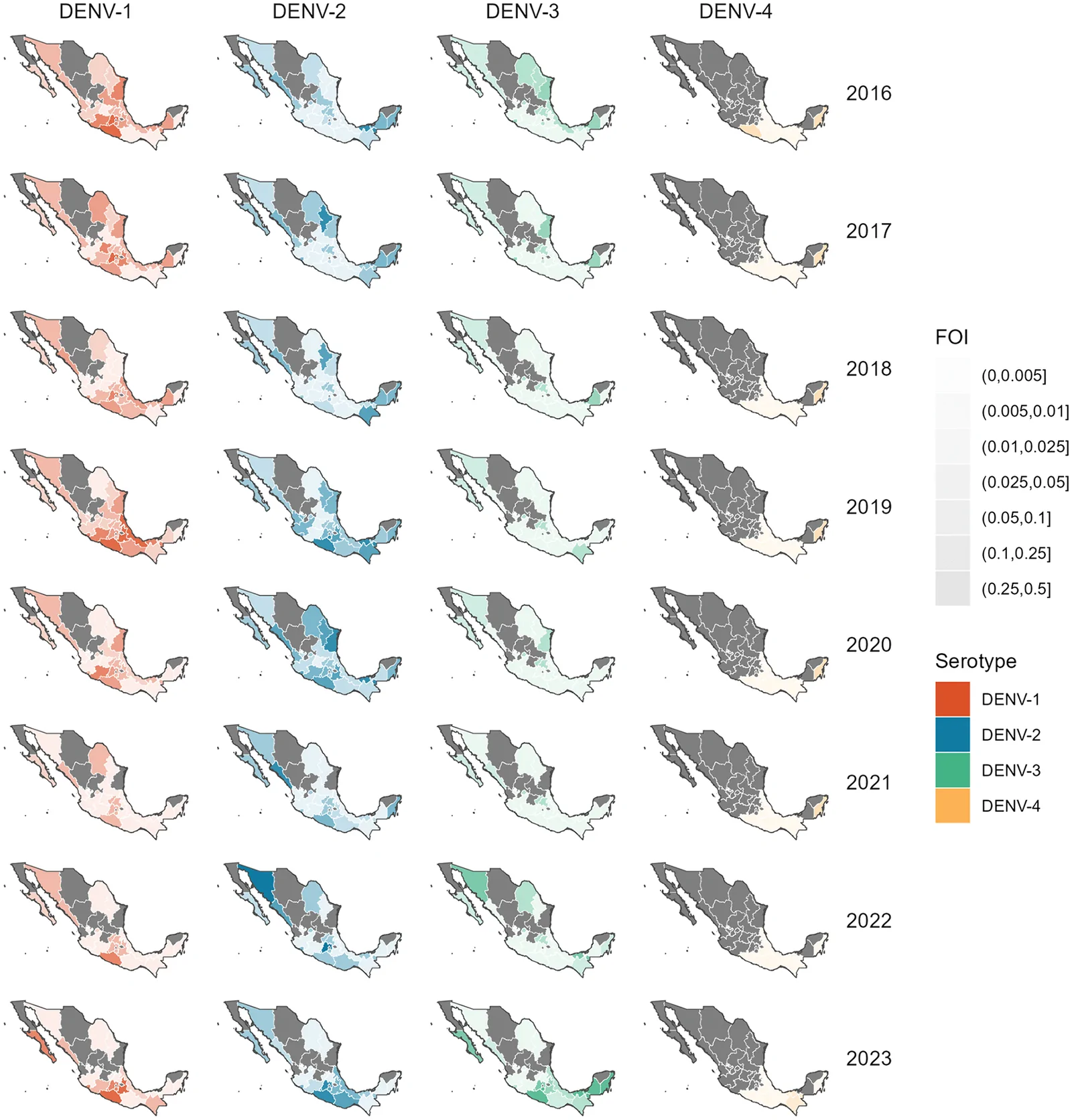
Annual serotype-specific force of infection (FOI) estimates by state. Serotype-specific FOI by state for each of the modelled years, grouped into FOI bands. States are shown in grey if there were no estimates generated for a given serotype and year, either due to the serotype being assumed to not be in circulation, the year being after the modelled period, or due to the state not being modelled. For states whose first modelled year was post 2016, the historical FOI estimate for the 20 years prior to the first modelled year is shown. The shapefiles used to generate the maps in the figure above were obtained from GADM (https://gadm.org/) and can be accessed as described above.

We found that the serotype with the greatest transmission intensity varied between states within a given year; in 2023, for example, DENV-1 had the largest FOI estimate in 9 states, DENV-2 the highest in 4 states and DENV-3 in 8 states. Each serotype’s transmission intensity also varied by year, with the highest estimated DENV-1 FOIs in 2016, 2019 and 2023, and the highest DENV-3 FOI estimates in either 2022 or 2023, clustered regionally in the south and to a lesser extent in the north of the country (Fig 5). The years in which DENV-2 transmission intensity was greatest varied by state, and DENV-4 had the lowest estimated transmission intensity of the four serotypes, with its median estimated FOI only rising above 0.01 in Guerrero in 2016 (low estimated DENV-4 FOIs for 2016–2023 were not preceded by high historic DENV-4 FOI estimates).

**Fig 6:**
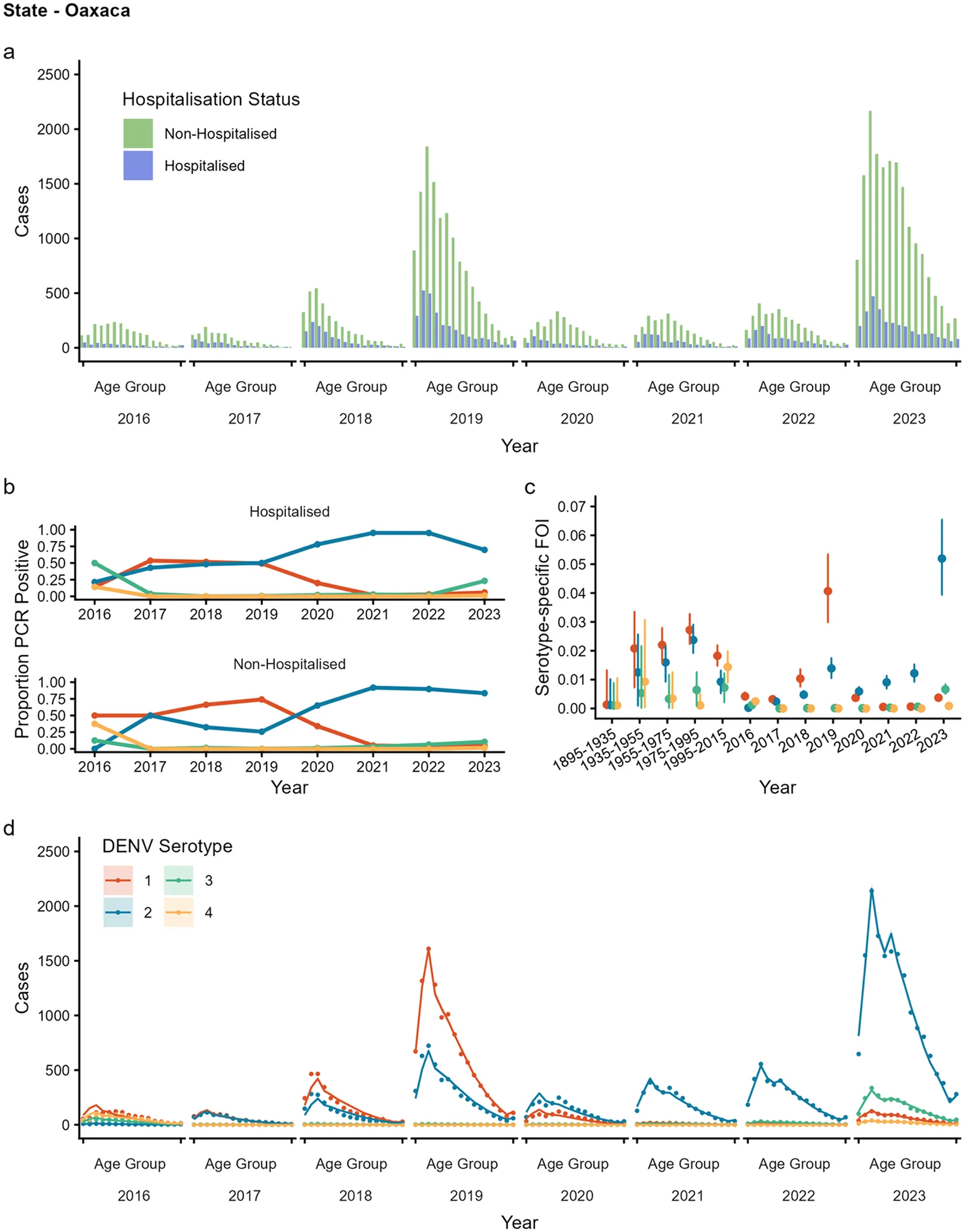
Data and outputs of the serotype-specific model (Model 4) for the state of Oaxaca. **(a)** Number of hospitalised (blue) and non-hospitalised (green) dengue cases per year per five-year age group 0–4, 5–9, …, 70–74, 75+. **(b)** Proportion of positive RT-PCR-test results positive for each DENV serotype each year (DENV-1 red, DENV-2 blue, DENV-3 green and DENV-4 yellow), among hospitalised patients (top) and non-hospitalised patients (bottom). **(c)** Model estimated median serotype-specific FOI and 95% credible intervals for each serotype of DENV modelled (colours the same as in **(b)**) for each year, 2016–2023. **(d)** Model fit (solid lines) to estimated serotype data imputed into the model (dots) by year and by serotype (colours same as before). Imputed data was generated by combining the proportions positive for each serotype among the hospitalised and non-hospitalised patients each year with the case data on annual probable/confirmed hospitalised and non-hospitalised patients.

Fig 6 shows the modelled serotype-specific FOI estimates for Oaxaca, as well as the model’s fit to the estimated serotype-specific incidence, and the data used to estimate this incidence (the equivalent figures for each of the other 24 states are reported in Figs H–AE in S1 Text).

**Fig 7:**
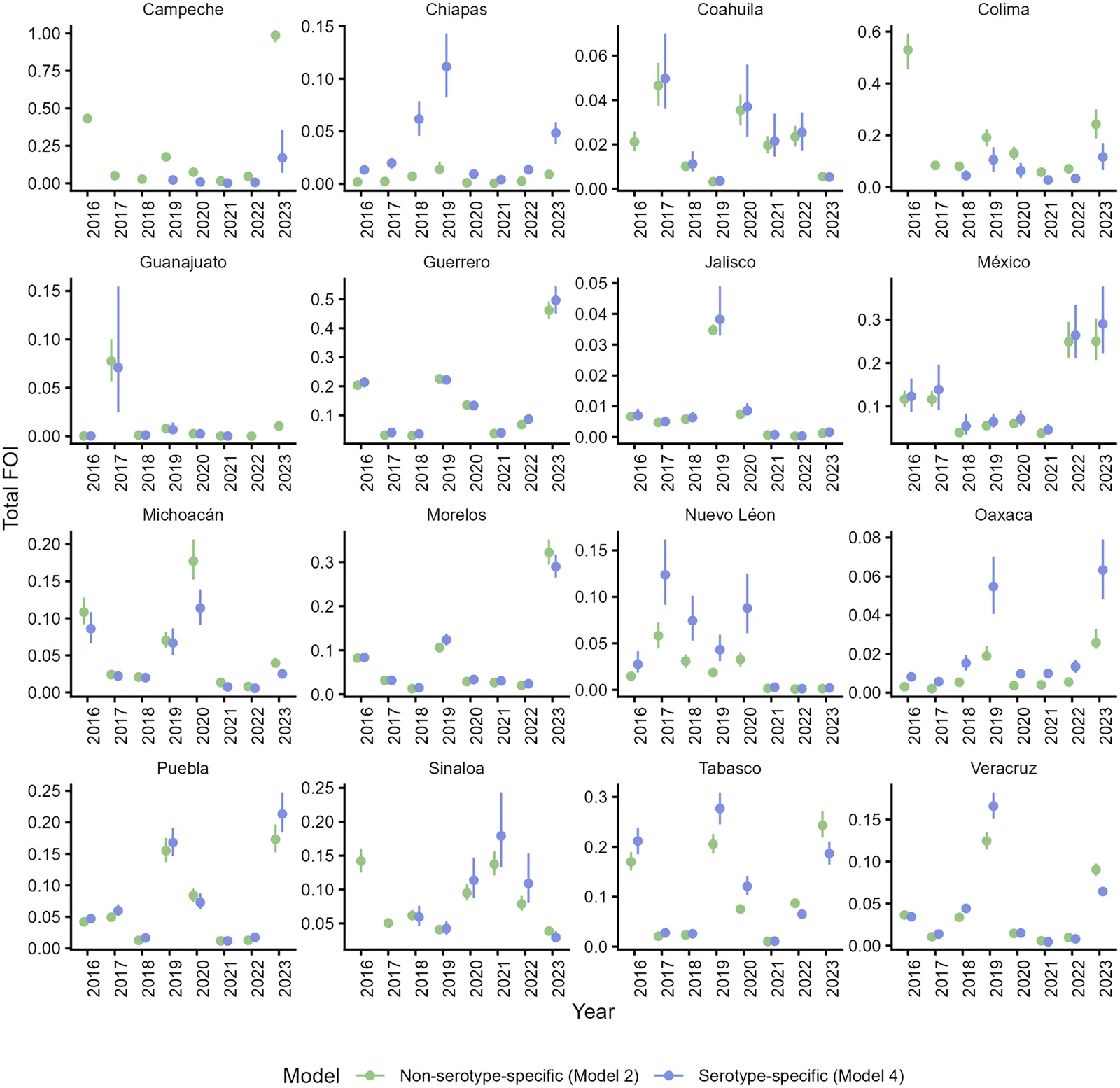
Comparison of model outputs from Models 2 and 4. Median total FOI estimates and 95% credible intervals generated by the non-serotype-specific model (Model 2, green) and the serotype-specific model (Model 4, blue), for states modelled for more than five years in Model 4 (and also for Campeche, which was modelled for five years).

The amount by which the total annual FOI estimates (i.e., the sum of all the serotype-specific estimates for a given year) generated by the serotype-specific model (Model 4) differ from those generated by the non-serotype-specific model (Model 2) varies among the 15 states fitted to more than five years of data, and in Campeche, which was only modelled for five years (Figs 7 and AG in S1 Text). In the majority of these states, the difference between the estimated total annual FOI was small. However, larger differences in the serotype-specific and non-serotype-specific total FOI estimates were observed in Chiapas, Nuevo León and Oaxaca, and states where fewer years of serotype-specific data were available (e.g., Campeche) (see Figs 6 and J, K and V in S1 Text).

## Discussion

This study shows that both overall and serotype-specific DENV FOIs vary substantially in time across Mexico. The serotype-specific model developed in this paper gives new insights on the transmission intensity of DENV serotypes across Mexico, possibly challenging the assumption of equal transmissibility of the four serotypes. We found substantial regional heterogeneity in dengue incidence and DENV transmission intensity in Mexico between 2016 and 2023, and over the 8-year period, DENV-2 was the most frequently detected serotype, followed by DENV-1. DENV-3 frequency was low at the start of the reporting period but substantially increased between 2021 and 2023 in many states in the south-east of the country, and few DENV-4 cases were reported across the whole study period.

In 2023, one of the worst years for dengue in Mexico in the last decade [1], different DENV serotypes caused simultaneous outbreaks in different parts of the country; in the south-east of Mexico, notable outbreaks in Campeche, Tabasco and Quintana Roo were driven by DENV-3, for which we estimated median FOIs of 0.1681 (95% CrI [0.0701, 0.3534]), 0.1556 (95% CrI [0.1358, 0.1771]), and 0.1125 (95% CrI [0.0328, 0.6022]), respectively, and in the centre and west of the country, a resurgence of DENV-1 transmission led to the substantial rise in cases in Puebla (median DENV-1 FOI: 0.1508; 95% CrI [0.1263, 0.1807]) and Guerrero (median DENV-1 FOI: 0.2476; 95% CrI [0.2166, 0.2794]). The impact of climate change on DENV transmission intensity in the modelled period is uncertain, and, in the future, the methods developed in this study could be extended to incorporate climatic factors as drivers of the changes in DENV FOI.

The range of non-serotype-specific total FOI estimates from the constant-in-time non-serotype-specific model (Model 1) — 0.0366 (95% CrI [0.0345, 0.0388]) to 0.0727 (95% CrI [0.0717, 0.0736]) — is similar to, though slightly narrower than, the range of estimates generated for states in Mexico by Rodriguez-Barraquer and colleagues (0.01 to 0.092 on average) [19]. Similarly to O’Driscoll and colleagues [17], we found that the modelled time-varying non-serotype-specific FOIs (i.e., those generated using Model 2) were more variable both within and between states than the constant-in-time non-serotype-specific FOIs generated using Model 1. Both our constant-in-time and time-varying (though non-serotype-specific) FOI estimates tended to be higher in states in the south-east of Mexico, with high DENV FOI estimates in 2023 in states such as Yucatán and Campeche. While our constant-in-time estimate of the FOI in Morelos from Model 1 was similar to the estimate generated by Vicco and colleagues using seroprevalence data from 2011 (0.0473; 95% CrI [0.0465, 0.0481] compared to 0.044; 95% CrI [0.018, 0.087]) [37], our estimate for Yucatán was higher than (but within the credible interval of) Vicco and colleagues’s (which used seroprevalence data from 2014) (0.0727; 95% CrI [0.0717, 0.0736] compared to 0.045; 95% CrI [0.020, 0.081]) [37].

Both the serotype- and non-serotype-specific models ignore cross-protection and may be limited by modelling reporting rates as constant in time and by serotype (implying all serotypes have the same propensity to cause symptomatic disease); increased public awareness during outbreak years may increase reporting rates and previous serotype-specific modelling work suggests that reporting rates may differ between serotypes [38]. The study period also included the COVID-19 pandemic, which may have affected reporting rates during 2020–2022 and could potentially have affected transmission intensity as well, given the restrictions in human mobility; however, the catalytic modelling framework used in this study does not allow us to quantify the mechanisms and extent to which the COVID-19 pandemic affected DENV circulation, which in future studies can be explored utilising mechanistic compartmental models calibrated to time-series data. We assumed that the probability of reporting a DENV infection among children under 15 years old was different than among the rest of the population in all our models with time-varying FOIs, which has been noted in Ecuador [39]. However, the estimated extent to which children reported more or less than adults differed by state, with children under 15 years old estimated to report a high proportion of DENV infections than adults in some states, and a lower proportion in others. More work could be done in the future to better understand age trends in DENV infection reporting rates, both in Mexico and globally.

In this study, we assumed that serotype-specific infection provides long-lived immunity against reinfection, i.e., that homologous reinfection does not occur. Whilst this is a consolidated modelling assumption, reinfections have been reported to occur in some studies [40], and in future work it will be interesting to explore the extent to which modelling homologous reinfections allow us to better capture the observed data. The serotype-specific version of the model, whilst being an improvement over the non-serotype-specific versions, is limited by data availability. Given only 10.3% of cases had a positive RT-PCR result, we reconstructed serotype-specific case data by scaling the total number of hospitalised and non-hospitalised cases by the respective proportions of each serotype observed from RT-PCR testing. As such, these reconstructed data have limitations, as we could not correct for potential differences in serotype-specific severity and therefore propensity to be RT-PCR-tested. Increased availability and use of validated RT-PCR assays capable of reliably distinguishing DENV serotypes in Mexico and across the world would help generate more accurate transmission intensity estimates for the different serotypes.

The surveillance system in Mexico has limitations, as is the case with all passive epidemiological surveillance systems [41]. In particular, there are differences between hospitalised and ambulatory cases, with underreporting higher in the latter group [42]. The database used in this paper is curated and validated annually by the health authorities, however, since transmission intensity changes annually, there may be annual variations in the magnitude of under-reporting. Only 30% of probable outpatient dengue cases are laboratory confirmed during epidemic peaks in Mexico [26], and in the absence of seroprevalence studies it is not possible to generate real-world estimates of the magnitude of this under-reporting. Finally, medical clinics associated with pharmacies (CAP) have expanded in Mexico since 2010; according to the 2023 National Health and Nutrition Survey [43], the number of consultations in this type of primary care clinic could reach 20% of all medical consultations nationwide. In theory, all providers of medical consultations are required to report probable cases of dengue, but the extent to which this regularly occurs remains unclear.

The availability of age-stratified seroprevalence data collected from prospective serological studies or residual samples at a local level (e.g., municipality, city or neighbourhood) would provide real-world evidence of the burden of DENV infection, and coupled with the reported case-notification data, would offer new opportunities to validate the reporting rate estimates generated in this study. Serotype-specific seroprevalence assays would also provide opportunities to validate serotype-specific FOI estimates, though the analysis and interpretation of serotype-specific antibody levels remains challenging due to cross-reactivity [44,45]. In Mexico, to date, population-based seroprevalence surveys have been conducted in Morelos and Yucatán [46,47], and a school-based survey has been conducted across 67 municipalities [48], which underlines the need for additional, systematically designed seroprevalence surveys, conducted in parallel to the surveillance system, to support robust state-level epidemiological inference.

Due to the limitations in serotype-specific data, we assumed that a serotype was not co-circulating in a state if there were fewer than 10 positive RT-PCR tests for that serotype in every modelled year—this assumption could potentially be affected by differing serotype-specific propensities to cause severe disease, hospitalisation and RT-PCR testing. We also assumed that if a serotype was not circulating between 2016 and 2023, it was also not circulating prior to 2016, given the catalytic modelling framework does not allow us to reconstruct whether high levels of population immunity or lack of introduction drive the absence of circulation of a serotype. Going forward, it would be useful to investigate and disentangle the potential mechanisms behind heterogeneities in serotype circulation, particularly the role that prior immunity plays in the low incidence and modelled transmission intensity of DENV-4, which has the lowest FOI of all four serotypes in the eight states in which we modelled the transmission of this serotype, which is not consistent with previous serotype-specific FOI estimates obtained in Peru and French Polynesia [22,38].

The development of mathematical models to estimate the serotype-specific transmission intensity of DENV when serotypes are co-circulating from routine case data is a novel contribution of this paper which should stimulate new research and evidence generation from multiple settings. Better characterising serotype-specific variations in transmissibility will allow the generation of refined global DENV transmission intensity and burden maps (such as https://arbomap.org), which currently assume equal transmission intensity of the four serotypes. Most importantly, given all currently available (and upcoming) dengue vaccines have complex and serotype-specific efficacy profiles, it is crucial to assess the extent to which the different serotypes may differ in their transmission potentials and in their propensities to generate severe disease, as these estimates will inform vaccine impact modelling studies, global guidelines and national vaccination programmes going forward.

## Supporting information

S1 Text Supplementary Information (Methods, Figures and Tables). (DOCX)

S1 Checklist RECORD checklist. Benchimol EI, Smeeth L, Guttmann A, Harron K, Moher D, Petersen I, Sørensen HT, von Elm E, Langan SM, the RECORD Working Committee. The REporting of studies Conducted using Observational Routinely-collected health Data (RECORD) Statement. *PLoS Medicine* 2015; in press. Checklist is protected under Creative Commons Attribution (CC BY) license.
(DOCX)

## Abbreviations

CAP: clinics associated with pharmacies
CFR: case fatality rate
DENV: dengue virus
DIC: deviance information criterion
FOI: force of infection
INAI: Instituto Nacional de Acceso a la Información
LOOIC: leave-one-out information criterion
PAHO: Pan American Health Organization
RECORD: Reporting of Studies Conducted Using Observational Routinely-Collected Data
RT-PCR: reverse transcription polymerase chain reaction
SINAVE: Sistema Nacional de Vigilancia Epidemiológica
WAIC: widely-applicable information criterion
