# Supplementary material for: Overall and serotype-specific dengue virus transmission intensity in Mexico, 2016–2023: A modelling study of case-notification data
Source: PLoS Med. 2026 Sep 3;23(9):e1004874. doi: 10.1371/journal.pmed.1004874 (PMC13541250; doi:10.1371/journal.pmed.1004874)
Supplement: Supplementary file 1 [file pmed.1004874.s004.pdf]

This paper provides various models for the transmission of dengue virus (DENV) in México.

I was asked for a methodological report and I interpret that to include all aspects of the design and conduct of the study. The version I received did not have page numbers. My numbering has the abstract starting on page 1

Points of detail

Page 1 The title seems rather terse. As I understand it much of the novelty here is the investigation of the four serotypes of DENV but the casual reader of the title would not pick that up.

Page 4 Given the small number of people with missing ages it probably does not matter what was imputed for them but the strategy here does seem rather crude. I would have been inclined just to remove them.

Page 5 If the serotypes lead to disease of different severity then the assumption that serotype prevalancce is the same for hospitalised cases and non–hospitalised seems dubious. Do we know that the disease severity is the same?

Page 6 My ageing eyes found it hard to read some of the formulas and equation 2 is a good example. A thin space between the terms like $p_2 \sum_{a \in \tilde{A}_j} I_{2,a,t}$ would help to see where one ends and the next one begins

Page 7 I do not understand the significance of \ in some of the subscripts. Is this the set complement operator?

Page 8 It might be better to move the justification for omitting those below one year to before the mention of the omission on page 6.

Page 9 The results of the convergence test do not seem to be reported in the results. A simple sentence confirming they converged is enough.

Page 9 Naming the packages used is a good idea but if any of them is crucial to the modelling rather than data manipulation a full citation would be better.

Page 10 I can understand why on page 2 we have confidence intervals as those are presumably quoted from other sources but when we start to see the results why do we start with confidence intervals here before moving on to credible intervals on page 14? Is this just a typo or is there some reason?

Page 12 Is it possible to remove the distracting international boundaries in Central America? Removing the US as well would do no harm.

Page 12 The caption could explain why there are grey areas in sub–panels c and d but not a and b.

Page 14 Figure 2 presents the states in alphabetical order of their names. Would it emphasise the results if they were grouped according to the pattern of the serotypes over time? The caption might point out the México here is not the country as a whole nor Ciudad de México but a state named México. At least I assume that is the case. Not all readers will be as familiar with local geography as the authors.

Page 18 I did not find this very informative. Because some states had very extreme values of FOI the remainder are very compressed and hard to distinguish. The state names in sub–panel a do not appear well aligned with the data points.

Page 20, Figure 7 Usually to compare two quantities one plots one against the other or some function(s) of the two quantities. The current presentation rather obscures the fact that Michoacán is rather different from the four mentioned in the text since they all have the discrepant points in the opposite order. I suggest plotting the FOIs against one another with the credible intervals as cross–hairs and code year in some way perhaps using colours on a continuum.

Page 21 and elsewhere If the authors wish to compare with previous work it would be good to make clear which estimates are which. If in fact the estimates given are only those from the present work then please quote the previous ones to save readers having to open several articles to see what they were.

Page 23 The database limitations to which the authors allude here seem to me to be hard to avoid when using routine data. That should not stop us trying of course.

As a very minor point the authors are not consistent about place names. We have the state names using the Spanish orthography everywhere. For example Yucatán not Yucatan but Mexico and Peru in some places. Unless PLOS has a policy on this I would suggest keeping to one approach. Personally I would prefer the Spanish orthography. At least in the UK there has been a gradual move to using local orthography over the last decades. I would remove *Entidad* from the caption in Figure 5 though.

Looking at the relevant publication guideline which I believe to be RECORD

(Benchimol et al., 2015) I cannot see major omission but I think recognition of those guidelines would be helpful.

Concluding remarks

The main message I draw from reading this is that there is massive heterogeneity. The authors have done a great deal of work to establish the facts but I did not find much explanation of possible moderator variables here. I am not though an expert in virology and have very limited knowledge of the physical or human geography of México.

Michael Dewey

References

E I Benchimol, L Smeeth, A Guttmann, K Harron, D Moher, I Petersen, H Sørensen, E von Elm, S Langan, and RECORD working committee. The REporting of studies Conducted using Observational Routinely–collected health Data(RECORD) Statement. *PLOS Medicine*, 12(10), 2015.
