# Supplementary material for: Overall and serotype-specific dengue virus transmission intensity in Mexico, 2016–2023: A modelling study of case-notification data
Source: PLoS Med. 2026 Sep 3;23(9):e1004874. doi: 10.1371/journal.pmed.1004874 (PMC13541250; doi:10.1371/journal.pmed.1004874)
Supplement: Supplementary file 2 [file pmed.1004874.s001.docx]

# Supplementary Information

# Overall and serotype-specific dengue virus transmission intensity in Mexico, 2016-2023: a modelling study of case-notification data

Table of Contents

[Supplementary Methods	2](#_Toc224562655)

[Equations for Model 2	2](#_Toc224562656)

[Supplementary Figures	3](#_Toc224562657)

[Supplementary Tables	35](#_Toc224562658)

[References	37](#_Toc224562659)

## Supplementary Methods

### Equations for Model 2

$$\begin{aligned} S\left( a,0 \right)=\exp\left( -4f(a) \right)\#\left( S1 \right) \end{aligned}$$

$$\begin{aligned} M\left( a,0 \right)=4\cdot exp \left( -3f(a) \right)\left( 1-\exp\left( -f\left( a \right) \right) \right) \#\left( S2 \right) \end{aligned}$$

where

$$\begin{aligned} f\left( a \right)= \sum_{k = 1}^{5} \lambda_{H_{k}}\mathbb{1}_{\{a > 20\left( k-1 \right)\}}\min(a-20(k-1), 20)\#\left( S3 \right) \end{aligned}\begin{aligned} l\left( a, \omega\right)= \sigma_{H_{k}}\lambda_{H_{k}}^{\omega}¨¨\mathbb{1}_{x} \#\left( 11 \right) \end{aligned}$$

For $t\in T,a\neq0$,

$$\begin{aligned} S\left( a,t \right)=S\left( a-1,t-1 \right)-4\lambda_{t}\cdot S\left( a-1,t-1 \right)\#\left( S4 \right) \end{aligned}$$

$$\begin{aligned} M\left( a,t \right)=M\left( a-1,t-1 \right)+4\lambda_{t}\cdot S\left( a-1,t-1 \right)-3\lambda_{t}\cdot M\left( a-1,t-1 \right) \#\left( S5 \right) \end{aligned}$$

$$\begin{aligned} I_{1}\left( a,t \right)=4\lambda_{t}\cdot S\left( a,t-1 \right)\#\left( S6 \right) \end{aligned}$$

$$\begin{aligned} I_{2}\left( a,t \right)=3\lambda_{t}\cdot M\left( a,t-1 \right)\#\left( S7 \right) \end{aligned}$$

This is a similar model structure to that developed by O’Driscoll et al. 2020 [1], except the historic FOI is allowed to vary. Here $S(a,t)$ denotes the proportion of the population of age $a$ susceptible at the end of year $t$, $M\left( a,t \right)$ the proportion of the population monotypically infected with DENV at age $a$ at the end of year $t$, $I_{1}\left( a,t \right)$ the annual primary incidence of DENV in age $a$ in year $t$, and $I_{2}\left( a,t \right)$ the annual secondary incidence of DENV in age $a$ in year $t$.

## Supplementary Figures

Fig A: Map of federal entities (states) in Mexico. CDMX refers to Ciudad de México. The shapefiles used to generate the maps in the figure above were obtained from GADM (https://gadm.org/) and can be accessed by going to <https://gadm.org/download_country.html> and selecting “Mexico” from the drop-down menu.

Fig B: Proportion of RT-PCR-positive patients positive for each DENV serotype among hospitalised patients (a) and non-hospitalised patients (b) between 2016 and 2023 by state and age group.

Fig C: Number of RT-PCR-positive patients positive for each DENV serotype among hospitalised patients (a) and non-hospitalised patients (b) between 2016 and 2023 by state and age group.

Fig D: Proportion of probable and confirmed dengue cases hospitalised by state 2016-2023 (a) and dengue probable/confirmed case fatality ratio (CFR) by state 2016-2023 (b). The shapefiles used to generate the maps in the figure above were obtained from GADM (https://gadm.org/) and can be accessed as described above.

Fig E: Yearly dengue incidence by state, 2016-2023. The shapefiles used to generate the maps in the figure above were obtained from GADM (https://gadm.org/) and can be accessed as described above.

Fig F: Median secondary reporting rates ($\rho$) (a.), primary reporting rates ($\gamma$) (b.) and reporting multiplier for children under 15 ($\chi$) (b.) by state, generated by Model 2 (time-varying non-serotype-specific model with variable historic FOI). The shapefiles used to generate the maps in the figure above were obtained from GADM (https://gadm.org/) and can be accessed as described above.

Fig G: Median secondary reporting rates ($\rho$) (a.), primary reporting rates ($\gamma$) (b.) and reporting multiplier for children under 15 ($\chi$) (c.) by state, generated by the serotype-specific model (Model 4). The shapefiles used to generate the maps in the figure above were obtained from GADM (https://gadm.org/) and can be accessed as described above.

Fig H: Data used for and outputs of the serotype-specific model (Model 4) for the state of Aguascalientes. a: Number of hospitalised (blue) and non-hospitalised (green) dengue cases per year per five-year age group 0-4, 5-9, …, 70-74, 75+. b: Proportion of positive RT-PCR-test results positive for each serotype of dengue each year (DENV1 red, DENV2 blue, DENV-3 green and DENV-4 yellow), among hospitalised patients (top) and non-hospitalised patients (bottom). c: Model estimated median serotype-specific FOI and 95% credible intervals for each serotype of DENV modelled (colouring the same as in plot b) for each year, 2016-2023. d: Model fit (solid lines) to estimated serotype data imputed into the model (dots) by year and by serotype (colours same as before). Imputed data was generated by combining the proportions positive for each serotype among the hospitalised and non-hospitalised patients each year with the case data on annual probable/confirmed hospitalised and non-hospitalised patients. DENV-4 was assumed not to be in circulation in this state (see Methods for details).

Fig I: Data used for and outputs of the serotype-specific model (Model 4) for the state of Baja California Sur. DENV-4 was assumed not to be in circulation (see Methods for details).

Fig J: Data used for and outputs of the serotype-specific model (Model 4) for the state of Campeche. DENV-4 was assumed not to be in circulation (see Methods for details).

Fig K: Data used for and outputs of the serotype-specific model (Model 4) for the state of Chiapas.

Fig L: Data used for and outputs of the serotype-specific model (Model 4) for the state of Coahuila. DENV-4 was assumed not to be in circulation (see Methods for details).

Fig M: Data used for and outputs of the serotype-specific model (Model 4) for the state of Colima. DENV-4 was assumed not to be in circulation (see Methods for details).

Fig N: Data used for and outputs of the serotype-specific model (Model 4) for the state of Guanajuato. DENV-3 and DENV-4 were assumed not to be in circulation (see Methods for details).

Fig O: Data used for and outputs of the serotype-specific model (Model 4) for the state of Guerrero.

Fig P: Data used for and outputs of the serotype-specific model (Model 4) for the state of Hidalgo. DENV-4 was assumed not to be in circulation (see Methods for details).

Fig Q: Data used for and outputs of the serotype-specific model (Model 4) for the state of Jalisco. DENV-4 was assumed not to be in circulation (see Methods for details).

Fig R: Data used for and outputs of the serotype-specific model (Model 4) for the state of México. Note: DENV-4 assumed not to be in circulation, therefore not contributing to the dengue FOI.

Fig S: Data used for and outputs of the serotype-specific model (Model 4) for the state of Michoacán. Note: DENV-4 assumed not to be in circulation, therefore not contributing to the dengue FOI.

Fig T: Data used for and outputs of the serotype-specific model (Model 4) for the state of Morelos. Note: DENV-4 assumed not to be in circulation, therefore not contributing to the dengue FOI.

Fig U: Data used for and outputs of the serotype-specific model (Model 4) for the state of Nayarit. DENV-3 and DENV-4 were assumed not to be in circulation (see Methods for details).

Fig V: Data used for and outputs of the serotype-specific model (Model 4) for the state of Nuevo León. DENV-4 was assumed not to be in circulation (see Methods for details).

Fig W: Data used for and outputs of the serotype-specific model (Model 4) for the state of Puebla.

Fig X: Data used for and outputs of the serotype-specific model (Model 4) for the state of Querétaro. DENV-4 was assumed not to be in circulation (see Methods for details).

Fig Y: Data used for and outputs of the serotype-specific model (Model 4) for the state of Quintana Roo.

Fig Z: Data used for and outputs of the serotype-specific model (Model 4) for the state of San Luis Potosí. DENV-3 and DENV-4 were assumed not to be in circulation (see Methods for details).

Fig AA: Data used for and outputs of the serotype-specific model (Model 4) for the state of Sinaloa. DENV-4 was assumed not to be in circulation (see Methods for details).

Fig AB: Data used for and outputs of the serotype-specific model (Model 4) for the state of Sonora. DENV-4 was assumed not to be in circulation (see Methods for details).

Fig AC: Data used for and outputs of the serotype-specific model (Model 4) for the state of Tabasco.

Fig AD: Data used for and outputs of the serotype-specific model (Model 4) for the state of Tamaulipas. DENV-4 was assumed not to be in circulation (see Methods for details).

Fig AE: Data used for and outputs of the serotype-specific model (Model 4) for the state of Veracruz.

Fig AF: Model 2 (best non-serotype-specific model) outputs of $\lambda_{t}$ by state and year. Here "1995-2015", “1975-1995”, “1955-1975”, “1935-1955” and “1895-1935” are the five historical FOI used to generate the initial immunity profile of the population (referred to as $\lambda_{H_{1}}$, $\lambda_{H_{2}}$, $\lambda_{H_{3}}$, $\lambda_{H_{4}}$, and $\lambda_{H_{5}}$respectively in the equations above). Points are the median estimates, and the error bars represent the 95% credible interval of results.

Fig AG: Comparison between total force of infection estimates between the best performing non-serotype-specific model (Model 2) and the serotype-specific model (Model 4), for each modelled year for states fitted to more than 5 years of data (and Campeche, which was fitted to 5 years of data). Points show the median total FOI estimate, and error bars 95% credible intervals. Total force of infection estimates were calculated either by summing the FOI estimates of all serotypes (in the case of the serotype-specific model) or by multiplying the per-serotype FOI estimate by four (in the case of the non-serotype-specific model).

## Supplementary Tables

| Parameter | Description | Model | Prior | Support Constraints |
| --- | --- | --- | --- | --- |
| $\lambda$ | Time-constant per-serotype FOI. | Model 1 | *Normal(0,1)* | $[0,0.25]$ |
| $\lambda_{t}$  $t\in\left\{ 2016, 2017, \ldots, 2023 \right\}$ | Per-serotype FOI during year $t$. | Model 2,3 | *Normal(0,1)* | $[0,0.25]$ |
| $\lambda_{t}^{\omega}$  $t\in\{2016, 2017, \ldots, 2023\},$  $\omega\in\{1,2,3,4\}$ | Proportion of the total FOI attributable to serotype $\omega$ during year $t$. | Model 4 | *Dir(1,1,1,1)* | $[0,1]$ |
| $\sigma_{t}$  $t\in\{2016, 2017, \ldots, 2023\}$ | Total FOI of all four serotypes during year $t$. | Model 4 | *Uniform(0,1)* | $[0,1]$ |
| $\lambda_{H}$ | Time-constant historic per-serotype FOI. | Model 3 | *Normal(0,1)* | $[0,0.25]$ |
| $\lambda_{H_{k}}$  $k\in\{1,2,3,4,5\}$ | Time-varying historic per-serotype FOI during time period $k$. | Model 2 | *Normal(0,1)* | $[0,0.25]$ |
| $\lambda_{H_{k}}^{\omega}$  $k\in\left\{ 1,2,3,4,5 \right\},$  $\omega\in\{1,2,3,4\}$ | Proportion of the total time-varying historic FOI attributable to serotype $\omega$ during time period $k$. | Model 4 | *Dir(1,1,1,1)* | $[0,1]$ |
| $\sigma_{H_{k}}$  $k\in\left\{ 1,2,3,4,5 \right\}$ | Total time-varying historic FOI of all four serotypes during time period $k$. | Model 4 | *Uniform(0,1)* | $[0,1]$ |
| $\rho$ | Secondary reporting rate. | Model 1,2,3,4 | *Normal(0,1)* | $[0,1]$ |
| $\gamma$ | Primary reporting rate. | Model 2,3,4 | *Normal(0,1)* | $[0,1]$ |
| $\tilde{\gamma}$ | Primary reporting rate relative to secondary reporting rate. | Model 1 | *Normal(0,1)* | $[0,1]$ |
| $\tilde{\chi_{J}}$  $J\in\{1,2,3\}$  $(\tilde{\chi_{J}}=1 \forall J\in\left\{ 4,5,6,\ldots,16 \right\})$ | Paediatric reporting modifier. | Model 2,3,4 | *Normal(1,1)* | ℝ_>0_, but also such that $\tilde{\chi_{J}}\cdot\rho\leq1$ and $\tilde{\chi_{J}}\cdot\gamma\leq1$ |

Table A: Description of model parameters used in Models 1-4.

| State | Median Age of Reported Dengue Cases - Overall | Median Age of Reported Dengue Cases - Females | Median Age of Reported Dengue Cases - Males |
| --- | --- | --- | --- |
| Aguascalientes | 29 (18, 42) | 31 (20, 46.5) | 26 (16.2, 38) |
| Baja California | 30.5 (17, 46) | 33 (21, 46) | 28 (14, 44.5) |
| Baja California Sur | 28 (15, 42) | 29 (17, 43) | 27 (14, 41) |
| Campeche | 23 (12, 36) | 24 (13, 38) | 21 (12, 35) |
| Ciudad de México | 35 (27, 50) | 36.5 (27, 52) | 33 (26, 48) |
| Chiapas | 14 (7, 28) | 15 (7, 30) | 13 (7, 26) |
| Chihuahua | 33 (20, 46) | 34.5 (22, 48) | 30 (16, 43) |
| Coahuila | 31 (21, 44) | 33 (22, 45) | 29 (19, 42) |
| Colima | 22 (11, 36) | 24 (12, 38) | 20 (10, 34) |
| Durango | 31 (19, 44) | 33 (21, 45) | 29 (18, 43) |
| Guanajuato | 29 (18, 42) | 30 (19, 44) | 26 (16, 39) |
| Guerrero | 19 (11, 35) | 21 (11, 36) | 17 (10, 33) |
| Hidalgo | 28 (15, 42) | 28 (17, 42) | 26 (14, 42) |
| Jalisco | 29 (17, 44) | 31 (19, 46) | 27 (16, 42) |
| México | 27 (15, 42) | 29 (17, 44) | 25 (13, 41) |
| Michoacán | 28 (15, 41) | 29 (18, 43) | 25 (14, 39) |
| Morelos | 28 (15, 42) | 29 (17, 44) | 26 (14, 40) |
| Nayarit | 28 (15, 42) | 30 (17, 43) | 26 (14, 41) |
| Nuevo León | 29 (18, 44) | 32 (20, 46) | 27 (16, 41) |
| Oaxaca | 24 (12, 39) | 26 (13, 40) | 21 (12, 36) |
| Puebla | 26 (14, 40) | 28 (16, 41) | 23 (13, 37) |
| Querétaro | 29 (19, 42) | 31 (21, 45) | 27 (15, 39) |
| Quintana Roo | 25 (14, 36) | 26 (15, 37) | 24 (13, 35) |
| San Luis Potosí | 26 (15, 39) | 28 (17, 40) | 23 (13, 37) |
| Sinaloa | 27 (15, 42) | 29 (17, 43) | 24 (14, 40) |
| Sonora | 28 (17, 44) | 30 (18, 45) | 27 (16, 43) |
| Tabasco | 24 (13, 37) | 26 (14, 39) | 22 (12, 35) |
| Tamaulipas | 27 (16, 41) | 29 (18, 43) | 24 (14, 38) |
| Tlaxcala | 34 (25, 49) | 38 (29, 52) | 30 (21.2, 43.8) |
| Veracruz | 27 (14, 41) | 28 (15, 43) | 24 (13, 39) |
| Yucatán | 21 (12, 33) | 22 (13, 35) | 20 (12, 32) |
| Zacatecas | 31 (18.2, 44.8) | 32 (21, 45) | 29 (16, 44) |

Table B: Median age of probable/confirmed reported cases of dengue by state during the period 2016-2023. 25% and 75% quantiles are provided in brackets.

| **State** | **Model 2 DIC** | **Model 3 DIC** | **Model 2 WAIC** | **Model 3 WAIC** | **Model 2 LOOIC** | **Model 3 LOOIC** | **Best by DIC** | **Best by WAIC** | **Best by LOOIC** |
| --- | --- | --- | --- | --- | --- | --- | --- | --- | --- |
| Aguascalientes | 260.6491 | 281.7209 | 287.935 | 285.3002 | 298.044 | 296.4367 | Model 2 | Model 3 | Model 3 |
| Baja California Sur | 856.3105 | 882.5461 | 916.1501 | 915.7019 | 918.6705 | 921.1093 | Model 2 | Model 3 | Model 2 |
| Campeche | 1196.444 | 1367.656 | 1365.006 | 1472.604 | 1363.215 | 1451.892 | Model 2 | Model 2 | Model 2 |
| Chiapas | 2476.729 | 2606.683 | 2766.931 | 3310.852 | 2666.264 | 3193.625 | Model 2 | Model 2 | Model 2 |
| Coahuila | 1004.494 | 1126.578 | 1056.022 | 1143.931 | 1062.782 | 1149.234 | Model 2 | Model 2 | Model 2 |
| Colima | 1141.317 | 1225.31 | 1210.448 | 1249.41 | 1215.631 | 1254.797 | Model 2 | Model 2 | Model 2 |
| Durango | 375.2304 | 634.2028 | 628.001 | 656.2271 | 636.5939 | 664.4009 | Model 2 | Model 2 | Model 2 |
| Guanajuato | 808.6494 | 950.5674 | 952.9577 | 975.502 | 960.4193 | 983.8781 | Model 2 | Model 2 | Model 2 |
| Guerrero | 1910.389 | 2222.973 | 2197.498 | 2362.506 | 2159.086 | 2336.901 | Model 2 | Model 2 | Model 2 |
| Hidalgo | 795.8433 | 854.7552 | 875.1979 | 902.6863 | 881.3909 | 907.4272 | Model 2 | Model 2 | Model 2 |
| Jalisco | 2660.358 | 2788.724 | 2994.501 | 3077.82 | 2815.186 | 2928.65 | Model 2 | Model 2 | Model 2 |
| México | 800.2135 | 1054.329 | 1014.965 | 1069.774 | 1022.631 | 1077.213 | Model 2 | Model 2 | Model 2 |
| Michoacán | 1480.237 | 1644.089 | 1688.857 | 1838.284 | 1589.599 | 1758.149 | Model 2 | Model 2 | Model 2 |
| Morelos | 1407.117 | 1512.37 | 1456.527 | 1540.58 | 1458.246 | 1549.258 | Model 2 | Model 2 | Model 2 |
| Nayarit | 1203.283 | 1297.197 | 1297.75 | 1388.094 | 1290.108 | 1386.286 | Model 2 | Model 2 | Model 2 |
| Nuevo León | 1691.744 | 1707.301 | 1863.82 | 1867.679 | 1800.866 | 1823.174 | Model 2 | Model 2 | Model 2 |
| Oaxaca | 2085.506 | 2362.66 | 2380.296 | 2512.446 | 2348.895 | 2488.994 | Model 2 | Model 2 | Model 2 |
| Puebla | 1134.467 | 1492.752 | 1602.114 | 1623.752 | 1568.455 | 1591.918 | Model 2 | Model 2 | Model 2 |
| Querétaro | 490.757 | 494.3353 | 514.6559 | 509.4043 | 520.1608 | 516.1364 | Model 2 | Model 3 | Model 3 |
| Quintana Roo | 1402.726 | 1943.63 | 1881.434 | 2260.615 | 1801.692 | 2100.107 | Model 2 | Model 2 | Model 2 |
| San Luis Potosí | 635.0459 | 1292.241 | 1216.114 | 1386.056 | 1196.284 | 1379.722 | Model 2 | Model 2 | Model 2 |
| Sinaloa | 1266.051 | 1324.076 | 1305.189 | 1349.506 | 1310.955 | 1353.768 | Model 2 | Model 2 | Model 2 |
| Sonora | 775.2872 | 930.549 | 884.7443 | 932.4567 | 891.767 | 939.418 | Model 2 | Model 2 | Model 2 |
| Tabasco | 859.8486 | 1478.739 | 1426.207 | 1634.914 | 1415.32 | 1595.174 | Model 2 | Model 2 | Model 2 |
| Tamaulipas | 1576.004 | 1618.379 | 1718.851 | 1716.977 | 1697.066 | 1705.146 | Model 2 | Model 3 | Model 2 |
| Veracruz | 675.1925 | 3389.266 | 4073.936 | 4181.93 | 3374.742 | 3672.705 | Model 2 | Model 2 | Model 2 |
| Yucatán | 2113.179 | 2706.852 | 2331.63 | 2952.37 | 2238.132 | 2866.286 | Model 2 | Model 2 | Model 2 |

Table C: Comparison of DIC, WAIC, and LOOIC between the two versions of the non-serotype-specific model (Models 2-3). Model 2 allows the historic FOI prior to 2016 to vary whereas Model 3 uses a constant historic FOI.

## References

1.	O'Driscoll M, Imai N, Ferguson NM, Hadinegoro SR, Satari HI, Tam CC, et al. Spatiotemporal variability in dengue transmission intensity in Jakarta, Indonesia. PLoS Negl Trop Dis. 2020;14(3):e0008102. Epub 20200306. doi: 10.1371/journal.pntd.0008102. PubMed PMID: 32142516; PubMed Central PMCID: PMCPMC7080271.
