# Supplementary material for: Overall and serotype-specific dengue virus transmission intensity in Mexico, 2016–2023: A modelling study of case-notification data
Source: PLoS Med. 2026 Sep 3;23(9):e1004874. doi: 10.1371/journal.pmed.1004874 (PMC13541250; doi:10.1371/journal.pmed.1004874)
Supplement: Supplementary file 3 [file pmed.1004874.s002.docx]

**The RECORD statement – checklist of items, extended from the STROBE statement, that should be reported in observational studies using routinely collected health data.**

|  | **Item No.** | **STROBE items** | **Location in manuscript where items are reported** | **RECORD items** | **Location in manuscript where items are reported** |
| --- | --- | --- | --- | --- | --- |
| **Title and abstract** | | | | | |
|  | 1 | (a) Indicate the study’s design with a commonly used term in the title or the abstract (b) Provide in the abstract an informative and balanced summary of what was done and what was found | See Title & Abstract, both pp1-2. | RECORD 1.1: The type of data used should be specified in the title or abstract. When possible, the name of the databases used should be included.  RECORD 1.2: If applicable, the geographic region and timeframe within which the study took place should be reported in the title or abstract.  RECORD 1.3: If linkage between databases was conducted for the study, this should be clearly stated in the title or abstract. | See Title & Abstract, both pp1-2. |
| **Introduction** | | | | | |
| Background rationale | 2 | Explain the scientific background and rationale for the investigation being reported | Introduction, pp3-6. |  |  |
| Objectives | 3 | State specific objectives, including any prespecified hypotheses | Introduction, pp5-6. |  |  |
| **Methods** | | | | | |
| Study Design | 4 | Present key elements of study design early in the paper | Methods, Data, Epidemiological Data, pp6-7. |  |  |
| Setting | 5 | Describe the setting, locations, and relevant dates, including periods of recruitment, exposure, follow-up, and data collection | Methods, Data, Epidemiological Data, pp6-7. |  |  |
| Participants | 6 | *(a) Cohort study* - Give the eligibility criteria, and the sources and methods of selection of participants. Describe methods of follow-up  *Case-control study* - Give the eligibility criteria, and the sources and methods of case ascertainment and control selection. Give the rationale for the choice of cases and controls  *Cross-sectional study* - Give the eligibility criteria, and the sources and methods of selection of participants  *(b) Cohort study* - For matched studies, give matching criteria and number of exposed and unexposed  *Case-control study* - For matched studies, give matching criteria and the number of controls per case | Methods, Data, Epidemiological Data, pp6-7. | RECORD 6.1: The methods of study population selection (such as codes or algorithms used to identify subjects) should be listed in detail. If this is not possible, an explanation should be provided.  RECORD 6.2: Any validation studies of the codes or algorithms used to select the population should be referenced. If validation was conducted for this study and not published elsewhere, detailed methods and results should be provided.  RECORD 6.3: If the study involved linkage of databases, consider use of a flow diagram or other graphical display to demonstrate the data linkage process, including the number of individuals with linked data at each stage. | 6.1 – See Methods, Data, Epidemiological Data, pp6-7. Study population selected is all probable or confirmed cases with residence in Mexico, definitions provided or given in reference due to length.  6.2 – NA, no validation done.  6.3 – No data linkage on the individual level. Multiple datasets used a data split annually (for 2020-2023) or combined over 4 years (2016-2019). |
| Variables | 7 | Clearly define all outcomes, exposures, predictors, potential confounders, and effect modifiers. Give diagnostic criteria, if applicable. | English translations of the variables and outcomes used have been provided, with the original Spanish given in brackets after (Methods, Data, Epidemiological Data, pp6-7). Definitions have been given where applicable, except for probable cases, where, due to length, the reference where this is defined has been provided instead. | RECORD 7.1: A complete list of codes and algorithms used to classify exposures, outcomes, confounders, and effect modifiers should be provided. If these cannot be reported, an explanation should be provided. | See previous answer. |
| Data sources/ measurement | 8 | For each variable of interest, give sources of data and details of methods of assessment (measurement).  Describe comparability of assessment methods if there is more than one group | For data sources and details of methods of assessment see Methods, Data, Epidemiological Data, pp6-7. |  |  |
| Bias | 9 | Describe any efforts to address potential sources of bias | For model data imputation, see Methods, Serotype-specific case incidence, pp8-9. |  |  |
| Study size | 10 | Explain how the study size was arrived at | All confirmed or probable cases, see Methods, Data, Population Data, pp7-8. |  |  |
| Quantitative variables | 11 | Explain how quantitative variables were handled in the analyses. If applicable, describe which groupings were chosen, and why | For age groupings of population, see Methods, Data, Population Data, pp7-8, and Methods, Non-serotype-specific Models, p9. |  |  |
| Statistical methods | 12 | (a) Describe all statistical methods, including those used to control for confounding  (b) Describe any methods used to examine subgroups and interactions  (c) Explain how missing data were addressed  (d) *Cohort study* - If applicable, explain how loss to follow-up was addressed  *Case-control study* - If applicable, explain how matching of cases and controls was addressed  *Cross-sectional study* - If applicable, describe analytical methods taking account of sampling strategy  (e) Describe any sensitivity analyses | (a&b) Statistical tests for comparisons between population groups and methods for generating confidence intervals are detailed in Methods, Data, Epidemiological Data (p7) and where used in Results, Descriptive Analysis (pp13-15). Catalytic modelling used to estimate transmission intensity, fit to data using MCMC methods, see Methods, Mathematical Models, pp 9-12.  (c) Missing data on hospitalisation status is covered in Methods, Data, Serotype-specific case incidence, p8) and missing data on population sizes from the 2015 intercensal survey and 2020 census in Methods, Data, Population Data, pp7-8. |  |  |
| Data access and cleaning methods |  | .. |  | RECORD 12.1: Authors should describe the extent to which the investigators had access to the database population used to create the study population.  RECORD 12.2: Authors should provide information on the data cleaning methods used in the study. | Methods, Data, Epidemiological Data, pp 6-7 and Data Availability, p13. |
| Linkage |  | .. |  | RECORD 12.3: State whether the study included person-level, institutional-level, or other data linkage across two or more databases. The methods of linkage and methods of linkage quality evaluation should be provided. | NA |
| **Results** | | | | | |
| Participants | 13 | (a) Report the numbers of individuals at each stage of the study (*e.g.*, numbers potentially eligible, examined for eligibility, confirmed eligible, included in the study, completing follow-up, and analysed)  (b) Give reasons for non-participation at each stage.  (c) Consider use of a flow diagram | (a) Total number of cases and total number of probable and confirmed cases are detailed in Methods, Data, Epidemiological Data, p6. The total number of people analysed is also given on p6, and repeated at the start of Results, Descriptive Analysis (p13).  (b&c) - NA | RECORD 13.1: Describe in detail the selection of the persons included in the study (*i.e.,* study population selection) including filtering based on data quality, data availability and linkage. The selection of included persons can be described in the text and/or by means of the study flow diagram. | Persons selected detailed in Methods, Data, Epidemiological Data, pp6-7. |
| Descriptive data | 14 | (a) Give characteristics of study participants (*e.g.*, demographic, clinical, social) and information on exposures and potential confounders  (b) Indicate the number of participants with missing data for each variable of interest  (c) *Cohort study* - summarise follow-up time (*e.g.*, average and total amount) | (a) Characteristics of study participants (sex and age) are given in Results, Descriptive Analysis, p13  (b) As above, Missing data on hospitalisation status is covered in Methods, Data, Serotype-specific case incidence (p8) and missing data on population sizes from the 2015 intercensal survey and 2020 census in Methods, Data, Population Data (p7). |  |  |
| Outcome data | 15 | *Cohort study* - Report numbers of outcome events or summary measures over time  *Case-control study* - Report numbers in each exposure category, or summary measures of exposure  *Cross-sectional study* - Report numbers of outcome events or summary measures | Total number reported Methods, Data, Epidemiological Data (p6), denominators provided whenever proportion given in Results, Descriptive Analysis (pp13-15). |  |  |
| Main results | 16 | (a) Give unadjusted estimates and, if applicable, confounder-adjusted estimates and their precision (e.g., 95% confidence interval). Make clear which confounders were adjusted for and why they were included  (b) Report category boundaries when continuous variables were categorized  (c) If relevant, consider translating estimates of relative risk into absolute risk for a meaningful time period | (a) Unadjusted estimates and confidence or credible intervals given throughout Results (pp13-19).  (b) Category boundaries reported for population groups when defined in Methods, Data, Population Data, pp7-8, and Methods, Mathematical Models, Non-serotype-specific Models, pp9-10.  (c ) NA |  |  |
| Other analyses | 17 | Report other analyses done—e.g., analyses of subgroups and interactions, and sensitivity analyses | Other modelling analysis described in Results, Non-serotype-specific-models, pp16-17 and Results, Serotype-specific Model, pp17-19. |  |  |
| **Discussion** | | | | | |
| Key results | 18 | Summarise key results with reference to study objectives | Discussion p19. |  |  |
| Limitations | 19 | Discuss limitations of the study, taking into account sources of potential bias or imprecision. Discuss both direction and magnitude of any potential bias | Discussion pp20-23. | RECORD 19.1: Discuss the implications of using data that were not created or collected to answer the specific research question(s). Include discussion of misclassification bias, unmeasured confounding, missing data, and changing eligibility over time, as they pertain to the study being reported. | Discussion pp20-23. |
| Interpretation | 20 | Give a cautious overall interpretation of results considering objectives, limitations, multiplicity of analyses, results from similar studies, and other relevant evidence | Discussion pp20-23. |  |  |
| Generalisability | 21 | Discuss the generalisability (external validity) of the study results | Discussion pp20-23. |  |  |
| **Other Information** | | | | | |
| Funding | 22 | Give the source of funding and the role of the funders for the present study and, if applicable, for the original study on which the present article is based | Funding is detailed separately in PLOS’s financial disclosure statement. |  |  |
| Accessibility of protocol, raw data, and programming code |  | .. |  | RECORD 22.1: Authors should provide information on how to access any supplemental information such as the study protocol, raw data, or programming code. | Methods, Data, Epidemiological Data, pp 6-7 and Data Availability, p13. |

*Reference: Benchimol EI, Smeeth L, Guttmann A, Harron K, Moher D, Petersen I, Sørensen HT, von Elm E, Langan SM, the RECORD Working Committee. The REporting of studies Conducted using Observational Routinely-collected health Data (RECORD) Statement. *PLoS Medicine* 2015; in press.

*Checklist is protected under Creative Commons Attribution ([CC BY](http://creativecommons.org/licenses/by/4.0/)) license.
