# Supplementary material for: Overall and serotype-specific dengue virus transmission intensity in Mexico, 2016–2023: A modelling study of case-notification data
Source: PLoS Med. 2026 Sep 3;23(9):e1004874. doi: 10.1371/journal.pmed.1004874 (PMC13541250; doi:10.1371/journal.pmed.1004874)
Supplement: Supplementary file 4 [file pmed.1004874.s006.docx]

**Point-by-point response letter**

**Overall and serotype-specific trends in dengue virus transmission in Mexico, 2016-2023**

Oliver Simmons, Anna Vicco, Ruth A. Martínez-Vega, José Ramos-Castañeda, Ilaria Dorigatti

**Comments from the academic editor:**

**1) I think there is clearly a large COVID effect on surveillance and potentially transmission, both overall, and for specific serotypes (e.g. Figure 1, 3, 4, and 5). The authors don't address this either in their model structures, or in interpretation of results, and I would like to see a little more consideration of this.**

We agree with the editor that COVID may have affected dengue surveillance and transmission, but given dengue virus (DENV) transmission in Mexico is cyclical (with large outbreaks typically occurring every 3-4 years due to complex immunological interactions), the reduction in reported case incidence seen in 2020, 2021 and 2022 cannot be attributed with certainty to the COVID pandemic, and may have been at least partially due to the build-up of immunity in the population that occurred in the large outbreak in 2019 (which was followed by a large dengue outbreak across many states in 2023, consistent with observed historical trends).

However, we did consider whether we could estimate and statistically test hypotheses around the COVID effect (e.g. reductions in transmission vs reduced surveillance and case reporting). Methodologically, the introduction of a COVID effect as either a reduction in transmission (acting on the force of infection [FOI]) or in reporting is not identifiable in the catalytic modelling framework which was used in this work. In fact, we can scale both or either the FOI and the reporting rate by a scaling factor during the pandemic years (representing a proxy for the pandemic effect) but the spatial and time-varying nature of the analysis imply that the other parameter estimates would balance out any reduction and ultimately generate the current estimates. Estimating the extent to which the COVID pandemic reduced transmission and/or reporting is feasible using transmission dynamics models (e.g. SIR-type compartmental models), which however require different data and are used to answer completely different questions from those investigated in this study.

We have therefore expanded the Discussion section of the paper to discuss the possible impacts of COVID-19 on dengue transmission, and discuss how this modelling framework can quantify the changes and variations in transmission and reporting including during the pandemic, but do not allow to disentangle the relative contribution of potential reductions in transmission and/or reporting due to the COVID restrictions vs natural variations in the FOI. We have now added these considerations to the Discussion (p20, lines 521-526):

*“The study period also included the COVID-19 pandemic, which may have affected reporting rates during 2020-2022 and could potentially have affected transmission intensity as well, given the restrictions in human mobility; however, the catalytic modelling framework used in this study does not allow us to quantify the mechanisms and extent to which the COVID-19 pandemic affected DENV circulation, which in future studies can be explored utilising mechanistic compartmental models calibrated to time-series data.”*
**2) In model structures, there are quite a number of assumptions for fixed parameters - I suspect that reviewers will pick up on these, and will likely ask the author for more sensitivity analysis, varying these assumptions.**

We did not include fixed parameters in our models, but in the original version of the paper, we had made the assumption that the estimated historical transmission intensity of dengue (and $\lambda_{H}^{\omega}$) were constant in time. In light of this comment and of the suggestions made by Reviewer #2, we have relaxed this assumption and in the revised version of the manuscript we estimated time-varying historical FOI estimates. We provide more details on the changes made on this point below (see our response to Reviewer #2, point 3).

**3) I tried to access the Github repository to review code and output, but this link is inaccessible currently. Important to review model convergence and diagnostics I think.**

We are unsure about the technical issues with the GitHub repository, as it is public and open access. If the issue with accessing the code persists, we would be delighted to work with the Editor to help and solve the issue.

As per request of Reviewer #3, we have now made it clear at the start of each modelling section of the results that the model converged for all states presented according to the diagnostic criteria outlined in the Methods section (Model Fitting, p13, lines 295-297). We have also now added trace plots of parameter estimates for all states for the three main models used in the paper (Models 1,2, and 4) to the GitHub repository, which can also be used to visually confirm model convergence.

**4) Minor point, but adding state labels to map figures would considerably aid interpretation of data for readers unfamiliar with the geography of Mexico.**

We thank the Editor for this suggestion, and we agree that this additional information is useful to readers. We have added a labelled map of the Mexican states in the Supplementary (Supplementary Figure S1), which can be used as reference to all other maps included in the paper.

**5) The authors should acknowledge and discuss the limitation around non-availability of serological data in more detail.**

We thank the editor for this suggestion and agree with them and Reviewer #4 that it is useful to discuss the advantages of serological data and case-notification data. We have updated the discussion substantially to cover this in more detail (pages 21-23, lines 538-586):

*“The serotype-specific version of the model, whilst being an improvement over the non-serotype-specific versions, is limited by data availability. Given only 10.3% of cases had a positive RT-PCR result, we reconstructed serotype-specific case data by scaling the total number of hospitalised and non-hospitalised cases by the respective proportions of each serotype observed from RT-PCR testing. As such, these reconstructed data have limitations, as we could not correct for potential differences in serotype-specific severity and therefore propensity to be RT-PCR-tested. Increased availability and use of validated RT-PCR assays capable of reliably distinguishing dengue serotypes in Mexico and across the world would help generate more accurate transmission intensity estimates for the different serotypes.*

*The availability of age-stratified seroprevalence data collected from prospective serological studies or residual samples at a local level (e.g. municipality, city or neighbourhood) would provide real-world evidence of the burden of DENV infection, and coupled with the reported case-notification data, would offer new opportunities to validate the reporting rate estimates generated in this study. Serotype-specific seroprevalence assays would also provide opportunities to validate serotype-specific FOI estimates, though the analysis and interpretation of serotype-specific antibody levels remains challenging due to cross-reactivity [1, 2]. To date, population-based seroprevalence surveys have been conducted in Morelos and Yucatán [3, 4], and a school-based survey has been conducted across 67 municipalities [5], which underlines the need for additional, systematically designed seroprevalence surveys, conducted in parallel to the surveillance system, to support robust state-level epidemiological inference.*

*Due to the limitations in serotype-specific data, we assumed that a serotype was not co-circulating in a state if there were fewer than 10 positive RT-PCR tests for that serotype in each modelled year– this assumption could potentially be affected by differing serotype-specific propensities to cause severe disease, hospitalisation, and RT-PCR-testing. We also assumed that if a serotype was not circulating between 2016-2023, it was also not circulating prior to 2016, given the catalytic modelling framework does not allow us to reconstruct whether high levels of population immunity versus no introduction drive the absence of circulation of a serotype. Going forward, it would be useful to investigate and disentangle the potential mechanisms behind heterogeneities in serotype circulation, particularly the role that prior immunity plays in the low incidence and modelled transmission intensity of DENV-4, which has the lowest FOI of all four serotypes in the eight states in which we modelled the transmission of this serotype, which is not consistent with previous serotype-specific FOI estimates obtained in Peru and French Polynesia [6, 7].”*

**Comments from the reviewers: 

Reviewer #1: Simmons et al present a detailed set of dengue surveillance data from Mexico and several models to describe the transmission dynamics. As someone who is interested in dengue, I liked the detailed descriptions of the data and following the serotype trends. In particular, the re-emergence of DENV-3 (likely caused by the newly introduced and emerging DENV-3III_B.3.2 lineage) was fascinating the see. The figures were mostly clear and visually appealing.**

We thank the Reviewer for their positive comments.

**My main criticism is that the work is mostly descriptive, and it is not very clear what we learn about dengue beyond 2016-2023 in Mexico. The conclusion mentioned that the work challenges the assumption of equal transmissibility between serotypes, but this knowledge gap or misconception was not well-established in the text (assuming that this comes from the Ferguson model, but my take on it is that it was for computational simplicity rather than a true epi process). Further, the discussion was also mostly descriptive and missed opportunities to compare the work with the vast dengue literature. I think that would help in making in more convincing to the reader the importance and novelty of the work.**

We thank the Reviewer for their suggestions and have taken the opportunity to better explaining the knowledge gap and novelty of the methods developed in this paper. Specifically, we have clarified the extent to which the assumption of equal transmissibility of serotypes is made in several previous modelling studies (e.g., in Imai et al. [8], Vicco et al. [9], and others [10-14]) and in turn how this assumption has been propagated in the current global transmission intensity map published by Cattarino et al. [8, 15]. This study challenges this assumption and, given the serotype-specific efficacy profile of dengue vaccines, serotype-specific FOI estimates can help improve analyses on the impact of vaccination in the future. We have clarified the knowledge gaps and novelty of this study in the Introduction of the paper as follows (p5, lines 114-118):

*“Previous modelling work on dengue using reported-case data typically assumes the equal circulation and transmissibility of all four serotypes [8, 11-13, 15], due to the absence of data on the number of cases caused by circulating serotypes. Whilst serotype-specific FOIs have been estimated, mainly from seroprevalence studies, none of these focussed on Mexico [6, 7, 10, 16-19].”*

**A moderate concern is that the details of the RT-PCR test to determine the serotype should be provided. There are many assays, and some have different but important limitations (such as not being able to detect specific genotypes that could bias the data). Moreover, broadly calling for increased RT-PCR for serotype discrimination requires nuance as there are many ways to do this wrong and not enough clarity on which assays perform the best**

We agree with the Reviewer that advocating for increased testing requires nuance and have amended the text as requested (lines 545-547, p21):

*“Increased availability and use of validated RT-PCR assays capable of reliably distinguishing dengue serotypes in Mexico and across the world would help generate more accurate transmission intensity estimates for the different serotypes.”*

As for the details of the RT-PCR tests used, diagnosis is carried out using commercial RT-PCR kits. There are no specific federal guidelines determining which tests should be used by state public health laboratories, so states typically buy kits at the best price. This is the case for both the first round of testing, to determine whether the patient is positive for ZIKV, DENV or CHIKV, and the serotyping done in a proportion of these DENV-positive cases [20]. An example RT-PCR used in the first round of testing is the *Applied Biosystems TaqMan Arbovirus Triplex Control Kit*. For serotyping, the central laboratory is part of the Latin American dengue surveillance network, so uses the CDC DENV-1-4 Real-Time RT-PCR Assay [21], but the test bought by states again depends on price.

With regards to not being able to detect specific genotypes: some commercially available kits fail to detect genotype III of DENV-4, and there have been reports of false negatives of the sylvatic genotype of DENV-2 [22]. However, none of these genotypes are known to have circulated in Mexico (as of 2025, using methods similar to Ramos- Castañeda et al. [23]).

**Minor concerns:

Should be clearer when referring to the disease/cases (dengue) and the virus (dengue virus). Descriptions of transmission, serotypes, and mosquito vectors should be attributed to the virus not the disease. This became better later in the text, but there are many examples of this misuse are throughout the abstract, first paragraph of the introduction, methods,**

We thank the Reviewer for highlighting these discrepancies. We have corrected them throughout the paper.
**Introduction: is immunity truly life-long against reinfection with the same serotype. My assumption is that the duration would be long-lasting but also variable. Ref 11 does not study this and another reference should be added to support this claim.**

We thank the Reviewer for pointing this out. We have now amended that sentence to read *“Upon infection, each serotype confers long-term immunity to that serotype, and short-term immunity to heterologous serotypes, though estimates of the duration of this immunity vary [24, 25]”.* We have clarified in the Methods section that lifelong immunity is a common modelling assumption that we have also made. **Suggest labeling lambda in the figures as FOI for clarity.**

We thank the reviewer for this suggestion and have amended Figure 2c and 4 specifying that the figures show the “per-serotype FOI”.

**I'm not sure how to make it better, but I found Figure 5a very difficult to follow. FOI, serotype, location, and year is a bit too much to pack into one set of panels. Perhaps something similar to Figure 3 would be better.**

We thank the Reviewer for this comment. We have redesigned Figure 5, and we feel confident that it is now easier to follow.

**For the future, adding line numbers would help the review process. Many times I wanted to reference a specific sentence to be clearer with my responses but couldn't.**

Apologies for the inconvenience, we had included line numbers in the original submission but there must have been a technical error. We have included line numbers in the revised version, and we will submit in PDF version too to avoid any potential issue.

**Reviewer #2: The article "Changes in dengue virus transmission in Mexico: 2016-2023", summarizes the spatial and temporal variation in dengue virus transmission at the serotype-specific level across Mexico from 2016 to 2023 using surveillance data. They found heterogeneity across Mexico with high transmission of DENV-1, DENV-2, limited circulation of DENV-4, and an increase in DENV-3 transmission in multiple states in recent years. This paper uses a large national dataset and addresses an important gap in the literature: how to measure the serotype-specific force of infection from case data. This paper is also important and relevant given the recent increase in dengue across South America. There are a few points of concern listed below, as well as some general suggestions.**

We thank the Reviewer for the positive feedback.

**Major Comments: 
1) Introduction: "The extent to which primary and post-primary (including tertiary and quaternary) infections are reported to surveillance can also vary, not only due to differences in disease but also in surveillance". This sentence is confusing, and it is not clear that reference 12 supports this point. If differences in reporting of primary and secondary infections are not only due to disease presentation, or reporting by age (which is also accounted for in the models) what would drive this difference in surveillance? The sentence seems circular.**

We agree that the sentence could be improved. The intent was to make the point that differences in disease severity due to antibody dependent enhancement can cause different propensities to report cases, and that the number of cases reported by different locations is affected by the sensitivity of the surveillance system (i.e. what proportion of the actual infections is reported). We cited Katzelnick et al.’s paper on ADE [26] to support the point that dengue severity varies by infection type, but we appreciate that this was unclear and we have now re-written the sentence to clarify the meaning (lines 95-98, p4):

*“This complex relationship between immunity and disease also makes evaluations of dengue’s disease burden difficult, and, due to differences in disease severity [26], the extent to which primary and post-primary (including tertiary and quaternary) infections are reported to surveillance can vary.”*

**A related point, in the Results section, "There was also substantial variation in the estimated secondary reporting rates (ρ) and the estimated reporting rates of primary cases relative to secondary reporting rates (γd), with ρ varying from 0.0051 (95% CrI 0.0046-0.0058) in Querétaro to 0.3245 (95% CrI 0.3207-0.3283) in Morelos, and γd from 0.0003 (95% CrI: 0.00001, 0.0018) in Coahuila to 0.9920 (95% CrI 0.9604-0.9997) in Chiapas." I am still unclear about the interpretation of variation in secondary reporting rates, and how this affects the FOI age assumptions. What could explain the variation across states reporting rates for primary versus secondary infection? Is it something to do with how severe cases are captured? Providing additional explanation for the possible causes would be helpful to the reader. Also, is data on IgM/IgG ratios available to help validate variation in primary versus secondary infections?**

We thank the reviewer for this question. Our estimates suggest a consistent trend across states whereby all reporting rates (primary relative o secondary, unconstrained primary and unconstrained secondary reporting rates) increase as the FOI estimates increase (see Figure 1a-c below). This implies that in Mexican states where DENV transmission is more established and the intensity of transmission is stronger, a larger number of the underlying (unobserved) number of infections is reported to surveillance. The modelling framework adopted in this study does not allow us to disentangle the mechanisms driving this observation, e.g. whether larger reporting is driven by increases in severity or whether medical professionals, the general public, or both are more aware and therefore likely to report dengue cases. Figure 1d (below) shows that the estimates of primary reporting rate relative to the secondary reporting rate are consistent across states, except for two outliers. Data on IgM and IgG ratios are not available in the National Epidemiological Surveillance System (SINAVE). We have amended the original text as follows (p16, lines 388-391):

*“Estimated secondary reporting rates (*$\rho$*) varied from 0.0051 (95% CrI [0.0046, 0.0058]) in Querétaro to 0.3245 (95% CrI [0.3207, 0.3283] in Morelos, with low estimated secondary reporting rates often associated with low estimates of per-serotype FOI.”*

Figure 1: Trends in per-serotype FOI and reporting rates from the time-constant non-serotype-specific model (Model 1). Colours indicate a wider regional grouping of states. The solid black line in a.-c. shows the fit of a linear regression, and the grey shaded area the 95% confidence interval.

**2) As written, it was not clear if all PCR-tested cases were serotyped. Further, as a reader, it wasn't clear until page 22 what proportion of cases were tested by PCR: "Among all cases, 85529 (10.3% of all probable or confirmed cases) tested RT-PCR positive for one of the four DENV serotypes between 2016 and 2023." Finally, what were the criteria for RT-PCR testing? Is it likely that they are representative, or are there biases in who was tested? Having more information on these points included in the methods section would he useful. It would also be helpful for the authors to explain the serotype-specific FOI estimates as conditional on assumptions about how representative the serotype-specific data are.**

The diagnostic framework implemented in Mexico is described in [20] and can be summarised as follows: at the beginning of the transmission season samples collected in the acute phase (less than 6 days after the onset of fever) from probable cases are tested by either a DENV NS1 or a multiplex qRT-PCR testing for DENV, ZIKV, and CHIK, depending on test availability and regardless of severity. When local cases have been reported, testing is done on 30% of probable non-severe dengue cases and on all suspected severe dengue cases and dengue cases with warning signs; after 30 days of no reported cases, testing focuses entirely on probable cases as is the case at the start of the transmission season. If either the NS1 or qRT-PCR test is positive, serotyping is then performed in a separate qRT-PCR reaction on 10% of the positive non-severe dengue cases and on all positive severe dengue cases and positive dengue cases with signs of alarm. If the sample was obtained after day 6, IgM capture is performed instead (and cases are not serotyped). Additional details are provided in Figure 4 of reference [13].

By surveillance design, testing and serotyping focuses on severe cases in Mexico, and for this reason we calculated the prevalence of each serotype by hospitalisation status. Figure 2 below (which is also included in the Supplementary Information) shows the serotype prevalence by age separately for hospitalised patients (a) and non-hospitalised patients (b). Notably, we did not assume that the serotypes detected in non-hospitalised patients are representative of hospitalised patients (or vice versa). Rather, we projected the relative serotype prevalence observed in each group (hospitalised and non-hospitalised) onto the respective proportions of hospitalised and non-hospitalised individuals with no serotype information, i.e. we assumed that the serotype-specific proportions detected among hospitalised (non-hospitalised) individuals is representative of the serotypes circulating among hospitalised (non-hospitalised) cases respectively.

We have added the following to the Methods (p7, lines 156-162) and pp8-9, lines 183-204):

*“The reverse transcription polymerase chain reaction (RT-PCR) test result (resultado PCR) was defined as either DENV-1, DENV-2, DENV-3, DENV-4, or no designated serotype (sin serotipo aislado). Not all probable cases are tested for DENV infection, and not all those positive for DENV have their serotype categorised, with the proportion of probable cases selected for testing dependent on the local dengue circulation according to surveillance and the case severity [20].*

*We reconstructed the number of serotype-specific cases from the total number of cases reported by state and the respective proportions of cases positive for each serotype among hospitalised and non-hospitalised cases separately. To account for the fact that severity impacts the likelihood a person receives an RT-PCR test [20], we stratified cases positive for a serotype by hospitalisation status and calculated the prevalence of each serotype separately for hospitalised and non-hospitalised cases for each year and state. We then assumed that the prevalence of each serotype among the probable/confirmed cases without a designated serotype was the same as among those with a designated DENV serotype, and reconstructed the number of hospitalised and non-hospitalised cases for each serotype by multiplying the total number of hospitalised and non-hospitalised cases by, respectively, the serotype-specific proportion positive for each serotype among hospitalised and non-hospitalised cases. We then combined these estimates for each state and year to generate the total reconstructed serotype-specific case incidence for each state and year.*

*We assumed that the serotype-specific distribution of the cases with unknown hospitalisation status (0.006% [53/833,360] of probable and confirmed cases) was the same as was observed among non-hospitalised cases. Building on the available age-distribution of serotype-specific case data (Figure S2 and Figure S3 in S1 Text), we assumed that the observed serotype proportions were constant over all ages. We reconstructed serotype-specific case incidence in a given state for the longest sequence of consecutive years for which there were more than 10 RT-PCR positive test results in each year. Furthermore, for a given state, we assumed that a given serotype was only circulating within these modelled years if were more than 10 RT-PCR positive cases for that serotype in at least in at least one of the years.”*

Figure 2: Proportion of RT-PCR-positive patients positive for each DENV serotype among (a) hospitalised patients and (b) non-hospitalised patients in 2016-2023 by state and age group.

**3) The authors assume that the serotype-specific and non-serotype-specific models have a constant level of dengue transmission before 2016. However, given what we know about long-term changes in dengue transmission in Mexico, including the changes that are related to urbanization and climate, does the assumption hold equally across all states in Mexico? This seems particularly problematic for serotypes that seem to not be observed for long periods at a time like DENV-3 and DENV-4: "This assumption also meant that if a serotype was not circulating between 2016- 2023, we assumed that it was also not circulating in the years before 2016, so any previous contribution of that serotype's historical circulation to population immunity profiles was not accounted for in the model, possibly biasing the initial susceptibility of the population and hence the FOI estimates generated."**

This is a great point, and we agree with the reviewer that the assumption of constant historic FOI (i.e. endemic transmission prior to 2016) could be relaxed. As a result, we performed a sensitivity analysis where we allowed the historic force of infections in both the time-varying non-serotype and serotype-specific models to vary over time. We assumed that the FOI was constant for 20- or 40-year periods of time, longer than annual period we assume for the years for which we have data. For the non-serotype-specific model, this improved the model fit for a majority of states over multiple performance criteria (DIC, WAIC, LOO-CV). Therefore, in the revised version of the manuscript we now present this model in the Results section of our paper and carried this model structure on for the serotype-specific model too. For consistency, the Methods section of the paper has also been changed to reflect this change in model structure. In general, the changes to the yearly FOI estimates generated by the new model were small, with the new model structure resulting in slightly lower FOI estimates in some years. For each state, the previous time-constant historic FOI was often around the midpoint of the new time-varying historic FOIs, and the magnitude of the new time-varying historic FOIs is consistent with the magnitude of the time constant FOI.

We also agree that the assumption that if a serotype was not circulating during the study period it was not circulating prior to 2016 poses limitations, but with no serology data, we are unable to discern whether the lack of circulation of a specific serotype is due to the absence of this serotype or is a result of high population immunity against that serotype due to a previous large outbreak. Further relaxing this assumption by adding a time-varying historic FOI for the unobserved serotypes would not, however, solve this issue due to fundamental identifiability issues in this question that cannot be disentangled with case data alone.

**4) The manuscript describes an increase in dengue transmission after COVID-19 but does not mention possible effects of the pandemic on surveillance and reporting. It would be helpful to comment on anything known about the effect on surveillance and possible effect it may have on estimates during the pandemic, especially 2020 and 2021.**

We thank the reviewer for this suggestion. We have now added a discussion of the possible effects of the pandemic on surveillance and reporting as suggested (lines 521-526):

*“The study period also included the COVID-19 pandemic, which may have affected reporting rates during 2020-2022 and could potentially have affected transmission intensity as well, given the restrictions in human mobility; however, the catalytic modelling framework used in this study does not allow us to quantify the mechanisms and extent to which the COVID-19 pandemic affected DENV circulation, which in future studies can be explored utilising mechanistic compartmental models calibrated to time-series data.”*

**5) This manuscript makes a novel contribution of estimating the time-varying serotype-specific force of infection from case data. It would be helpful to provide more literature review and comparison to what has been done previously for serotype-specific models using serology data.**

We thank the reviewer for their suggestion, and we have added the following to the Introduction (p5, lines 114-118):

*“Previous modelling work on dengue using reported-case data typically assumes the equal circulation and transmissibility of all four serotypes [8, 11-13, 15], due to the absence of data on the number of cases caused by circulating serotypes. Whilst serotype-specific FOIs have been estimated, mainly from seroprevalence studies, none of these focussed on Mexico [6, 7, 10, 16-19].”*

**6) Introduction: "Previous studies have suggested that the burden of dengue in Mexico may worsen further over the coming years due to climate change(7), although the extent and geographical distribution of the predicted change in dengue transmission differs between models(3, 8)." The present manuscript nicely measures annual changes in the force of infection of dengue across Mexico but does not connect to some of the bigger questions about effects of climate change. It would be helpful to return to this point and discuss potential implications in the discussion.**

We appreciate the suggestion and given we have not investigated how changes in climate may drive changes in the FOI, we agree to comment on the potential role of climate change and possible implications. We have now added the following sentence (p19, lines 499-501):

*“The impact of climate change on DENV transmission intensity in the modelled period is uncertain, and, in the future, the methods developed in this study could be extended to incorporate climactic factors as drivers of the changes in DENV FOI.”*

**Minor comments:
1) It would be helpful to build slightly more intuition for what the force of infection is for non-expert readers when the concept is first introduced.**

We thank the reviewer for this suggestion and have added amended the Introduction to now read (lines 103-108, p5):

*“The burden of dengue can, however, be estimated from the age-distribution of the reported case data through mathematical models. An important measure of dengue virus’s transmission intensity is the rate at which susceptible people become infected, known as the force of infection (FOI). The FOI of DENV can be estimated using a class of mathematical models referred to as catalytic models, first used to model DENV transmission dynamics by Ferguson et al. [19], using either seroprevalence or case-notification data [8, 10, 12]. ”*
**2) Throughout the manuscript, figures are not cited in order. For instance, in the methods, Figs. S5 and S6 are cited first in the text. Also, Fig. 2a is cited right after Fig. 1a. This is unconventional, and it would aid the reader if the figures were cited in the order they appear in the text.**

Apologies for this oversight, we have fixed the order of the figures in the text throughout the manuscript.

**3) Methods: "The explored four variants (Model 2-5) of the time-varying catalytic model developed by O'Driscoll et al." The heading to this section says "non-serotype specific models", but it was confusing because the four models were referred to as the 'explored four variants', which sounded like a serotype-specific model. Consider changing the wording to "Explored four different versions of the time varying catalytic model".**

Thank you for this. We have changed the sentence as suggested.

**4) Methods: "We only reconstructed the serotype-specific case incidence in a given state for years that had more than 10 RT-PCR-positive test results, and we assumed that a DENV serotype was present in a state if there were more than 10 RT-PCR-positive results for that serotype in at least one of the modelled years (2016-2023 inclusive)." This is confusing. Does this mean if a state had 10 dengue cases, 2 DENV-1 and 8 DENV-2, then even though it meets the first rule, you wouldn't reconstruct the case data? It would be helpful to add a sentence to better explain when the serotype-reconstruction is performed.**

We thank the Reviewer for pointing out that this sentence needed clarification. We have reworded the sentence as shown in lines 200-204 (Methods, Serotype-specific case incidence, pp8-9):

*“We reconstructed serotype-specific case incidence in a given state for the longest sequence of consecutive years for which there were more than 10 RT-PCR positive test results in each year. Furthermore, for a given state, we assumed that a given serotype was circulating within the modelled years only if were more than 10 RT-PCR positive cases for that serotype in at least one of these years.”*

**5) Methods: "as we removed those under 1 year old from the analysis". Further down in the text, it is explained that the authors removed "children younger than 1 year old due to maternally transferred immunity". It would be helpful if this point could be added further up in the text when this is first introduced.**

We have moved the explanation for our modelling choice further up in the text (p10, lines 235-236) as suggested.

**6) Models: Why introduce the scaling factor in equation 11 for different reporting rates by age and not further up in equation 2, when the concept is first introduced?**

We agree and have amended equation (2) as suggested.

**7) What are the colors in Fig. 5b?**

We have changed Figure 5 to improve legibility and interpretability and have changed the previous colour scheme. We feel confident this has improved Figure 5.

**8) "We were able to reconstruct the estimated age-distribution of the serotype-specific data (see Methods) using the serotype-specific model in 23 out of 26 states." It would be helpful to list the 3 excluded states. It wasn't clear why some were excluded that had high numbers of cases.**

With the revised model, we only excluded a single state (Yucatán), for which we had sufficient serotype-specific data only for two consecutive years (2022: 1091 cases, 391 RT-PCR positive and 2023: 44259 cases, 4731 RT-PCR positive).

**Reviewer #3:**

**This paper provides various models for the transmission of dengue virus (DENV) in Mexico.**

**I was asked for a methodological report and I interpret that to include all aspects of the design and conduct of the study. The version I received did not have page numbers. My numbering has the abstract starting on page 1.**

**Points of detail**

**Page 1 The title seems rather terse. As I understand it much of the novelty here is the investigation of the four serotypes of DENV but the casual reader of the title would not pick that up.**

We thank the reviewer for this suggestion and have updated the title to “*Overall and serotype-specific trends in dengue virus transmission in Mexico, 2016-2023.”*

**Page 4 Given the small number of people with missing ages it probably does not matter what was imputed for them but the strategy here does seem rather crude. I would have been inclined just to remove them.**

We agree with the reviewer that the small number of NAs in the 2015 intercensal survey (0.07% of people) mean that it does not make a great difference what was imputed for them, and we also agree that our strategy was simple. On balance, given both the strategy suggested by the Reviewer and our strategy are crude, we propose to stick to our original solution which is slightly less crude.

**Page 5 If the serotypes lead to disease of different severity then the assumption that serotype prevalancce is the same for hospitalised cases and non–hospitalised seems dubious. Do we know that the disease severity is the same?**

We agree with the reviewer that assuming that serotype prevalence was the same for hospitalised and non-hospitalised cases would have been problematic, and in fact we did not do that. We allowed the serotype prevalence to differ by hospitalisation status, calculated the prevalence of each serotype in each of these groups (hospitalised and non-hospitalised) separately, and we then assumed that the serotype prevalence among all hospitalised cases was the same as the serotype prevalence among hospitalised cases for which we have the serotype result, and that the prevalence of each serotype among non-hospitalised cases was the same as the prevalence of each serotype among non-hospitalised RT-PCR positive cases. We have reworded the paragraph explaining this more clearly in the text to aid clarity. It now reads as below (lines 183-198, p8):

*“We reconstructed the number of serotype-specific cases from the total number of cases reported by state and the respective proportions of cases positive for each serotype among hospitalised and non-hospitalised cases separately. To account for the fact that severity impacts the likelihood a person receives an RT-PCR test [20], we stratified cases positive for a serotype by hospitalisation status and calculated the prevalence of each serotype separately for hospitalised and non-hospitalised cases for each year and state. We then assumed that the prevalence of each serotype among the probable/confirmed cases without a designated serotype was the same as among those with a designated DENV serotype, and reconstructed the number of hospitalised and non-hospitalised cases for each serotype by multiplying the total number of hospitalised and non-hospitalised cases by, respectively, the serotype-specific proportion positive for each serotype among hospitalised and non-hospitalised cases. We then combined these estimates for each state and year to generate the total reconstructed serotype-specific case incidence for each state and year.*

*We assumed that the serotype-specific distribution of the cases with unknown hospitalisation status (0.006% [53/833,360] of probable and confirmed cases) was the same as was observed among non-hospitalised cases.”*

**Page 6 My ageing eyes found it hard to read some of the formulas and equation 2 is a good example. A thin space between the terms like p2 a∈˜Aj I2,a,t would help to see where one ends and the next one begins**

We thank the reviewer for this suggestion and agree that equation 2 could be improved. We have revised this in the reviewed version.

**Page 7 I do not understand the significance of \ in some of the subscripts. Is this the set complement operator?**

The “\” used in subscripts refers to set difference, i.e. $\Omega\backslash\{\omega\}$ is all the elements in $\Omega$ except $\omega$, in this case all serotypes except serotype $\omega$. We have explicitly stated what we mean by this when it appears (lines 257-258, Methods, Serotype-specific model, p11).

**Page 8 It might be better to move the justification for omitting those below one year to before the mention of the omission on page 6.**

We have updated this as recommended by the reviewer.

**Page 9 The results of the convergence test do not seem to be reported in the results. A simple sentence confirming they converged is enough.**

We have added sentences to each section of the Results concerning modelling to confirm convergence, and we have uploaded convergence plots to the GitHub repository.

(line 384, Results, Non-serotype-specific models, Time-constant model, p16):

*“Model 1 converged for all 27 states modelled.”*

(lines 399-400, Results, Non-serotype-specific models, Time-varying models, p16):

*“As a result, we present the results of Model 2 below (which converged for all states according to the tests set out in Methods).”*

(lines 433-436, Results, Serotype-specific Model, p17):

*“We reconstructed the age-distribution of the serotype-specific data (see Methods) using the serotype-specific model in 25 states (with the model successfully converging in all these states).”*

**Page 9 Naming the packages used is a good idea but if any of them is crucial to the modelling rather than data manipulation a full citation would be better.**

Thank you for sharing this comment. *rstan* is the only package crucial for modelling and is fully cited in the paper. The other packages (*tidyverse, loo, sf, MetBrewer, patchwork and binom*) are only used for data visualisation, manipulation, and statistical testing.

**Page 10 I can understand why on page 2 we have confidence intervals as those are presumably quoted from other sources but when we start to see the results why do we start with confidence intervals here before moving on to credible intervals on page 14? Is this just a typo or is there some reason?**

The Reviewer is correct that in the Introduction section we use confidence intervals as quoting results from other papers. The descriptive analysis sub-section of the Results contains confidence intervals because uncertainty was calculated using the exact Binomial confidence interval (using the Clopper-Pearson method), whilst in the modelling work we used Bayesian inferential methods to reconstruct the uncertainty of the parameters and estimates, which were therefore quantified in terms of credible intervals. We appreciate that it may be slightly inconsistent to switch from frequentist to Bayesian methods in the same paper, but these apply to two separate sections so we believe is acceptable. For completeness, we have added the following details to the data section of the Methods:

*“The case fatality rate (CFR) was calculated as the proportion of confirmed and probable cases who were recorded as having died, and incidence as the total number of probable and confirmed cases per capita per year, with the population of each state each year calculated as below. For the descriptive analysis, uncertainty was calculated using exact binomial confidence intervals (Clopper-Pearson method).”*

**Page 12 Is it possible to remove the distracting international boundaries in Central America? Removing the US as well would do no harm.**

We thank the reviewer for this suggestion and have removed all international boundaries from maps figures.

**Page 12 The caption could explain why there are grey areas in sub–panels c and d but not a and b.**

We thank the reviewer for this suggestion and have added the following sentence to the caption of Figure 3 (previously Figure 2) to explain this (lines 371-373, p15):

“Fig 3c and Fig 3d show the results of modelling work, with Baja California, Ciudad de México, Chihuahua, Tlaxcala, and Zacatecas in grey because they were excluded due to low dengue incidence.”

**Page 14 Figure 2 presents the states in alphabetical order of their names. Would it emphasise the results if they were grouped according to the pattern of the serotypes over time? The caption might point out the M´exico here is not the country as a whole nor Ciudad de M´exico but a state named M´exico. At least I assume that is the case. Not all readers will be as familiar with local geography as the authors.**

We thank the reviewer for their suggestion as to how to reduce confusion surrounding the state of México and the country of Mexico and have added the following sentence to the caption of Figure 2 (previously Figure 3) (p15, line 356): *“Note: México here refers to the State of México (one of Mexico’s 32 states), not the country itself.”* This change was made in addition to the provision of Supplementary Figure S1, which shows the different Mexican states. We tried to re-arrange the figure to reconstruct the rough geographic shape of the Mexican states, but this unfortunately affects the size of the panels and the legibility of the figure (see Figure 3 below) so we sticked to the original presentation of the plot.

Figure 3: Serotype prevalence by year and state in Mexico. ISO codes for state names are used.

**Page 18 I did not find this very informative. Because some states had very extreme values of FOI the remainder are very compressed and hard to distinguish. The state names in sub–panel a do not appear well aligned with the data points.**

We have changed the way the results are presented in Figure 5, which fixed these problems.

**Page 20, Figure 7 Usually to compare two quantities one plots one against the other or some function(s) of the two quantities. The current presentation rather obscures the fact that Michoac´an is rather different from the four mentioned in the text since they all have the discrepant points in the opposite order. I suggest plotting the FOIs against one another with the credible intervals as cross–hairs and code year in some way perhaps using colours on a continuum.**

We appreciate the suggestion to visually show the consistency of the estimates obtained with the two models as done in Figure 4 below. However, we find that Figure 4 makes it harder to compare the FOIs obtained with the two models by year and states, which is the point of this analysis. On balance, given this is a stylistic preference, we would prefer to stick to the original version of the figure in the main text, but we have now included Figure 5 below in the Supplementary Information (as Figure S33) for completeness.

Figure 4:Comparison between total force of infection estimates between the best performing non-serotype-specific model (Model 2) and the serotype-specific model (Model 4), for each modelled year for states fitted to more than 5 years of data (and also Campeche, which was fitted to 5 years of data). Points show the median total FOI estimate, and error bars 95% credible intervals. Total force of infection estimates were calculated by summing the FOI estimates of all serotypes (in the case of the serotype-specific model) or by multiplying the per-serotype FOI estimate by four (in the case of the non-serotype-specific model).

**Page 21 and elsewhere If the authors wish to compare with previous work it would be good to make clear which estimates are which. If in fact the estimates given are only those from the present work then please quote the previous ones to save readers having to open several articles to see what they were.**

We have re-written parts of this paragraph to hopefully make it clearer. Estimates from Vicco et al. were already directly quoted, and we have added the range of estimates from Rodriguez-Barraquer et al. from the introduction (which are now in both the Introduction and Discussion) [9, 11]. We have chosen not to include the estimates from O’Driscoll et al. [12], as they do not relate to Mexico.

**Page 23 The database limitations to which the authors allude here seem to me to be hard to avoid when using routine data. That should not stop us trying of course.**

We agree with the reviewer here.

**As a very minor point the authors are not consistent about place names. We have the state names using the Spanish orthography everywhere. For example Yucat´an not Yucatan but Mexico and Peru in some places. Unless PLOS has a policy on this I would suggest keeping to one approach. Personally I would prefer the Spanish orthography. At least in the UK there has been a gradual move to using local orthography over the last decades. I would remove Entidad from the caption in Figure 5 though.**

Thank you for this suggestion, we agree with the Reviewer that “*Entidad*” should be removed from Figure 5 (though since Figure 5 has been changed this is no longer an issue). We have been consistent with orthography, using Spanish orthography for state names, and English orthography for the names of countries. Our logic was that since “Mexico” and “Peru” are words in English, it makes sense to use their English spellings. However, since Yucatán and Querétaro, for example, are not, we prefer to use the Spanish spelling. We agree that if PLOS has a policy on this, we should adhere to that.

**Looking at the relevant publication guideline which I believe to be RECORD (Benchimol et al., 2015) I cannot see major omission but I think recognition of those guidelines would be helpful.**

We thank the Reviewer for pointing out that we missed this out. We have now added the following to the Method section (lines 137-139, p6):

*“This study is reported as per the Reporting of Studies Conducted Using Observational Routinely-Collected Data (RECORD) guidelines (S1 Checklist).”*

We will also be adding the completed STROBE/RECORD checklist as Supporting Information (S1 Checklist).

**Concluding remarks**

**The main message I draw from reading this is that there is massive heterogeneity. The authors have done a great deal of work to establish the facts but I did not find much explanation of possible moderator variables here. I am not though an expert in virology and have very limited knowledge of the physical or human geography of M´exico.**

**Michael Dewey**

We thank Prof Michael Dewey for revising our manuscript and the valuable suggestions.

**Reviewer #4: This study characterizes the spatial and temporal variations in dengue force of infection (FOI) across 27 states in Mexico from 2016 to 2023, utilizing confirmed/probable annual dengue cases (total of 833761) reported to the surveillance system and previously established non-serotype-based time-constant and time-varying catalytic models. Authors also report variations of the catalytic model they developed to allow serotype-specific time-varying forces, using the 85529 RT-PCR-positive cases to characterise differences in FOI across dengue serotypes. Overall, the manuscript is well written, and the descriptive analyses are well presented in Figures 1 and 3. While I believe the manuscript is valuable and worthy of publication in PLOS Medicine, I have also noted several limitations that must be addressed.**

We thank the reviewer for sharing their feedback and we are delighted to take this opportunity to discuss the identified limitations, and how this study contributes to challenging and expanding traditional modelling assumptions making best use of existing data.

**Their findings from the data modeling are undoubtedly the best use of the available RT-PCR data. However, it may not inform the actual epidemiological situation of dengue in Mexico. This is because the non-serotype-based time-constant and time-varying catalytic models used in the manuscript rely on presumptive and RT-PCR data, rather than serological testing, and on age-based stratification. The inability to distinguish primary from secondary infections and the exclusion of asymptomatic and mild subclinical cases can also lead to an underestimation of the true infection rate. While the models employed may still provide valuable insights into symptomatic cases (laboratory-confirmed and presumptive), the findings are not fully representative of the overall epidemiological situation of dengue, and this must be acknowledged.**

We thank the reviewer for sharing their concerns. It looks like there might have been some confusion in how the available data have been used to reconstruct the disease burden. In fact, despite the lack of reporting of asymptomatic and mild subclinical cases in the data, our model is robust to under-reporting as we modelled the observed symptomatic case data captured by surveillance through the modelling of case reporting, i.e. by modelling symptomatic cases as a proportion of the overall number of (unobserved) infections which includes asymptomatic and mild subclinical cases. Similarly, whilst the data do not distinguish between primary and secondary cases, our models do capture primary and secondary infections as shown in equation (2) and equation (12). In other words, we model both the incidence of primary and secondary dengue infection and asymptomatic and mild subclinical infections, and by assuming separate primary and secondary case reporting rates (which takes into account differences in severity), we reconstruct the proportion of infections that are reported to health authorities in each age group. This is a key motivation for the use of the models developed in this study to reconstruct the FOI which is needed to capture the unobserved infection and observed disease burden.

**Additionally, the non-serotype version of the catalytic models assumes that the four DENV serotypes are equally transmissible. This poses a significant limitation as asymptomatic dengue infections can vary by serotype.**

The assumption of equal transmissibility of the four serotypes is a long-standing modelling assumption which dates back to Ferguson et al. 1999 [19], and has been carried forward in numerous papers since (e.g. Imai et al. [8], Rodriguez-Barraquer et al. [11], Cattarino et al. [12, 15], and others [9, 10, 12-14]) in aid of model parsimony and in light of the lack of serotype data to reconstruct serotype-specific differences in FOI. Whilst the Reviewer hints at differences in severity (i.e. in the extent to which the asymptomatic to symptomatic ratio varies by serotype) there is no consensus surrounding potential differences in the transmissibility of the different serotypes, which this study focuses on. In other words, a key contribution of this paper is that it challenges an established assumption in dengue modelling, as we estimate transmission intensity using non-serotype-specific catalytic models and then compare our estimates to those generated using serotype-specific models that relax the historical assumption. What the Reviewer points to as a limitation, is one of the key insights the paper provides as it generates data-driven evidence around serotype-specific differences in transmission which local, multi-national, and global studies (e.g. Cattarino et al. [15]) do not account for.

**Previous, mostly cohort-based, studies showed that DENV-1 and DENV-4 are more likely to be asymptomatic, whereas DENV-2 is frequently symptomatic. However, these patterns may also vary depending on previous immunity, population genetics, and local viral strains. I appreciate that the authors acknowledged these limitations and, indeed, attempted to improve these models by developing serotype-specific variants using serotype-specific RT-PCR results. Yet it is the "best use of the existing data" scenario, but the findings reported remain limited by the lack of serology data.**

We agree that serological surveys on top of case data might give additional insight on dengue transmission in Mexico, unfortunately serotype-specific serological data have not been published. There is also no consensus around differences in the propensity of the different serotypes to cause asymptomatic and symptomatic infections, as different settings provide evidence of contrasting results, as shown for instance in Brazil [27], Nicaragua [28], Singapore [29], and Thailand [30, 31]. We also note that the analysis and interpretation of serotype-specific serological data is not trivial (with the exception of primary infection data). To capture the Reviewer’s comment, we have added the following text to the discussion (lines 562-573, p22):

*“The availability of age-stratified seroprevalence data collected from prospective serological studies or residual samples at a local level (e.g. municipality, city or neighbourhood) would provide real-world evidence of the burden of DENV infection, and coupled with the reported case-notification data, would offer new opportunities to validate the reporting rate estimates generated in this study. Serotype-specific seroprevalence assays would also provide opportunities to validate serotype-specific FOI estimates, despite the analysis and interpretation of serotype-specific antibody levels remaining challenging due to cross-reactivity [1, 2]. To date, population-based seroprevalence surveys have been conducted in Morelos and Yucatán [3, 4], and a school-based survey has been conducted across 67 municipalities [5], which underlines the need for additional, systematically designed seroprevalence surveys, conducted in parallel to the surveillance system, to support robust state-level epidemiological inference.”*

**Authors stated that "we assumed that the serotypes of distribution of the cases with unknown hospitalization status were the same as was observed among non-hospitalized cases." I believe this assumption can be challenged because DENV2 transmission following the ZIKV wave in the Pacific has driven more hospitalizations than historical DENV2 transmission. Additionally, genotypic variation among dengue serotypes can disproportionately influence disease severity, as observed during the circulation of American and Asian/American DENV2 in Peru following a DENV1 outbreak. Considering this, can the authors compare the serotype of distribution among hospitalized and non-hospitalized symptomatic cases?**

We have indeed stratified cases by hospitalisation status and estimated the prevalence of each serotype among hospitalised and non-hospitalised cases separately to account for exactly what the Reviewer refers to in this point. The quoted sentence refers to the serotype distribution among the very small number of probable/confirmed dengue cases which had NA hospitalisation status (0.006%, 53/833630) – in this instance we assumed that the serotype distribution was the same as among non-hospitalised cases. We have rewritten the paragraph to aid clarity. It now reads as below (lines 183-198, p8):

*“We reconstructed the number of serotype-specific cases from the total number of cases reported by state and the respective proportions of cases positive for each serotype among hospitalised and non-hospitalised cases separately. To account for the fact that severity impacts the likelihood a person receives an RT-PCR test [20], we stratified cases positive for a serotype by hospitalisation status and calculated the prevalence of each serotype separately for hospitalised and non-hospitalised cases for each year and state. We then assumed that the prevalence of each serotype among the probable/confirmed cases without a designated serotype was the same as among those with a designated DENV serotype, and reconstructed the number of hospitalised and non-hospitalised cases for each serotype by multiplying the total number of hospitalised and non-hospitalised cases by, respectively, the serotype-specific proportion positive for each serotype among hospitalised and non-hospitalised cases. We then combined these estimates for each state and year to generate the total reconstructed serotype-specific case incidence for each state and year.*

*We assumed that the serotype-specific distribution of the cases with unknown hospitalisation status (0.006% [53/833,360] of probable and confirmed cases) was the same as was observed among non-hospitalised cases.”*

**The authors claimed that their model predicted that DENV-1 and DENV-2 circulated at high levels, with DENV-4 transmission intensity low during 2016 to 2023 across Mexico. They also noted that DENV-3 transmission intensity has increased in some Mexican states over the past few years. Given the lack of serological data, the authors should limit their findings to symptomatic RT-PCR-confirmed cases.**

We have highlighted more in the paper that our model relies on and is fitted to symptomatic RT-PCR confirmed cases, but the underlying structure of the model includes both symptomatic and asymptomatic cases and fundamentally reconstruct the unobserved infection burden. As discussed above, since the estimates of transmission intensity capture the unobserved infection burden of dengue (including symptomatic and asymptomatic infections), it would be incorrect to interpret the FOI estimates as pertaining to symptomatic RT-PCR-confirmed cases only. Catalytic models calibrated to only case data (e.g. Imai et al. [8], and Rodriguez-Barraquer et al. [11]) and to only seroprevalence data (e.g. Imai et al. [10], and Vicco et al. [9]) provide FOI estimates which are equally valuable, interpretable and comparable, and in fact these are often pooled and merged in global modelling studies (Cattarino et al. [15]). In other words, the FOI is a metric that allows to draw meaningful comparisons on the intensity of transmission across locations (regardless of whether FOI estimates have been obtained from case-notification or serological data) which is not possible to do using case data alone, due to differences in surveillance and reporting.

**Additionally, I suggest a modification in the abstract that states "This work quantifies serotype-specific differences in dengue transmission intensity using routinely collected case-notification data and demonstrates how extensive RT-PCR testing and new rapid diagnostic tests capable of discerning infecting serotypes can help to better understand the contributions of different serotypes to transmission." I am particularly concerned about the use of RT-PCR to estimate transmission rates, given that dengue viremia is brief, is estimated to miss roughly 50% of symptomatic cases, and the asymptomatic infections are also unaccounted for.**

We agree that there is a limited time window in which RT-PCR can pick up the infecting serotype, and we appreciate the Reviewer’s concern. However, because we do not calibrate the model to incidence data (which is indeed affected by under-reporting) rather we fit to the age-distribution of the case data (which is not affected by under-reporting), the estimates that we generate are not affected by under-reporting. As discussed above, our model reconstructs the unobserved number of primary and secondary, symptomatic and asymptomatic infections, and captures how different proportions of these infections generate the observed disease data. In summary, since we are calibrating the model to the age-distribution of the RT-PCR data (not the incidence), the transmission intensity estimates generated in the study are robust to under-reporting.

**Authors mention that "Serotype-specific seroprevalence studies would be instrumental to disentangle the potential mechanisms behind the heterogeneities in serotype circulation." It would be beneficial to add a discussion of the serological tools that could power future studies like theirs. They should emphasize that performing serology on an acute specimen is the best way to identify past infection and can distinguish primary from secondary dengue symptomatic infections. Serosurveillance using serological assays capable of distinguishing primary dengue serotypes with convenient yearly sampling can cover both asymptomatic and symptomatic dengue infections and distinguish primary from secondary dengue infections. Acknowledging the existence of such serological tools could also be beneficial for advancing the field.**

We thank the reviewer for this suggestion, and have added the following to the Discussion (lines 545-547 and 562-567, pp21-22):

*“Increased availability and use of validated RT-PCR assays capable of reliably distinguishing dengue serotypes in Mexico and across the world would help generate more accurate transmission intensity estimates for the different serotypes.*

*The availability of age-stratified seroprevalence data collected from prospective serological studies or residual samples at a local level (e.g. municipality, city or neighbourhood) would provide real-world evidence of the burden of DENV infection, and coupled with the reported case-notification data, would offer new opportunities to validate the reporting rate estimates generated in this study. Serotype-specific seroprevalence assays would also provide opportunities to validate serotype-specific FOI estimates”*
**Additionally, previous studies have noted that homologous reinfection and heterologous asymptomatic infections can contribute to DENV transmission, as infected individuals with high viremia may facilitate transmission. Authors should acknowledge that future epidemiological models should incorporate homologous re-infection and heterologous inapparent infections to more accurately estimate DENV exposure and transmission.**

We thank the Reviewer for this suggestion and agree that our models do not account for homologous reinfections, and that this is something that should be investigated more in the future. However, our models make no assumption about heterologous asymptomatic infections’ contribution to DENV transmission – what the Reviewer hints to can be explored using mechanistic models of transmission, not catalytic models like the ones we have utilised in this analysis. We have added the following sentence to the discussion on this point (lines 534-538, p21):

*“In this study we assumed that serotype-specific infection provides long-lived immunity against reinfection, i.e. that homologous reinfection does not occur. Whilst this is a consolidated modelling assumption, reinfections have been reported to occur in some studies, and in future work it will be interesting to explore the extent to which modelling homologous reinfections allow us to better capture the observed data [32].”*

## References

1.	Sangkaew S, Tan LK, Ng LC, Ferguson NM, Dorigatti I. Using cluster analysis to reconstruct dengue exposure patterns from cross-sectional serological studies in Singapore. Parasites & Vectors. 2020;13(1):32. doi: 10.1186/s13071-020-3898-5.

2.	Cracknell Daniels B, Buddhari D, Hunsawong T, Iamsirithaworn S, Farmer AR, Cummings DAT, et al. Predicting the infecting dengue serotype from antibody titre data using machine learning. PLOS Computational Biology. 2024;20(12):e1012188. doi: 10.1371/journal.pcbi.1012188.

3.	Amaya-Larios IY, Martinez-Vega RA, Mayer SV, Galeana-Hernandez M, Comas-Garcia A, Sepulveda-Salinas KJ, et al. Seroprevalence of neutralizing antibodies against dengue virus in two localities in the state of Morelos, Mexico. Am J Trop Med Hyg. 2014;91(5):1057–65. Epub 20141006. doi: 10.4269/ajtmh.14-0145. PubMed PMID: 25294613; PubMed Central PMCID: PMCPMC4228874.

4.	Pavia-Ruz N, Diana Patricia R, Salha V, Granja P, Balam-May A, Longini IM, et al. Seroprevalence of Dengue Antibodies in Three Urban Settings in Yucatan, Mexico. Am J Trop Med Hyg. 2018;98(4):1202–8. Epub 20180215. doi: 10.4269/ajtmh.17-0382. PubMed PMID: 29460714; PubMed Central PMCID: PMCPMC5928812.

5.	Amaya-Larios IY, Rojas-Russell M, Lopez-Cervantes M, Castro-Porras L, Castro-Borbonio MV, Sarti E, et al. Seroprevalence of dengue in school children in Mexico ages 6-17 years, 2016. Trans R Soc Trop Med Hyg. 2018;112(5):223–9. doi: 10.1093/trstmh/try046. PubMed PMID: 29917129; PubMed Central PMCID: PMCPMC6030845.

6.	Reiner RC, Jr., Stoddard ST, Forshey BM, King AA, Ellis AM, Lloyd AL, et al. Time-varying, serotype-specific force of infection of dengue virus. Proc Natl Acad Sci U S A. 2014;111(26):E2694–702. Epub 20140520. doi: 10.1073/pnas.1314933111. PubMed PMID: 24847073; PubMed Central PMCID: PMCPMC4084484.

7.	Nemoto T, Aubry M, Teissier Y, Paul R, Cao-Lormeau V-M, Salje H, et al. Reconstructing long-term dengue virus immunity in French Polynesia. PLOS Neglected Tropical Diseases. 2022;16(10):e0010367. doi: 10.1371/journal.pntd.0010367.

8.	Imai N, Dorigatti I, Cauchemez S, Ferguson NM. Estimating Dengue Transmission Intensity from Case-Notification Data from Multiple Countries. PLoS Negl Trop Dis. 2016;10(7):e0004833. Epub 20160711. doi: 10.1371/journal.pntd.0004833. PubMed PMID: 27399793; PubMed Central PMCID: PMCPMC4939939.

9.	Vicco A, McCormack C, Pedrique B, Ribeiro I, Malavige GN, Dorigatti I. A scoping literature review of global dengue age-stratified seroprevalence data: estimating dengue force of infection in endemic countries. EBioMedicine. 2024;104:105134. Epub 20240507. doi: 10.1016/j.ebiom.2024.105134. PubMed PMID: 38718682; PubMed Central PMCID: PMCPMC11096825.

10.	Imai N, Dorigatti I, Cauchemez S, Ferguson NM. Estimating dengue transmission intensity from sero-prevalence surveys in multiple countries. PLoS Negl Trop Dis. 2015;9(4):e0003719. Epub 20150416. doi: 10.1371/journal.pntd.0003719. PubMed PMID: 25881272; PubMed Central PMCID: PMCPMC4400108.

11.	Rodriguez-Barraquer I, Salje H, Cummings DA. Opportunities for improved surveillance and control of dengue from age-specific case data. Elife. 2019;8:e45474. Epub 20190523. doi: 10.7554/eLife.45474. PubMed PMID: 31120419; PubMed Central PMCID: PMCPMC6579519.

12.	O'Driscoll M, Imai N, Ferguson NM, Hadinegoro SR, Satari HI, Tam CC, et al. Spatiotemporal variability in dengue transmission intensity in Jakarta, Indonesia. PLoS Negl Trop Dis. 2020;14(3):e0008102. Epub 20200306. doi: 10.1371/journal.pntd.0008102. PubMed PMID: 32142516; PubMed Central PMCID: PMCPMC7080271.

13.	Djaafara B, Elyazar IRF, Surya A, Silalahi FSM, Handito A, Aryani D, et al. Estimating dengue force of infection from age-stratified surveillance data in Java, Indonesia. J R Soc Interface. 2025;22(232):20250445. Epub 20251126. doi: 10.1098/rsif.2025.0445. PubMed PMID: 41290141; PubMed Central PMCID: PMCPMC12646807.

14.	Rodriguez-Barraquer I, Cordeiro MT, Braga C, de Souza WV, Marques ET, Cummings DAT. From Re-Emergence to Hyperendemicity: The Natural History of the Dengue Epidemic in Brazil. PLOS Neglected Tropical Diseases. 2011;5(1):e935. doi: 10.1371/journal.pntd.0000935.

15.	Cattarino L, Rodriguez-Barraquer I, Imai N, Cummings DAT, Ferguson NM. Mapping global variation in dengue transmission intensity. Sci Transl Med. 2020;12(528):eaax4144. doi: 10.1126/scitranslmed.aax4144. PubMed PMID: 31996463.

16.	Castanha PM, Cordeiro MT, Martelli CM, Souza WV, Marques ET, Jr., Braga C. Force of infection of dengue serotypes in a population-based study in the northeast of Brazil. Epidemiol Infect. 2013;141(5):1080–8. Epub 20120717. doi: 10.1017/S0950268812001367. PubMed PMID: 22800513; PubMed Central PMCID: PMCPMC9151878.

17.	Rodriguez-Barraquer I, Buathong R, Iamsirithaworn S, Nisalak A, Lessler J, Jarman RG, et al. Revisiting Rayong: shifting seroprofiles of dengue in Thailand and their implications for transmission and control. Am J Epidemiol. 2014;179(3):353–60. Epub 20131105. doi: 10.1093/aje/kwt256. PubMed PMID: 24197388; PubMed Central PMCID: PMCPMC3895094.

18.	Phuong HT, Vy NHT, Thanh NTL, Tan M, de Bruin E, Koopmans M, et al. Estimating the force of infection of four dengue serotypes from serological studies in two regions of Vietnam. PLoS Negl Trop Dis. 2024;18(10):e0012568. Epub 20241007. doi: 10.1371/journal.pntd.0012568. PubMed PMID: 39374298; PubMed Central PMCID: PMCPMC11521262.

19.	Ferguson NM, Donnelly CA, Anderson RM. Transmission dynamics and epidemiology of dengue: insights from age-stratified sero-prevalence surveys. Philos Trans R Soc Lond B Biol Sci. 1999;354(1384):757–68. doi: 10.1098/rstb.1999.0428. PubMed PMID: 10365401; PubMed Central PMCID: PMCPMC1692557.

20.	Dirección General de Epidemiología. Manual de Procedimientos Estandarizados para la Vigilancia Epidemiológica de las Enfermedades Transmitidas por Vector (ETV). In: Secretaría de Salud SadPnyPndlS, editor. 2021.

21.	Centers for Disease Control and Prevention. CDC DENV-1-4 Real-Time RT-PCR Assay for Detection and Serotype Identification of Dengue Virus; Instructions for Use; Package Insert. 2025.

22.	Balde D, Ndiaye M, Efire AS, Faye O, Sall AA, Weidmann M, et al. CDC dengue typing kit fails to detect dengue virus 2 sylvatic genotype. Journal of Clinical Microbiology. 2025;63(8):e01741–24. doi: doi:10.1128/jcm.01741-24.

23.	Ramos-Castañeda J, Barreto dos Santos F, Martínez-Vega R, Galvão de Araujo JM, Joint G, Sarti E. Dengue in Latin America: Systematic Review of Molecular Epidemiological Trends. PLOS Neglected Tropical Diseases. 2017;11(1):e0005224. doi: 10.1371/journal.pntd.0005224.

24.	Reich NG, Shrestha S, King AA, Rohani P, Lessler J, Kalayanarooj S, et al. Interactions between serotypes of dengue highlight epidemiological impact of cross-immunity. J R Soc Interface. 2013;10(86):20130414. Epub 20130703. doi: 10.1098/rsif.2013.0414. PubMed PMID: 23825116; PubMed Central PMCID: PMCPMC3730691.

25.	Cattarino L, Rodriguez-Barraquer I, Imai N, Cummings DAT, Ferguson NM. Mapping global variation in dengue transmission intensity. Science Translational Medicine. 2020;12(528):eaax4144. doi: doi:10.1126/scitranslmed.aax4144.

26.	Katzelnick LC, Gresh L, Halloran ME, Mercado JC, Kuan G, Gordon A, et al. Antibody-dependent enhancement of severe dengue disease in humans. Science. 2017;358(6365):929–32. Epub 20171102. doi: 10.1126/science.aan6836. PubMed PMID: 29097492; PubMed Central PMCID: PMCPMC5858873.

27.	Burattini MN, Lopez LF, Coutinho FA, Siqueira JB, Jr., Homsani S, Sarti E, et al. Age and regional differences in clinical presentation and risk of hospitalization for dengue in Brazil, 2000-2014. Clinics (Sao Paulo). 2016;71(8):455–63. doi: 10.6061/clinics/2016(08)08. PubMed PMID: 27626476; PubMed Central PMCID: PMCPMC4975787.

28.	Narvaez F, Montenegro C, Juarez JG, Zambrana JV, Gonzalez K, Videa E, et al. Dengue severity by serotype and immune status in 19 years of pediatric clinical studies in Nicaragua. PLoS Negl Trop Dis. 2025;19(1):e0012811. Epub 20250110. doi: 10.1371/journal.pntd.0012811. PubMed PMID: 39792951; PubMed Central PMCID: PMCPMC11750095.

29.	Yung CF, Lee KS, Thein TL, Tan LK, Gan VC, Wong JGX, et al. Dengue serotype-specific differences in clinical manifestation, laboratory parameters and risk of severe disease in adults, singapore. Am J Trop Med Hyg. 2015;92(5):999–1005. Epub 20150330. doi: 10.4269/ajtmh.14-0628. PubMed PMID: 25825386; PubMed Central PMCID: PMCPMC4426593.

30.	Fried JR, Gibbons RV, Kalayanarooj S, Thomas SJ, Srikiatkhachorn A, Yoon IK, et al. Serotype-specific differences in the risk of dengue hemorrhagic fever: an analysis of data collected in Bangkok, Thailand from 1994 to 2006. PLoS Negl Trop Dis. 2010;4(3):e617. Epub 20100302. doi: 10.1371/journal.pntd.0000617. PubMed PMID: 20209155; PubMed Central PMCID: PMCPMC2830471.

31.	Wang L, Huang AT, Katzelnick LC, Lefrancq N, Escoto AC, Duret L, et al. Antigenic distance between primary and secondary dengue infections correlates with disease risk. Sci Transl Med. 2024;16(744):eadk3259. Epub 20240424. doi: 10.1126/scitranslmed.adk3259. PubMed PMID: 38657027; PubMed Central PMCID: PMCPMC12336440.

32.	Waggoner JJ, Balmaseda A, Gresh L, Sahoo MK, Montoya M, Wang C, et al. Homotypic Dengue Virus Reinfections in Nicaraguan Children. The Journal of Infectious Diseases. 2016;214(7):986–93. doi: 10.1093/infdis/jiw099.
