# Supplementary material for: Overall and serotype-specific dengue virus transmission intensity in Mexico, 2016–2023: A modelling study of case-notification data
Source: PLoS Med. 2026 Sep 3;23(9):e1004874. doi: 10.1371/journal.pmed.1004874 (PMC13541250; doi:10.1371/journal.pmed.1004874)
Supplement: Supplementary file 5 [file pmed.1004874.s007.docx]

**Second Response to Revisions Requests**
**Title**

- Please confirm that your title complies with PLOS Medicine's style. Your title must be nondeclarative and not a question. It should begin with main concept if possible. "Effect of" should be used only if causality can be inferred, i.e., for an RCT. Please place the study design ("A randomized controlled trial," "A retrospective study," "A modelling study," etc.) in the subtitle (ie, after a colon).

We have changed the title to:

*“Overall and serotype-specific dengue virus transmission intensity in Mexico, 2016-2023: a modelling study of case-notification data”*

**Abstract**

- Please confirm that your abstract complies with our requirements, including format (three sections: Background, Methods and Findings, and Conclusions) and providing all the information relevant to this study type <https://journals.plos.org/plosmedicine/s/submission-guidelines#loc-abstract>

With the additions set out below, and the addition of the total number of probable/confirmed cases to the sentence below, we think that the Abstract complies with PLOS Medicine requirements.

*“We analysed annual dengue case data reported to the National Epidemiological Surveillance System (SINAVE) in Mexico between 2016 and 2023, which comprised 833,630 probable or confirmed cases, using established catalytic models and a new, serotype-specific extension of these models that characterises differences in the transmission intensity of the different serotypes.”*

- In the Abstract Background section, please explain what is the open question that this study aims to address. Please clarify why this study is important at this time, and in Mexico, and/or how the study of Mexico fits into the global picture.

The Abstract Background section has been edited slightly and now reads as below.

*“Mexico, and the Americas region more widely, has experienced increased dengue incidence in recent years, and there are concerns that the changing climate may enhance dengue virus (DENV) transmission in the future. Whilst previous studies have characterised spatial heterogeneities in the long-term average risk of DENV infection in Mexico, the extent to which DENV transmission changes year-to-year has not been investigated. Furthermore, the extent to which DENV’s four serotypes (DENV-1, DENV-2, DENV-3 and DENV-4) may differ in their transmissibility remains poorly characterised, both in Mexico and globally.”*

- In the last sentence of the Abstract Methods and Findings section, please describe the main limitation(s) of the study's methodology.

We have added the following sentence:

*“The extent to which the serotype-specific transmission intensity estimates generated in this study are affected by potential heterogeneities in case reporting across states and through time remains to be validated in future studies.”*

- In the last sentence of the Abstract, please explain how understanding transmission of the 4 serotypes may help in the clinical or public health response.

We have added the following sentence to the Conclusions section of the Abstract:

*“The methods developed in this study contribute to the development of a better understanding of the past and current burden of dengue infection, which can help to refine assessments of the potential impact of existing and new interventions in future analyses.”*

- Please ensure that all abbreviations are defined at first use throughout the text.

We have slightly edited the sentence on lines 16-18 to make this clear. We believe all other abbreviations are defined on first use.

*“Furthermore, the extent to which DENV’s four serotypes (DENV-1, DENV-2, DENV-3 and DENV-4) may differ in their transmissibility remains poorly characterised globally.”*

- Please confirm that all numbers presented in the abstract are present and identical to numbers presented in the main manuscript text.

We can confirm that this is the case.

**Author Summary**

- In the author summary, in the final bullet point of 'What Do These Findings Mean?', please include the main limitations of the study in non-technical language.

We have removed the first bullet point from this section, as it is now covered by the new title of the paper, and have added the following sentence as a final bullet point:

*“The model developed in in this study assumes that dengue virus serotypes do not differ in their propensity to cause symptomatic disease, and that the observed serotype-specific data are representative of the serotypes circulating in the population. To date, this assumption remains to be validated in Mexico and globally.”*

**Introduction**

- Please ensure that the Introduction ends with a clear description of the study question or hypothesis.

We have changed the last sentence of the Introduction to improve clarity with respect to this. It now reads:

*“Finally, we developed a new serotype-specific catalytic model of DENV transmission intensity to quantify heterogeneities in the transmissibility of each serotype, enabling us to investigate potential drivers behind the incidence trends observed in Mexico over the last 8 years.”*

Lines 65-66: please correct grammar

*We have edited the sentence (now on lines 75-78) as below:*

*“The annual number of suspected dengue cases in the Americas, whilst variable, has increased steadily since 2000 [1], with estimates suggesting*

*that 16.7 (95% confidence interval [CI] [15.6, 17.7]) million infections and 7.6 (95% CI [4.8, 9.9]) million febrile cases occur on average each year in the region [2].”*

**Results**

- Beginning line 345: Please provide some additional context for the non-modeler reader.

We are not sure what this point refers to, as the sentence now on lines 363-366 contains results of data analysis, not modelling:

*“Regionally, the highest CFRs were recorded in states in north-west Mexico such as Baja California (0.591%; 3/508; 95% CI [0.122%, 1.72%]), Sonora (0.524%; 62/11843; 95% CI [0.402%, 0.671%]) and Chihuahua (0.929%; 3/323; 95% CI [0.192%, 2.69%]) (see Fig D in S1 Text).”*

- Line 399: please ensure that these acronyms are defined at first use.

We have verified that the three acronyms now in on line 420 are defined in lines 256-258.

**Funding statement**

- It appears that one or more study authors is affiliated with one or more of the agencies that funded the study. Thus, the statement “The funders had no role in study design, data collection and analysis, decision to publish, or preparation of the manuscript” does not apply. Please revise the Financial Disclosure accordingly, as in "[Author name] is [author's role] at [funding agency]. The funders had no other role in study design…..”

We have edited the funding statement to specify that OSS, AV and ID are affiliated with an MRC Centre that received funding from the MRC, but the funder of the Centre (which is the UK Medical Research Council) had no role in the study design, data collection and analysis, decision to publish, or preparation of the manuscript. We hope that our amendments clarify the confusion:

*“OSS, AV and ID acknowledge centre funding for the MRC Centre for Global Infectious Disease Analysis (reference MR/X020258/1), funded by the UK Medical Research Council (MRC, https://www.ukri.org/councils/mrc/). This UK funded award is carried out in the frame of the Global Health EDCTP3 Joint Undertaking. ID also acknowledges funding from Wellcome Trust (https://wellcome.org) (213494/Z/18/Z, 226072/Z/22/Z, 226727/Z/22/Z and 228185/Z/23/Z). The funders played no role in study design, data collection and analysis, decision to publish, or preparation of the manuscript."*

- The Data Availability Statement (DAS) requires revision as the statement that all data are freely available does not appear to be consistent with the statement in the Methods. For each data source used in your study:
  a) If the data are freely or publicly available, note this and state the location of the data: within the paper, in Supporting Information files, or in a public repository (include the DOI or accession number).
  b) If the data are owned by a third party but freely available upon request, please note this and state the owner of the data set and contact information for data requests (web or email address). Note that a study author cannot be the contact person for the data.
  c) If the data are not freely available, please describe briefly the ethical, legal, or contractual restriction that prevents you from sharing it. Please also include an appropriate contact (web or email address) for inquiries (again, this cannot be a study author).

Apologies for any confusion. The cleaned and anonymised data needed to run the model is available publicly from the GitHub repository at the link provided. However, as specified in the Methods, not all the underlying raw data that was used to create the estimates are available publicly online. The DAS statement has been updated as follows to reflect this:

*“The aggregated data and code needed to run the models can be accessed through GitHub, via the URL https://github.com/mrc-ide/Overall-and-Serotype-Specific-Dengue-Transmission-Trends-in-Mexico-2016-2023.*

*The individual-level data used to generate the aggregated data provided above are available partly publicly and partly upon request. Line-list dengue case data for 2020-2023 is available online from the Mexican Ministry of Health (https://www.gob.mx/salud/documentos/datos-abiertos-bases-historicas-de-enfermedades-transmitidas-por-vector). Data for 2016-2019 are available upon request from the Instituto Nacional de Acceso a la Información (INAI), folio 330026924000362. Population data for Mexico from the 2015 intercensal survey and 2020 census is available publicly from INEGI at https://en.www.inegi.org.mx/programas/intercensal/2015/ and https://en.www.inegi.org.mx/programas/ccpv/2020/.”*

- Please provide a DOI for your code and data.

We have now provided a DOI for the code (<https://doi.org/10.5281/zenodo.20561265>).

- Please include URLs for all funders.

URLs for both funders, the UK Medical Research Council (MRC) and the Wellcome Trust are provided in the Financial Disclosure statement:

*“OSS, AV and ID acknowledge funding from the MRC Centre for Global Infectious Disease Analysis (reference MR/X020258/1), funded by the UK Medical Research Council (MRC, https://www.ukri.org/councils/mrc/). This UK funded award is carried out in the frame of the Global Health EDCTP3 Joint Undertaking. ID also acknowledges funding from Wellcome Trust (https://wellcome.org) (213494/Z/18/Z, 226072/Z/22/Z, 226727/Z/22/Z and 228185/Z/23/Z).”*

**Ethics**

- Please specify whether informed consent was written or oral. Please ensure that the research complies with the PLOS policy in full: <https://journals.plos.org/plosmedicine/s/human-subjects-research#loc-patient-privacy-and-informed-consent-for-publication>. If consent was waived, please state in Methods.

We have added the sentences below to the Methods section under the heading “Ethics Statement”:

*“This study is a secondary analysis of aggregated, anonymized, publicly available data obtained from the National Epidemiological Surveillance System of the Mexican Ministry of Health. In accordance with the criteria of CIOMS-WHO Guideline 12 and Article 17 of local health legislation* [1, 2]*, the project is classified as research involving minimal risk (or no risk) and is therefore exempt from the requirement to obtain individual informed consent.”*

**Figures and Tables**

- Please convert any stacked bar charts to another data representation for example a table, or other type of graph.

Fig 6d (and correspondingly Figs H to AE in S1 Text) have been amended (see below).

Fig 6 from paper

- Please consider avoiding the use of red and green in order to make your figure more accessible

Where red and green are used in the same figure (Fig 1d, Fig 2, Fig 5, Fig 6b,d), the red and green colours used (hexes #DD5129 and #43B284) are colourblind friendly according to the R package *MetBrewer* (which in turn uses the R package *colorblindcheck* to check this). If this choice is not ideal, we are happy to revise the figures.

- Where data points are discrete, please ensure that they are depicted in the figures as discrete data and not as a continuous line.

We appreciate the suggestion and whilst the data in Fig 1a, d, Fig 2 and Fig 6b are discrete in nature as they are time-series data, for clarity and visualisation purposes it is beneficial to the reader to connect these points with a line. As regards Fig 6d, we use a solid, continuous line to show the model output (with shaded area showing the 95% credible interval), and discrete data points to show the data the model was fit to. We think visualising the model output and data in this way helps to differentiate between model output and the data.

- Please confirm that the appropriate usage rights apply to the use of this map. Please see our guidelines for map images: <https://journals.plos.org/plosmedicine/s/figures#loc-maps>

The maps in Figs 3, 4 and 5 in the paper (and all maps used in figures in the Supporting Information) used shapefiles from GADM (<https://gadm.org/>). We have added a link to the source of the base layer to all figure legends of figures that use maps.

**Acknowledgements and competing interests declaration**

- Please include an Acknowledgments section in your manuscript.

We have added the funding statement to the paper as the Acknowledgements section. If this results in a duplication of the statement, we can remove this. Otherwise, there are no other acknowledgements.

- All authors must declare their relevant competing interests per the PLOS policy, which can be seen here: https://journals.plos.org/plosmedicine/s/competing-interests For authors with ties to industry, please indicate whether any of the interests has a financial stake in the results of the current study.
- Please be advised that author AV has not completed the COI questionnaire.

AV should have now completed the COI questionnaire.

**Modeling studies**

- Please provide a diagram that shows the model structure, including how the disease natural history is represented, the process and determinants of disease acquisition, and how the putative intervention could affect the system.

We are not able to provide a diagram/flow chart to show the model structure. This is due to both the model’s complexity and to avoid misinterpretation (mechanistic models are commonly represented with a flow diagram, but the model structure here is catalytic).

- Please provide a complete list of model parameters, including clear and precise descriptions of the meaning of each parameter, together with the values or ranges for each, with justification or the primary source cited, and important caveats about the use of these values noted.

We set out the parameters used in the models as well as the prior distributions used, in the Mathematical Models section of Methods. We have also added a parameter table to S1 Text, Table C.

- Please provide a clear statement about how the model was fitted to the data including where relevant goodness-of-fit measure, the numerical algorithm used, which parameter varied, constraints imposed on parameter values, and starting conditions.

This is covered in Model Fitting subsection of Methods:

*“We fitted our models to the observed age-stratified case-notification data (Models 1-3) and to the age-stratified serotype-specific case incidence data reconstructed from the observed case-notification data (Model 4). We assume that the total number of cases (over the study period for Model 1 and over each year for Models 2-3) was Poisson distributed and that the age-specific number of cases was multinomially distributed. Similarly, for Model 4, we assume that the yearly total number of cases (over all age groups) for each serotype was Poisson distributed, and that for each serotype the yearly age-specific distribution of cases was multinomially distributed.*

*All models were fitted to the data using the Hamiltonian Monte Carlo method implemented in Stan (RStan version 2.32.7) [3, 4]. For each state, models were run using 3 chains, each with 6000 iterations, of which the first 1000 were considered warm-up. The chains were considered to have successfully converged if the diagnostic value Rhat was below 1.01 and the bulk effective sample size (ESS) was over 300 for all parameters, as per Stan guidelines [4].”*

- For uncertainty analyses, please state the sources of uncertainties quantified and not quantified this can include parameter, data, and model structure.

We have added the following sentence to Methods, lines 303-305:

*“We report the median of the posterior distribution of the estimated parameters and the 95% credible interval (CrI) to show the uncertainty.”*

- Please provide sensitivity analyses to identify which parameter values are most important in the model. Uncertainty estimates seek to derive a range of credible results on the basis of an exploration of the range of reasonable parameter values. The choice of method should be presented and justified.

This does not apply to our study, as we did not fix any parameter and rather estimated all parameters defining each model from the data.

- Please discuss the scientific rationale for this choice of model structure and identify points where this choice could influence conclusions drawn. Please also describe the strength of the scientific basis underlying the key model assumptions.

The rationale behind the chosen model structure is described in the Mathematical Models section of Methods (lines 217-237, 260-270), with some additional background information about DENV set out in the Introduction (e.g. lines 101-106, 125-127). The potential impact of various model assumptions on our results is covered in the Discussion (lines 542-564 and 600-607) and in this revision we have added additional comments throughout the manuscript following the Editorial suggestions.

## References

1.	Council for International Organizations of Medical Sciences (CIOMS). International Ethical Guidelines for Health-related Research Involving Humans 2016.

2.	Regulation of the General Health Law Regarding Research to Health, (2014).

3.	Stan Develpment Team. RStan: the R interface to Stan. 2.32.7 ed.

4.	Stan Develpment Team. Stan User's Guide. 2.38 ed2025.
