# Supplementary material for: Overall and serotype-specific dengue virus transmission intensity in Mexico, 2016–2023: A modelling study of case-notification data
Source: PLoS Med. 2026 Sep 3;23(9):e1004874. doi: 10.1371/journal.pmed.1004874 (PMC13541250; doi:10.1371/journal.pmed.1004874)
Supplement: Peer review history

# Editor report: Decision Letter 0

**DOI:** 10.1371/journal.pmed.1004874.r001

**Authors:** Helen Howard

9 Dec 2025

Dear Dr Simmons,

Thank you for submitting your manuscript entitled "Changes in dengue virus transmission in Mexico: 2016-2023" for consideration by PLOS Medicine.

Your manuscript has now been evaluated by the PLOS Medicine editorial staff, and I am writing to let you know that we would like to send your submission out for external peer review.

However, before we can send your manuscript to reviewers, we need you to complete your submission by providing the metadata that is required for full assessment. To this end, please login to Editorial Manager where you will find the paper in the 'Submissions Needing Revisions' folder on your homepage. Please click 'Revise Submission' from the Action Links and complete all additional questions in the submission questionnaire.

For clinical studies, please upload a copy of your trial study protocol as a supporting information file. The study protocol should be the version submitted for approval to the institutional review board or ethics committee, should include any amendments to the study protocol, as well as the date of their approval by the institutional review or ethics committee. Please also detail any deviations from the study protocol in the Methods section of your manuscript. The editors will consider the protocol and study conduct prior to a final decision for external review.

Please re-submit your manuscript within two working days, i.e. by Dec 11 2025 11:59PM.

Login to Editorial Manager here: https://www.editorialmanager.com/pmedicine

Once your full submission is complete, your paper will undergo a series of checks in preparation for peer review. Once your manuscript has passed all checks it will be sent out for review.

Feel free to email us at plosmedicine@plos.org if you have any queries relating to your submission.

Kind regards,

Helen Howard

PLOS Medicine

# Peer review: Decision Letter 1

**DOI:** 10.1371/journal.pmed.1004874.r002

**Authors:** Alison Farrell

15 Jan 2026

Dear Dr Simmons,

Many thanks for submitting your manuscript "Changes in dengue virus transmission in Mexico: 2016-2023" (PMEDICINE-D-25-04319R1) to PLOS Medicine. The paper has been reviewed by subject experts and a statistician; their comments are included below and can also be accessed here: [LINK]

As you will see, while the reviewers find the study of interest, they have raised numerous substantive concerns regarding the strength of some of the conclusions in the absence of serological data, lack of comparison of findings to existing literature, the need to consider the potential influence of the choice of RT-PCR test(s) as well as their low use, and require additional analyses to strengthen some of the claims. After discussing the paper with the editorial team and an academic editor with relevant expertise, I'm pleased to invite you to revise the paper in response to the reviewers' comments. We plan to send the revised paper to some or all of the original reviewers, and we cannot provide any guarantees at this stage regarding publication.

We further ask you to better frame the advance of the findings for a general audience, and their potential implications as they may relate to healthcare policy or implementation. Please also include a detailed discussion of the study limitations in the Discussion section (e.g. lack of serological data, potential limitations of conclusions based on heterogeneity of surveillance and reporting across states, and clarify whether there is any limitation to the inclusion of data on infections acquired outside the state of residence). Please identify the key novel findings in the Abstract.

In addition to these revisions, you may need to complete some formatting changes, which you will receive in a follow up email. A member of our team will be in touch with a set of requests shortly. If you do not receive a separate email within a few days, please assume that checks have been completed, and no additional changes are required.

When you upload your revision, please include a point-by-point response that addresses all of the reviewer and editorial points, indicating the changes made in the manuscript and either an excerpt of the revised text or the location (eg: page and line number) where each change can be found. Please also be sure to check the general editorial comments at the end of this letter and include these in your point-by-point response. When you resubmit your paper, please include a clean version of the paper as the main article file and a version with changes tracked as a marked-up manuscript. It may also be helpful to check the guidelines for revised papers at http://journals.plos.org/plosmedicine/s/revising-your-manuscript for any that apply to your paper.

We ask that you submit your revision by Feb 05 2026 11:59PM. However, if this deadline is not feasible, please contact me by email, and we can discuss a suitable alternative.

Don't hesitate to contact me directly with any questions (afarrell@plos.org).

Best regards,

Alison

Alison Farrell, Ph.D.

Senior Editor

PLOS Medicine

afarrell@plos.org

-----------------------------------------------------------

Comments from the academic editor:

1) I think there is clearly a large COVID effect on surveillance and potentially transmission, both overall, and for specific serotypes (e.g. Figure 1, 3, 4, and 5). The authors don't address this either in their model structures, or in interpretation of results, and I would like to see a little more consideration of this.

2) In model structures, there are quite a number of assumptions for fixed parameters - I suspect that reviewers will pick up on these, and will likely ask the author for more sensitivity analysis, varying these assumptions.

3) I tried to access the Github repository to review code and output, but this link is inaccessible currently. Important to review model convergence and diagnostics I think.

4) Minor point, but adding state labels to map figures would considerably aid interpretation of data for readers unfamiliar with the geography of Mexico.

5) The authors should acknowledge and discuss the limitation around non-availability of serological data in more detail.

-----------------------------------------------------------

Comments from the reviewers:

Reviewer #1: Simmons et al present a detailed set of dengue surveillance data from Mexico and several models to describe the transmission dynamics. As someone who is interested in dengue, I liked the detailed descriptions of the data and following the serotype trends. In particular, the re-emergence of DENV-3 (likely caused by the newly introduced and emerging DENV-3III_B.3.2 lineage) was fascinating the see. The figures were mostly clear and visually appealing.

My main criticism is that the work is mostly descriptive, and it is not very clear what we learn about dengue beyond 2016-2023 in Mexico. The conclusion mentioned that the work challenges the assumption of equal transmissibility between serotypes, but this knowledge gap or misconception was not well-established in the text (assuming that this comes from the Ferguson model, but my take on it is that it was for computational simplicity rather than a true epi process). Further, the discussion was also mostly descriptive and missed opportunities to compare the work with the vast dengue literature. I think that would help in making in more convincing to the reader the importance and novelty of the work.

A moderate concern is that the details of the RT-PCR test to determine the serotype should be provided. There are many assays, and some have different but important limitations (such as not being able to detect specific genotypes that could bias the data). Moreover, broadly calling for increased RT-PCR for serotype discrimination requires nuance as there are many ways to do this wrong and not enough clarity on which assays perform the best.

Minor concerns:

Should be clearer when referring to the disease/cases (dengue) and the virus (dengue virus). Descriptions of transmission, serotypes, and mosquito vectors should be attributed to the virus not the disease. This became better later in the text, but there are many examples of this misuse are throughout the abstract, first paragraph of the introduction, methods,

Introduction: is immunity truly life-long against reinfection with the same serotype. My assumption is that the duration would be long-lasting but also variable. Ref 11 does not study this and another reference should be added to support this claim.

Suggest labeling lambda in the figures as FOI for clarity.

I'm not sure how to make it better, but I found Figure 5a very difficult to follow. FOI, serotype, location, and year is a bit too much to pack into one set of panels. Perhaps something similar to Figure 3 would be better.

For the future, adding line numbers would help the review process. Many times I wanted to reference a specific sentence to be clearer with my responses but couldn't.

Reviewer #2: The article "Changes in dengue virus transmission in Mexico: 2016-2023", summarizes the spatial and temporal variation in dengue virus transmission at the serotype-specific level across Mexico from 2016 to 2023 using surveillance data. They found heterogeneity across Mexico with high transmission of DENV-1, DENV-2, limited circulation of DENV-4, and an increase in DENV-3 transmission in multiple states in recent years. This paper uses a large national dataset and addresses an important gap in the literature: how to measure the serotype-specific force of infection from case data. This paper is also important and relevant given the recent increase in dengue across South America. There are a few points of concern listed below, as well as some general suggestions.

Major Comments:

1) Introduction: "The extent to which primary and post-primary (including tertiary and quaternary) infections are reported to surveillance can also vary, not only due to differences in disease but also in surveillance". This sentence is confusing, and it is not clear that reference 12 supports this point. If differences in reporting of primary and secondary infections are not only due to disease presentation, or reporting by age (which is also accounted for in the models) what would drive this difference in surveillance? The sentence seems circular. A related point, in the Results section, "There was also substantial variation in the estimated secondary reporting rates (ρ) and the estimated reporting rates of primary cases relative to secondary reporting rates (γd), with ρ varying from 0.0051 (95% CrI 0.0046-0.0058) in Querétaro to 0.3245 (95% CrI 0.3207-0.3283) in Morelos, and γd from 0.0003 (95% CrI: 0.00001, 0.0018) in Coahuila to 0.9920 (95% CrI 0.9604-0.9997) in Chiapas." I am still unclear about the interpretation of variation in secondary reporting rates, and how this affects the FOI age assumptions. What could explain the variation across states reporting rates for primary versus secondary infection? Is it something to do with how severe cases are captured? Providing additional explanation for the possible causes would be helpful to the reader. Also, is data on IgM/IgG ratios available to help validate variation in primary versus secondary infections?

2) As written, it was not clear if all PCR-tested cases were serotyped. Further, as a reader, it wasn't clear until page 22 what proportion of cases were tested by PCR: "Among all cases, 85529 (10.3% of all probable or confirmed cases) tested RT-PCR positive for one of the four DENV serotypes between 2016 and 2023." Finally, what were the criteria for RT-PCR testing? Is it likely that they are representative, or are there biases in who was tested? Having more information on these points included in the methods section would he useful. It would also be helpful for the authors to explain the serotype-specific FOI estimates as conditional on assumptions about how representative the serotype-specific data are.

3) The authors assume that the serotype-specific and non-serotype-specific models have a constant level of dengue transmission before 2016. However, given what we know about long-term changes in dengue transmission in Mexico, including the changes that are related to urbanization and climate, does the assumption hold equally across all states in Mexico? This seems particularly problematic for serotypes that seem to not be observed for long periods at a time like DENV-3 and DENV-4: "This assumption also meant that if a serotype was not circulating between 2016- 2023, we assumed that it was also not circulating in the years before 2016, so any previous contribution of that serotype's historical circulation to population immunity profiles was not accounted for in the model, possibly biasing the initial susceptibility of the population and hence the FOI estimates generated."

4) The manuscript describes an increase in dengue transmission after COVID-19 but does not mention possible effects of the pandemic on surveillance and reporting. It would be helpful to comment on anything known about the effect on surveillance and possible effect it may have on estimates during the pandemic, especially 2020 and 2021.

5) This manuscript makes a novel contribution of estimating the time-varying serotype-specific force of infection from case data. It would be helpful to provide more literature review and comparison to what has been done previously for serotype-specific models using serology data.

6) Introduction: "Previous studies have suggested that the burden of dengue in Mexico may worsen further over the coming years due to climate change(7), although the extent and geographical distribution of the predicted change in dengue transmission differs between models(3, 8)." The present manuscript nicely measures annual changes in the force of infection of dengue across Mexico but does not connect to some of the bigger questions about effects of climate change. It would be helpful to return to this point and discuss potential implications in the discussion.

Minor comments:

1) It would be helpful to build slightly more intuition for what the force of infection is for non-expert readers when the concept is first introduced.

2) Throughout the manuscript, figures are not cited in order. For instance, in the methods, Figs. S5 and S6 are cited first in the text. Also, Fig. 2a is cited right after Fig. 1a. This is unconventional, and it would aid the reader if the figures were cited in the order they appear in the text.

3) Methods: "The explored four variants (Model 2-5) of the time-varying catalytic model developed by O'Driscoll et al." The heading to this section says "non-serotype specific models", but it was confusing because the four models were referred to as the 'explored four variants', which sounded like a serotype-specific model. Consider changing the wording to "Explored four different versions of the time varying catalytic model".

4) Methods: "We only reconstructed the serotype-specific case incidence in a given state for years that had more than 10 RT-PCR-positive test results, and we assumed that a DENV serotype was present in a state if there were more than 10 RT-PCR-positive results for that serotype in at least one of the modelled years (2016-2023 inclusive)." This is confusing. Does this mean if a state had 10 dengue cases, 2 DENV-1 and 8 DENV-2, then even though it meets the first rule, you wouldn't reconstruct the case data? It would be helpful to add a sentence to better explain when the serotype-reconstruction is performed.

5) Methods: "as we removed those under 1 year old from the analysis". Further down in the text, it is explained that the authors removed "children younger than 1 year old due to maternally transferred immunity". It would be helpful if this point could be added further up in the text when this is first introduced.

6) Models: Why introduce the scaling factor in equation 11 for different reporting rates by age and not further up in equation 2, when the concept is first introduced?

7) What are the colors in Fig. 5b?

8) "We were able to reconstruct the estimated age-distribution of the serotype-specific data (see Methods) using the serotype-specific model in 23 out of 26 states." It would be helpful to list the 3 excluded states. It wasn't clear why some were excluded that had high numbers of cases.

Reviewer #3: See attachment

Michael Dewey

Reviewer #4: This study characterizes the spatial and temporal variations in dengue force of infection (FOI) across 27 states in Mexico from 2016 to 2023, utilizing confirmed/probable annual dengue cases (total of 833761) reported to the surveillance system and previously established non-serotype-based time-constant and time-varying catalytic models. Authors also report variations of the catalytic model they developed to allow serotype-specific time-varying forces, using the 85529 RT-PCR-positive cases to characterise differences in FOI across dengue serotypes. Overall, the manuscript is well written, and the descriptive analyses are well presented in Figures 1 and 3. While I believe the manuscript is valuable and worthy of publication in PLOS Medicine, I have also noted several limitations that must be addressed.

Their findings from the data modeling are undoubtedly the best use of the available RT-PCR data. However, it may not inform the actual epidemiological situation of dengue in Mexico. This is because the non-serotype-based time-constant and time-varying catalytic models used in the manuscript rely on presumptive and RT-PCR data, rather than serological testing, and on age-based stratification. The inability to distinguish primary from secondary infections and the exclusion of asymptomatic and mild subclinical cases can also lead to an underestimation of the true infection rate. While the models employed may still provide valuable insights into symptomatic cases (laboratory-confirmed and presumptive), the findings are not fully representative of the overall epidemiological situation of dengue, and this must be acknowledged.

Additionally, the non-serotype version of the catalytic models assumes that the four DENV serotypes are equally transmissible. This poses a significant limitation as asymptomatic dengue infections can vary by serotype. Previous, mostly cohort-based, studies showed that DENV-1 and DENV-4 are more likely to be asymptomatic, whereas DENV-2 is frequently symptomatic. However, these patterns may also vary depending on previous immunity, population genetics, and local viral strains. I appreciate that the authors acknowledged these limitations and, indeed, attempted to improve these models by developing serotype-specific variants using serotype-specific RT-PCR results. Yet it is the "best use of the existing data" scenario, but the findings reported remain limited by the lack of serology data.

Authors stated that "we assumed that the serotypes of distribution of the cases with unknown hospitalization status were the same as was observed among non-hospitalized cases." I believe this assumption can be challenged because DENV2 transmission following the ZIKV wave in the Pacific has driven more hospitalizations than historical DENV2 transmission. Additionally, genotypic variation among dengue serotypes can disproportionately influence disease severity, as observed during the circulation of American and Asian/American DENV2 in Peru following a DENV1 outbreak. Considering this, can the authors compare the serotype of distribution among hospitalized and non-hospitalized symptomatic cases?

The authors claimed that their model predicted that DENV-1 and DENV-2 circulated at high levels, with DENV-4 transmission intensity low during 2016 to 2023 across Mexico. They also noted that DENV-3 transmission intensity has increased in some Mexican states over the past few years. Given the lack of serological data, the authors should limit their findings to symptomatic RT-PCR-confirmed cases.

Additionally, I suggest a modification in the abstract that states "This work quantifies serotype-specific differences in dengue transmission intensity using routinely collected case-notification data and demonstrates how extensive RT-PCR testing and new rapid diagnostic tests capable of discerning infecting serotypes can help to better understand the contributions of different serotypes to transmission." I am particularly concerned about the use of RT-PCR to estimate transmission rates, given that dengue viremia is brief, is estimated to miss roughly 50% of symptomatic cases, and the asymptomatic infections are also unaccounted for.

Authors mention that "Serotype-specific seroprevalence studies would be instrumental to disentangle the potential mechanisms behind the heterogeneities in serotype circulation." It would be beneficial to add a discussion of the serological tools that could power future studies like theirs. They should emphasize that performing serology on an acute specimen is the best way to identify past infection and can distinguish primary from secondary dengue symptomatic infections. Serosurveillance using serological assays capable of distinguishing primary dengue serotypes with convenient yearly sampling can cover both asymptomatic and symptomatic dengue infections and distinguish primary from secondary dengue infections. Acknowledging the existence of such serological tools could also be beneficial for advancing the field.

Additionally, previous studies have noted that homologous reinfection and heterologous asymptomatic infections can contribute to DENV transmission, as infected individuals with high viremia may facilitate transmission. Authors should acknowledge that future epidemiological models should incorporate homologous re-infection and heterologous inapparent infections to more accurately estimate DENV exposure and transmission.

Any attachments provided with reviews can be seen via the following link: [LINK]

--------------------------------------------------------- ---

General editorial requests:

(Note: not all will apply to your paper, but please check each item carefully)

*We ask every co-author listed on the manuscript to fill in a contributing author statement, making sure to declare all competing interests. If any of the co-authors have not filled in the statement, we will remind them to do so when the paper is revised. If all statements are not completed in a timely fashion this could hold up the re-review process. If new competing interests are declared later in the revision process, this may also hold up the submission. Should there be a problem getting one of your co-authors to fill in a statement we will be in contact. Please do not add or remove authors without first discussing this with the handling editor. You can see our competing interests policy here: http://journals.plos.org/plosmedicine/s/competing-interests.

*Please upload any figures associated with your paper as individual TIF or EPS files with 300dpi resolution at resubmission; please read our figure guidelines for more information on our requirements: http://journals.plos.org/plosmedicine/s/figures. While revising your submission, we strongly recommend that you use PLOS's NAAS tool (https://ngplosjournals.pagemajik.ai/artanalysis) to test your figure files. NAAS can convert your figure files to the TIFF file type and meet basic requirements (such as print size, resolution), or provide you with a report on issues that do not meet our requirements and that NAAS cannot fix.

After uploading your figures to PLOS's NAAS tool - https://ngplosjournals.pagemajik.ai/artanalysis, NAAS will process the files provided and display the results in the "Uploaded Files" section of the page as the processing is complete.

If the uploaded figures meet our requirements (or NAAS is able to fix the files to meet our requirements), the figure will be marked as "fixed" above. If NAAS is unable to fix the files, a red "failed" label will appear above.

When NAAS has confirmed that the figure files meet our requirements, please download the file via the download option, and include these NAAS processed figure files when submitting your revised manuscript.

*Please ensure that the paper adheres to the PLOS Data Availability Policy (see http://journals.plos.org/plosmedicine/s/data-availability), which requires that all data underlying the study's findings be provided in a repository or as Supporting Information. For data residing with a third party, authors are required to provide instructions with contact information (web or email address) for obtaining the data. Please note that a study author cannot be the contact person for the data. PLOS journals do not allow statements supported by "data not shown" or "unpublished results." For such statements, authors must provide supporting data or cite public sources that include it.

*We expect all researchers with submissions to PLOS in which author-generated code underpins the findings in the manuscript to make all author-generated code available without restrictions upon publication of the work. In cases where code is central to the manuscript, we may require the code to be made available as a condition of publication. Authors are responsible for ensuring that the code is reusable and well documented. Please make any custom code available, either as part of your data deposition or as a supplementary file. Please add a sentence to your data availability statement regarding any code used in the study, e.g. "The code used in the analysis is available from Github [URL] and archived in Zenodo [DOI link]" Please review our guidelines at https://journals.plos.org/plosmedicine/s/materials-software-and-code-sharing and ensure that your code is shared in a way that follows best practice and facilitates reproducibility and reuse. Because Github depositions can be readily changed or deleted, we encourage you to make a permanent DOI'd copy (e.g. in Zenodo) and provide the URL.

*Please complete PLOS’ questionnaire on inclusivity in global research (https://journals.plos.org/plosmedicine/s/best-practices-in-research-reporting)

*Please ensure that the study is reported according to the appropriate guideline and include the completed checklist(s) as Supporting Information. When completing the checklist, please use section and paragraph numbers, rather than page numbers. Please add the following statement, or similar, to the Methods: "This study is reported as per [XXXX] guideline (S1 Checklist).

FORMATTING - GENERAL

*Abstract: Please structure your abstract using the PLOS Medicine headings (Background, Methods and Findings, Conclusions). Please combine the Methods and Findings sections into one section. The last sentence of the Methods and Findings section should describe the methodological limitations of the study.

*At this stage, we ask that you include a short, non-technical Author Summary of your research to make findings accessible to a wide audience that includes both scientists and non-scientists. The Author Summary should immediately follow the Abstract in your revised manuscript. This text is subject to editorial change and should be distinct from the scientific abstract. Ideally each sub-heading should contain 2-3 single sentence, concise bullet points containing the most salient points from your study. In the final bullet point of 'What Do These Findings Mean?', please include the main limitations of the study in non-technical language. Please see our author guidelines for more information: https://journals.plos.org/plosmedicine/s/revising-your-manuscript#loc-author-summary.

*Please express the main results with 95% CIs as well as p values. When reporting p values please report as p<0.001 and where higher as the exact p value p=0.002, for example. Throughout, suggest reporting statistical information as follows to improve clarity for the reader "22% (95% CI [13%,28%]; p</=)". Please be sure to define all numerical values at first use.

*Please include page numbers and line numbers in the manuscript file. Use continuous line numbers (do not restart the numbering on each page).

*Please cite the reference numbers in square brackets. Citations should precede punctuation.

FIGURES AND TABLES

*Please provide titles and legends for all figures and tables (including those in Supporting Information files).

*Please define all abbreviations used in each figure/table (including those in Supporting Information files).

*Please consider avoiding the use of red and green in order to make your figure more accessible to those with color blindness.

SUPPLEMENTARY MATERIAL

*Please note that supplementary material will be posted as supplied by the authors. Therefore, please amend it according to the relevant comments outlined here.

*Please cite your Supporting Information as outlined here: https://journals.plos.org/plosmedicine/s/supporting-information

REFERENCES

*PLOS uses the numbered citation (citation-sequence) method and first six authors, et al.

*Please ensure that journal name abbreviations match those found in the National Center for Biotechnology Information (NCBI) databases (http://www.ncbi.nlm.nih.gov/nlmcatalog/journals), and are appropriately formatted and capitalised.

*Where website addresses are cited, please include the complete URL and specify the date of access (e.g. [accessed: 12/06/2023]).

*Please also see https://journals.plos.org/plosmedicine/s/submission-guidelines#loc-references for further details on reference formatting.

OBSERVATIONAL STUDIES

*Abstract: Please include the study design, population and setting, number of participants, years during which the study took place (enrollment and follow up), length of follow up, and main outcome measures.

*Please ensure that the study is reported according to the STROBE (or appropriate STOBE extension) guideline (available from: https://www.equator-network.org/reporting-guidelines/strobe) and include the completed STROBE (or STROBE extension) checklist as Supporting Information. Please add the following statement, or similar, to the Methods: "This study is reported as per the Strengthening the Reporting of Observational Studies in Epidemiology (STROBE) guideline (S1 Checklist)." When completing the checklist, please use section and paragraph numbers, rather than page numbers.

*[FOR POPULATION HEALTH/REGISTRY STUDIES] Please ensure that the study is reported according to the RECORD guideline (available from https://www.record-statement.org) and include the completed checklist as Supporting Information. Please add the following statement, or similar, to the Methods: "This study is reported as per the Reporting of Studies Conducted using Observational Routinely-Collected Data (RECORD) guideline (S1 Checklist)." When completing the checklist, please use section and paragraph numbers, rather than page numbers.

*[FOR POPULATION HEALTH ESTIMATES] Please ensure that the study is reported according to the GATHER statement (available from https://www.equator-network.org/reporting-guidelines/gather-statement) and include the completed checklist as Supporting Information. Please add the following statement, or similar, to the Methods: "This study is reported as per the Guidelines for Accurate and Transparent Health Estimates Reporting (GATHER) statement (S1 Checklist)." When completing the checklist, please use section and paragraph numbers, rather than page numbers.

*For all observational studies, in the manuscript text, please indicate: (1) the specific hypotheses you intended to test, (2) the analytical methods by which you planned to test them, (3) the analyses you actually performed, and (4) when reported analyses differ from those that were planned, transparent explanations for differences that affect the reliability of the study's results. If a reported analysis was performed based on an interesting but unanticipated pattern in the data, please be clear that the analysis was data driven.

*Please state in the Methods section whether the study had a prospective protocol or analysis plan. If a prospective analysis plan (from your funding proposal, IRB or other ethics committee submission, study protocol, or other planning document written before analyzing the data) was used in designing the study, please include the relevant document(s) with your revised manuscript as a Supporting Information file to be published alongside your study and cite it in the Methods section. A legend for this file should be included at the end of your manuscript. If no such document exists, please make sure that the Methods section transparently describes when analyses were planned, and when/why any data-driven changes to analyses took place. Changes in the analysis, including those made in response to peer review comments, should be identified as such in the Methods section of the paper, with rationale.

MODELLING STUDIES

The following list is derived from Geoffrey P Garnett, Simon Cousens, Timothy B Hallett, Richard Steketee, Neff Walker. Mathematical models in the evaluation of health programmes. (2011) Lancet DOI:10.1016/S0140-6736(10)61505-X:

*If pertinent, please provide a diagram that shows the model structure, including how the natural history of the disease is represented, the process and determinants of disease acquisition, and how the putative intervention could affect the system.

*Please provide a complete list of model parameters, including clear and precise descriptions of the meaning of each parameter, together with the values or ranges for each, with justification or the primary source cited and important caveats about the use of these values noted.

*Please provide a clear statement about how the model was fitted to the data, including goodness-of-fit measure, the numerical algorithm used, which parameter varied, constraints imposed on parameter values, and starting conditions.

*For uncertainty analyses, please state the sources of uncertainties quantified and not quantified [can include parameter, data, and model structure].

*Please provide sensitivity analyses to identify which parameter values are most important in the model. Uncertainty estimates seek to derive a range of credible results on the basis of an exploration of the range of reasonable parameter values. The choice of method should be presented and justified.

*Please discuss the scientific rationale for the choice of model structure and identify points where this choice could influence conclusions drawn. Please also describe the strength of the scientific basis underlying the key model assumptions.

*For studies that develop a prediction model or evaluate its performance, please ensure that the study is reported according to the TRIPOD statement (https://www.equator-network.org/reporting-guidelines/tripod-statement) and include the completed checklist as Supporting Information. Please add the following statement, or similar, to the Methods: "This study is reported as per the Transparent Reporting of a Multivariable Prediction Model for Individual Prognosis Or Diagnosis (TRIPOD) statement (S1 Checklist)." For studies using machine learning, please use the TRIPOD-AI checklist. When completing the checklist, please use section and paragraph numbers, rather than page numbers.

Attachment Submitted filename: simmons.pdf

# Author comment: Author response to Decision Letter 2

**DOI:** 10.1371/journal.pmed.1004874.r003

31 Mar 2026

Attachment Submitted filename: PLOS Revisions Letter.docx

# Peer review: Decision Letter 2

**DOI:** 10.1371/journal.pmed.1004874.r004

**Authors:** Alison Farrell

12 May 2026

Dear Dr. Simmons,

Thank you very much for re-submitting your manuscript "Overall and serotype-specific trends in dengue virus transmission in Mexico, 2016-2023" (PMEDICINE-D-25-04319R2) for review by PLOS Medicine.

I have discussed the paper with my colleagues and the academic editor and it was also seen again by 4 reviewers. I am pleased to say that provided the remaining editorial and production issues are dealt with we are planning to accept the paper for publication in the journal.

The remaining issues that need to be addressed are listed at the end of this email. Any accompanying reviewer attachments can be seen via the link below. Please take these into account before resubmitting your manuscript:

[LINK]

***Please note while forming your response, if your article is accepted, you may have the opportunity to make the peer review history publicly available. The record will include editor decision letters (with reviews) and your responses to reviewer comments. If eligible, we will contact you to opt in or out.***

In revising the manuscript for further consideration here, please ensure you address the specific points made by each reviewer and the editors. In your rebuttal letter you should indicate your response to the reviewers' and editors' comments and the changes you have made in the manuscript. Please submit a clean version of the paper as the main article file. A version with changes marked must also be uploaded as a marked up manuscript file.

Please also check the guidelines for revised papers at http://journals.plos.org/plosmedicine/s/revising-your-manuscript for any that apply to your paper. If you haven't already, we ask that you provide a short, non-technical Author Summary of your research to make findings accessible to a wide audience that includes both scientists and non-scientists. The Author Summary should immediately follow the Abstract in your revised manuscript. This text is subject to editorial change and should be distinct from the scientific abstract.

In addition to these revisions, you may need to complete some formatting changes, which you will receive in a follow up email. A member of our team will be in touch with a set of requests shortly. If you do not receive a separate email within a few days, please assume that checks have been completed, and no additional changes are required.

We expect to receive your revised manuscript within 1 week. Please email us (plosmedicine@plos.org) if you have any questions or concerns.

We ask every co-author listed on the manuscript to fill in a contributing author statement. If any of the co-authors have not filled in the statement, we will remind them to do so when the paper is revised. If all statements are not completed in a timely fashion this could hold up the re-review process. Should there be a problem getting one of your co-authors to fill in a statement we will be in contact. YOU MUST NOT ADD OR REMOVE AUTHORS UNLESS YOU HAVE ALERTED THE EDITOR HANDLING THE MANUSCRIPT TO THE CHANGE AND THEY SPECIFICALLY HAVE AGREED TO IT.

Please ensure that the paper adheres to the PLOS Data Availability Policy (see http://journals.plos.org/plosmedicine/s/data-availability), which requires that all data underlying the study's findings be provided in a repository or as Supporting Information. For data residing with a third party, authors are required to provide instructions with contact information for obtaining the data. PLOS journals do not allow statements supported by "data not shown" or "unpublished results." For such statements, authors must provide supporting data or cite public sources that include it.

To enhance the reproducibility of your results, we recommend that you deposit your laboratory protocols in protocols.io, where a protocol can be assigned its own identifier (DOI) such that it can be cited independently in the future. Additionally, PLOS ONE offers an option to publish peer-reviewed clinical study protocols. Read more information on sharing protocols at https://plos.org/protocols?utm_medium=editorial-email&utm_source=authorletters&utm_campaign=protocols

Please review your reference list to ensure that it is complete and correct. If you have cited papers that have been retracted, please include the rationale for doing so in the manuscript text, or remove these references and replace them with relevant current references. Any changes to the reference list should be mentioned in the rebuttal letter that accompanies your revised manuscript.

Please note, when your manuscript is accepted, an uncorrected proof of your manuscript will be published online ahead of the final version, unless you've already opted out via the online submission form. If, for any reason, you do not want an earlier version of your manuscript published online or are unsure if you have already indicated as such, please let the journal staff know immediately at plosmedicine@plos.org.

If you have any questions in the meantime, please contact me or the journal staff on plosmedicine@plos.org.

We look forward to receiving the revised manuscript by May 19 2026 11:59PM.

Sincerely,

Alison Farrell, Ph.D.

Senior Editor

PLOS Medicine

plosmedicine.org

------------------------------------------------------------

Requests from Editors:

Title

* Please confirm that your title complies with PLOS Medicine's style. Your title must be nondeclarative and not a question. It should begin with main concept if possible. "Effect of" should be used only if causality can be inferred, i.e., for an RCT. Please place the study design ("A randomized controlled trial," "A retrospective study," "A modelling study," etc.) in the subtitle (ie, after a colon).

Abstract

* Please confirm that your abstract complies with our requirements, including format (three sections: Background, Methods and Findings, and Conclusions) and providing all the information relevant to this study type https://journals.plos.org/plosmedicine/s/submission-guidelines#loc-abstract

* In the Abstract Background section, please explain what is the open question that this study aims to address. Please clarify why this study is important at this time, and in Mexico, and/or how the study of Mexico fits into the global picture.

* In the last sentence of the Abstract Methods and Findings section, please describe the main limitation(s) of the study's methodology.

* In the last sentence of the Abstract, please explain how understanding transmission of the 4 serotypes may help in the clinical or public health response.

* Please ensure that all abbreviations are defined at first use throughout the text.

* Please confirm that all numbers presented in the abstract are present and identical to numbers presented in the main manuscript text.

Author Summary

* In the author summary, in the final bullet point of 'What Do These Findings Mean?', please include the main limitations of the study in non-technical language.

Introduction

* Please ensure that the Introduction ends with a clear description of the study question or hypothesis.

*Lines 65-66: please correct grammar

Results

* Beginning line 345: Please provide some additional context for the non-modeler reader.

* Line 399: please ensure that these acronyms are defined at first use.

Funding statement

* It appears that one or more study authors is affiliated with one or more of the agencies that funded the study. Thus, the statement “The funders had no role in study design, data collection and analysis, decision to publish, or preparation of the manuscript” does not apply. Please revise the Financial Disclosure accordingly, as in "[Author name] is [author's role] at [funding agency]. The funders had no other role in study design…..”

* The Data Availability Statement (DAS) requires revision as the statement that all data are freely available does not appear to be consistent with the statement in the Methods. For each data source used in your study:

a) If the data are freely or publicly available, note this and state the location of the data: within the paper, in Supporting Information files, or in a public repository (include the DOI or accession number).

b) If the data are owned by a third party but freely available upon request, please note this and state the owner of the data set and contact information for data requests (web or email address). Note that a study author cannot be the contact person for the data.

c) If the data are not freely available, please describe briefly the ethical, legal, or contractual restriction that prevents you from sharing it. Please also include an appropriate contact (web or email address) for inquiries (again, this cannot be a study author).

**Please provide a DOI for your code and data.

**Please include URLs for all funders.

Ethics

* Please specify whether informed consent was written or oral. Please ensure that the research complies with the PLOS policy in full: https://journals.plos.org/plosmedicine/s/human-subjects-research#loc-patient-privacy-and-informed-consent-for-publication

If consent was waived, please state in Methods.

Figures and Tables

* Please convert any stacked bar charts to another data representation for example a table, or other type of graph.

* Please consider avoiding the use of red and green in order to make your figure more accessible

* Where data points are discrete, please ensure that they are depicted in the figures as discrete data and not as a continuous line.

* Please confirm that the appropriate usage rights apply to the use of this map. Please see our guidelines for map images: https://journals.plos.org/plosmedicine/s/figures#loc-maps

Acknowledgements and competing interests declaration

* Please include an Acknowledgments section in your manuscript.

* All authors must declare their relevant competing interests per the PLOS policy, which can be seen here: https://journals.plos.org/plosmedicine/s/competing-interests For authors with ties to industry, please indicate whether any of the interests has a financial stake in the results of the current study.

* Please be advised that author AV has not completed the COI questionnaire.

Modeling studies

* Please provide a diagram that shows the model structure, including how the disease natural history is represented, the process and determinants of disease acquisition, and how the putative intervention could affect the system.

* Please provide a complete list of model parameters, including clear and precise descriptions of the meaning of each parameter, together with the values or ranges for each, with justification or the primary source cited, and important caveats about the use of these values noted.

* Please provide a clear statement about how the model was fitted to the data including where relevant goodness-of-fit measure, the numerical algorithm used, which parameter varied, constraints imposed on parameter values, and starting conditions.

* For uncertainty analyses, please state the sources of uncertainties quantified and not quantified this can include parameter, data, and model structure.

* Please provide sensitivity analyses to identify which parameter values are most important in the model. Uncertainty estimates seek to derive a range of credible results on the basis of an exploration of the range of reasonable parameter values. The choice of method should be presented and justified.

* Please discuss the scientific rationale for this choice of model structure and identify points where this choice could influence conclusions drawn. Please also describe the strength of the scientific basis underlying the key model assumptions.

For more details on the requests above please see Geoffrey P Garnett, Simon Cousens, Timothy B Hallett, Richard Steketee, Neff Walker. Mathematical models in the evaluation of health programmes. (2011) Lancet DOI:10.1016/S0140-6736(10)61505-X.

Comments from Reviewers:

Reviewer #1: I thank the authors for their effort in addressing all of the reviewer's comments. While I cannot say if all of the comments have been adequately addressed, mine are. I look forward to seeing this published.

Reviewer #2: The authors have made a concerted effort to address my comments and concerns. Where they could not address the comments with further analyses, they have appropriately acknowledged the limitations in the discussion.

Reviewer #3: The authors have addressed all my points

Michael Dewey

Reviewer #4: The authors have adequately addressed my previous comments. I do not have further comments.

Any attachments provided with reviews can be seen via the following link:

[LINK]

# Author comment: Author response to Decision Letter 3

**DOI:** 10.1371/journal.pmed.1004874.r005

8 Jun 2026

Attachment Submitted filename: Second Response to Revisions Requests.docx

# Editor report: Decision Letter 3

**DOI:** 10.1371/journal.pmed.1004874.r006

**Authors:** Alison Farrell

9 Jun 2026

Dear Dr Simmons,

On behalf of my colleagues and the Academic Editor, Peter MacPherson, I am pleased to inform you that we have agreed to publish your manuscript "Overall and serotype-specific dengue virus transmission intensity in Mexico, 2016-2023: a modelling study of case-notification data" (PMEDICINE-D-25-04319R3) in PLOS Medicine.

Before your manuscript can be formally accepted you will need to complete some formatting changes, which you will receive in a follow up email. Please be aware that it may take several days for you to receive this email; during this time no action is required by you. Once you have received these formatting requests, please note that your manuscript will not be scheduled for publication until you have made the required changes.

In the meantime, please log into Editorial Manager at http://www.editorialmanager.com/pmedicine/, click the "Update My Information" link at the top of the page, and update your user information to ensure an efficient production process.

PRESS

We frequently collaborate with press offices. If your institution or institutions have a press office, please notify them about your upcoming paper at this point, to enable them to help maximise its impact. If the press office is planning to promote your findings, we would be grateful if they could coordinate with medicinepress@plos.org. If you have not yet opted out of the early version process, we ask that you notify us immediately of any press plans so that we may do so on your behalf.

We also ask that you take this opportunity to read our Embargo Policy regarding the discussion, promotion and media coverage of work that is yet to be published by PLOS. As your manuscript is not yet published, it is bound by the conditions of our Embargo Policy. Please be aware that this policy is in place both to ensure that any press coverage of your article is fully substantiated and to provide a direct link between such coverage and the published work. For full details of our Embargo Policy, please visit http://www.plos.org/about/media-inquiries/embargo-policy/.

To enhance the reproducibility of your results, we recommend that you deposit your laboratory protocols in protocols.io, where a protocol can be assigned its own identifier (DOI) such that it can be cited independently in the future. Additionally, PLOS ONE offers an option to publish peer-reviewed clinical study protocols. Read more information on sharing protocols at https://plos.org/protocols?utm_medium=editorial-email&utm_source=authorletters&utm_campaign=protocols

Thank you again for submitting to PLOS Medicine. We look forward to publishing your paper.

Sincerely,

Alison Farrell, Ph.D.

Senior Editor

PLOS Medicine
